# Correlated product of experts for sparse Gaussian process regression

**DOI:** 10.1007/s10994-022-06297-3

**Published:** 2023-01-25

**Authors:** Manuel Schürch, Dario Azzimonti, Alessio Benavoli, Marco Zaffalon

**Affiliations:** 1 Istituto Dalle Molle di Studi sull’Intelligenza Artificiale (IDSIA), Lugano, Switzerland; 2 Università della Svizzera italiana (USI), Lugano, Switzerland; 3 University of Limerick (UL), Limerick, Ireland

**Keywords:** Gaussian processes, Probabilistic regression, Expert fusion

## Abstract

Gaussian processes (GPs) are an important tool in machine learning and statistics. However, off-the-shelf GP inference procedures are limited to datasets with several thousand data points because of their cubic computational complexity. For this reason, many sparse GPs techniques have been developed over the past years. In this paper, we focus on GP regression tasks and propose a new approach based on aggregating predictions from several local and correlated experts. Thereby, the degree of correlation between the experts can vary between independent up to fully correlated experts. The individual predictions of the experts are aggregated taking into account their correlation resulting in consistent uncertainty estimates. Our method recovers independent Product of Experts, sparse GP and full GP in the limiting cases. The presented framework can deal with a general kernel function and multiple variables, and has a time and space complexity which is linear in the number of experts and data samples, which makes our approach highly scalable. We demonstrate superior performance, in a time vs. accuracy sense, of our proposed method against state-of-the-art GP approximations for synthetic as well as several real-world datasets with deterministic and stochastic optimization.

**Supplementary Information:**

The online version contains supplementary material available at 10.1007/s10994-022-06297-3.

## Introduction

*Gaussian processes* (GPs) are a class of powerful probabilistic method used in many statistical models due to their modelling flexibility, robustness to overfitting and availability of well-calibrated predictive uncertainty estimates with many applications in machine learning and statistics. However, off-the-shelf GP inference procedures are limited to datasets with a few thousand data points *N*, because of their computational complexity $\mathcal {O}(N^3)$ and memory complexity $\mathcal {O}(N^2)$ due to the inversion of a $N \times N$ kernel matrix (Rasmussen and Williams 2006). For this reason, many GP approximation techniques have been developed over the past years. There are at least two different approaches to circumvent the computational limitation of full GP. On the one hand, there are *sparse and global* methods (Csató and Opper 2002, Quiñonero-Candela and Rasmussen 2005, Rasmussen and Williams 2006, Seeger et al. 2003) based on $M_g \ll N$ so-called (global) inducing points, which cover sparsely the input space and optimally summarizing the dependencies of the training points. This results in a low-rank approximation of the kernel matrix of size $M_g\times M_g$, which is less expensive to invert. These methods consistently approximate full GP, for instance the authors in Titsias (2009) have shown that it converges to full GP as $M_g \rightarrow N$. However, all these methods are still cubic in the number of global inducing points $M_g$ and for many applications—in particular in higher dimensions—the amount of inducing points has to be rather large to capture the pattern of the function properly. A lot of work has been done to optimize the locations of the inducing inputs e.g., Bui et al. (2017), Snelson and Ghahramani (2006), Titsias (2009), which allows to have less inducing points but more optimization parameters. This optimization procedures were further improved by stochastic optimization e.g., Bui et al. (2017), Hensman et al. (2013), Kania et al. (2021), Schürch et al. (2020), which allows to update the parameters in mini-batches and thus speed up the inference. Optimization of these (variational) parameters helps to scale GP approximations, however, the large number of optimization parameters makes these methods hard to train and they are still limited to $M_g$ global inducing points.

On the other hand, there are *independent and local * models based on averaging predictions from *J* independent local experts/models resulting in a block-diagonal approximation of the kernel matrix. The final probabilistic aggregation is then based on a product of the individual predictive densities, thus they are called *Product of Experts (PoEs)*, see Fleet (2014), Deisenroth and Ng (2015), Hinton (2002), Rullière et al. (2018), Tresp (2000), Liu et al. (2018). PoE methods provide fast and rather accurate predictions, because they have fewer hyperparameters than inducing point methods and are locally exact. However, the predictive aggregation of complete independent experts leads to unreliable uncertainty estimates and less accurate predictions in regions between experts. Further, also a rigorous connection to full GP is missing. Beside the mentioned local and global methods, there are also numerical approaches, for instance by exploiting parallelism in specialized hardware (Wang et al. 2019). For a more thorough overview of GP approximations we refer to Liu et al. (2020), Rasmussen and Williams (2006).

Our approach aims to overcome these limitations by introducing a framework based on *J* correlated experts, so that it approximates full GP in two orthogonal directions: sparsity and locality. Thereby, our model is a generalization of the independent PoEs and sparse global GPs by introducing local correlations between experts. These experts correspond to local and sparse GP models represented by a set of *local inducing points*, which are points on the GP summarizing locally the dependencies of the training data. The degree of correlation *C* between the experts can vary between independent up to fully correlated experts in a consistent way, so that our model recovers independent PoEs, sparse global GP and full GP in the limiting cases. Our method exploits the conditional independence between the experts resulting in a sparse and low-rank prior as well as posterior precision (inverse of covariance) matrix, which can be used to efficiently obtain local and correlated predictions from each expert. These correlated predictions are aggregated by the covariance intersection method (Julier and Uhlmann 1997), which is useful for combining consistently several estimates with unknown correlations. The resulting predictive distribution is a smooth weighted average of the predictive distributions of the individual experts. Our algorithm works with a general kernel function and performs well in higher dimensional input spaces. The number of hyperparameters to optimize of our method is the same as for full GP, which are just a few parameters (depending on the kernel). These parameters can be similarly estimated via the log marginal likelihood, which is analytically and efficiently computable for our model. In our inference, also log normal priors can be incorporated leading to maximum-a-posteriori estimates for the hyperparameters.

Compared to the number of *global* inducing point $M_g$, which is usual much smaller than the number of data points *N*, our approach allows a much higher of total *local* inducing points in the order of *N* which helps to cover the space and therefore model more complicated functions. Compared to the independent PoEs, the performance can already significantly improve by modelling just a few of the pairwise correlations between the experts. Our method shares also some similarities with other sparse precision matrix GP approximations. The works Durrande et al. (2019), Grigorievskiy et al. (2017) exploit a band precision matrix together with univariate kernels, whereas Bui and Turner (2014) propose a precision structure according to a tree. The authors Datta et al. (2016), Katzfuss and Guinness (2021) use a more general precision matrix structure, however they need to know the prediction points in advance and are only well suited for low dimensional data (i.e. 1D and 2D), which is usually not useful in the context of machine learning, where the dimension is higher and predictions are needed after training.

In Sect. 2, we briefly review full GP for regression and *sparse and global * as well as *independent and local * approaches for GP approximation. In Sect. 3, we propose our method *Correlated Product of Experts* (CPoEs), where we introduce the graphical model (Sect. 3.1) of our method and explain the local and sparse character of the prior approximation (Sect. 3.2). Further, we discuss how to make inference (Sect. 3.3) and prediction (Sect. 3.4) in our model. In Sect. 3.5, we show that the quality of our approximation consistently improves in terms of Kullback–Leibler-(KL)-divergence (B11) w.r.t. full GP for increasing degree of correlation. Further, we present deterministic and stochastic hyperparameter optimization techniques (Sect. 3.6). In Sect. 4 we compare against state-of-the-art GP approximation methods in a time versus accuracy sense, for synthetic as well as several real-world datasets. Moreover, comparison to non-GP regression methods are provided. We demonstrate superior performance of our proposed method for different (non-trivial) kernels in multiple dimensions. Section 5 concludes the work and presents future research directions.

## GP regression

Suppose we are given a training set $\mathcal {D} = \left\{ y_i, X_i \right\} _{i=1}^N$ of *N* pairs of inputs $X_i\in \mathbb {R}^D$ and noisy scalar outputs $y_i$ generated by adding independent Gaussian noise to a latent function *f*, that is $y_i = f(X_i)+\varepsilon _i$, where $\varepsilon _i\sim \mathcal {N}\left( 0,\sigma _n^2\right)$. We denote $\varvec{y}= [y_1,\ldots ,y_N]^T$ the vector of observations and with $\varvec{X}= [X_1^T,\ldots ,X_N^T]^T \in \mathbb {R}^{N\times D}$. We model *f* with a *Gaussian Process*, i.e. $f\sim$ GP($m, k_{\varvec{\theta }}$) with mean *m*(*X*) and a covariance function (or kernel) $k_{\varvec{\theta }}(X,X')$ for any $X,X'\in \mathbb {R}^D$, where $\varvec{\theta }$ is a set of hyperparemeters. For the sake of simplicity, we assume $m(X)\equiv 0$ and a *squared exponential* (SE) kernel with individual lengthscales for each dimension if not otherwise stated, however, the mean function can be arbitrary and the covariance any positive definite kernel function (see, e.g., Rasmussen and Williams (2006), Chap. 4). For any input matrix $\varvec{A}=[A_1;\ldots ;A_M] \in \mathbb {R}^{M\times D}$ consisting of rows $A_i\in \mathbb {R}^D$, we define the GP output value $\varvec{a}= f\left( \varvec{A}\right) =\left[ f(A_1),\ldots ,f(A_{M})\right] ^T =\left[ a_1,\ldots ,a_{M}\right] ^T \in \mathbb {R}^M$, so that the joint distribution $p\left( \varvec{a}\right) =p\left( a_1,\ldots ,a_{M}\right)$ is Gaussian $\mathcal {N}\left( \varvec{a}\vert \varvec{0}, \varvec{K}_{\varvec{A}\varvec{A}}\right)$ with a kernel matrix $\varvec{K}_{\varvec{A}\varvec{A}}\in \mathbb {R}^{M\times M}$, where the entries $\left[ \varvec{K}_{\varvec{A}\varvec{A}} \right] _{ij} = \varvec{K}_{A_iA_j}$ correspond to the kernel evaluations $k_{\varvec{\theta }}(A_i,A_j)\in \mathbb {R}$. In particular, the joint distribution $p(\varvec{f}, f_*)$ of the training values $\varvec{f}= f\left( \varvec{X}\right) =\left[ f(X_1),\ldots ,f(X_N)\right] ^T$ and a test function value $f_*=f(X_*)$ at test point $X_*\in \mathbb {R}^D$ is Gaussian $\mathcal {N}\left( \varvec{0},\varvec{K}_{ [\varvec{X}; X_*] [\varvec{X}; X_*] } \right)$, where $[\varvec{X}; X_*]$ is the resulting matrix when stacking the matrices above each other. For GP regression, the Gaussian likelihood $p\left( \varvec{y}\vert \varvec{f}\right) = \mathcal {N}\left( \varvec{y}\vert \varvec{f}, \sigma _n^2\mathbb {I}\right)$ can be combined with the joint prior $p(\varvec{f}, f_*)$, so that the predictive posterior distribution can be analytically derived Rasmussen and Williams (2006).

**Fig. 1:**
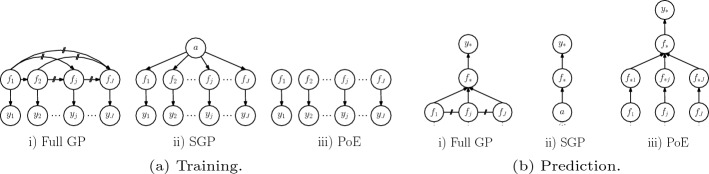
Graphical models of different GP approaches

Alternatively, the posterior distribution over the latent variables given the data can be explicitly formulated as

1 $$\begin{aligned} p\left( \varvec{f}\vert \varvec{y}\right) \propto p\left( \varvec{f},\varvec{y}\right) = \prod _{j=1}^J p\left( \varvec{y}_{j}\vert \varvec{f}_{j}\right) p\left( \varvec{f}_{j}\vert \varvec{f}_{1:j-1}\right) , \end{aligned}$$

where the data is split into *J* mini-batches of size *B*, i.e. $\mathcal {D} = \left\{ \varvec{y}_j, \varvec{X}_j \right\} _{j=1}^J$ with inputs $\varvec{X}_j\in \mathbb {R}^{B\times D}$, outputs $\varvec{y}_j\in \mathbb {R}^B$ and the corresponding latent function values $\varvec{f}_j = f(\varvec{X}_j)\in \mathbb {R}^B$. In (1) we used the notation $\varvec{f}_{k:j}$ indicating $[\varvec{f}_k,\ldots ,\varvec{f}_j]$ and the conditionals $p\left( \varvec{f}_{j}\vert \varvec{f}_{1:j-1}\right)$ can be derived from the joint Gaussian, where we define $p\left( \varvec{f}_{1}\vert \varvec{f}_{1:0}\right) =p(\varvec{f}_{1})$. Given the posterior $p(\varvec{f}\vert \varvec{y})$, the predictive posterior distribution from above is equivalently obtained as $p\left( f_*\vert \varvec{y}\right) = \int p\left( f_*\vert \varvec{f}\right) p\left( \varvec{f}\vert \varvec{y}\right) \mathop {}\!\textrm{d}\varvec{f}$ via Gaussian integration (B7). The corresponding graphical model is depicted in Fig. 1(a)i) and 1(b)i), respectively.

The GP depends via the kernel matrix on the hyperparameters $\varvec{\theta }$, which are typically estimated by maximizing the log marginal likelihood $\log p\left( \varvec{y}\vert \varvec{\theta }\right) = \log \mathcal {N}\left( \varvec{y}\vert \varvec{0}, \varvec{K}_{\varvec{X}\varvec{X}} + \sigma _n^2\mathbb {I}\right) .$ Although GP inference is an elegant probabilistic approach for regression, the computations for inference and parameter optimization require the inversion of the matrix $\varvec{K}_{\varvec{X}\varvec{X}} + \sigma _n^2 \mathbb {I}\in \mathbb {R}^{N\times N}$, which scales as $\mathcal {O}(N^3)$ in time and $\mathcal {O}(N^2)$ for memory which is infeasible for large *N*.

### Global sparse GPs

Sparse GP regression approximations based on *global inducing points* reduce the computational complexity by introducing $M_g \ll N$ inducing points $\varvec{a}\in \mathbb {R}^{M_g}$ that optimally summarize the dependency of the whole training data globally, as illustrated in the graphical model in Fig. 1(a)ii) and is denoted in the following as SGP$(M_g)$. Thereby the inducing *inputs* $\varvec{A}\in \mathbb {R}^{M_g\times D}$ are in the *D*-dimensional input data space and the inducing *outputs* $\varvec{a}= f(\varvec{A})\in \mathbb {R}^{M_g}$ are the corresponding GP-function values.

Similarly to full GP in Eq. (1), the posterior over the inducing points $p(\varvec{a}\vert \varvec{y}) \propto \int p\left( \varvec{a},\varvec{f},\varvec{y}\right) \mathop {}\!\textrm{d}\varvec{f}$ can be derived from the joint distribution

2 $$\begin{aligned} p\left( \varvec{a},\varvec{f},\varvec{y}\right) = \prod _{j=1}^J p\left( \varvec{y}_{j}\vert \varvec{f}_{j}\right) p\left( \varvec{f}_{j}\vert \varvec{a}\right) p(\varvec{a}), \end{aligned}$$

where the usual Gaussian likelihood $p\left( \varvec{y}_{j}\vert \varvec{f}_{j}\right) =\mathcal {N}\left( \varvec{f}_{j},\sigma _n^2\mathbb {I}\right)$ and the Gaussian conditional $p\left( \varvec{f}_{j}\vert \varvec{a}\right)$ are used. Based on the joint distribution in (2), the posterior $p(\varvec{a}\vert \varvec{y})$ can be derived from which prediction can be performed using the predictive conditional $p\left( f_*\vert \varvec{a}\right)$ as more precisely explained in Appendix E.1 and illustrated in Fig. 1(b)ii). Batch inference in these sparse global models can be done in $\mathcal {O}(M_g^2N)$ time and $\mathcal {O}(M_g N)$ space (Quiñonero-Candela and Rasmussen (2005)).

In order to find optimal inducing inputs $\varvec{A}$ and hyperparameters $\varvec{\theta }$, a sparse variation of the log marginal likelihood similar to full GP can be used Bui et al. (2017), Snelson and Ghahramani (2006), Titsias (2009). For larger datasets, stochastic optimization has been applied e.g., Bui et al. (2017), Hensman et al. (2013), Kania et al. (2021), Schürch et al. (2020) to obtain faster and more data efficient optimization procedures. For recent reviews on the subject we refer to Liu et al. (2020), Quiñonero-Candela and Rasmussen (2005), Rasmussen and Williams (2006).

### Local independent GPs

**Fig. 2:**
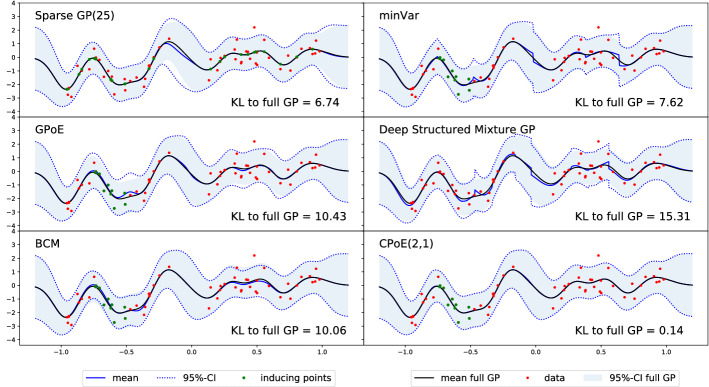
Different GP approximations (with comparable time complexity) indicated with predictive mean (solid blue) and $95\%$-credible interval (dotted blue) compared to full GP (black and shaded blue area). The number in the right bottom corner indicates the KL-divergence (B11) to full GP. In the last plot, our method *Correlated Product of Expert (CPoE)* is presented for a degree of correlation $C=2$ and sparsity $\gamma =1$. We provide a second example in Figure A6 and a discussion about the relation of our method to *deep structured mixture GPs* (Trapp et al. 2020) is given in Sect. A.5

Local approaches constitute an alternative to global sparse inducing point methods, which exploit multiple local GPs combined with averaging techniques to perform predictions. In this work we focus on *Product of Expert (PoE)* Hinton (2002), where individual predictions from *J* experts based on the local data $\varvec{y}_j$ are aggregated to the final predictive distribution

3 $$\begin{aligned} p\left( f_*\vert \varvec{y}\right) = \prod _{j=1}^{J} g_j\left( p\left( f_{*j}\vert \varvec{y}_{j}\right) \right) , \end{aligned}$$

where $g_j$ is a function introduced in order to increase or decrease the importance of the experts and depends on the particular PoE method Hinton (2002), Fleet (2014), Tresp (2000), Liu et al. (2018), Liu et al. (2020). Note, in particular, the *generalized PoE (GPoE)*Fleet (2014), where the weights are set to the difference in entropy of the local prior and posterior. The individual predictions $p\left( f_{*j}\vert \varvec{y}_{j}\right)$ are based on a local GP, for which the implicit joint posterior can be formulated as

4 $$\begin{aligned} p\left( \varvec{f}\vert \varvec{y}\right) \propto p\left( \varvec{f},\varvec{y}\right) = \prod _{j=1}^J p\left( \varvec{y}_{j}\vert \varvec{f}_{j}\right) p\left( \varvec{f}_{j}\right) , \end{aligned}$$

where the corresponding graphical model is depicted in Fig. 1iii) and more details are provided in Appendix E.2. Other important contributions in this field are distributed local GPs Deisenroth and Ng (2015), parallel hierarchical PoEs Buschjäger et al. (2019), and local experts with consistent aggregations Rullière et al. (2018), Nakai-Kasai and Tanaka (2021). A different category of averaging techniques are for instance *mixture of experts* (Masoudnia and Ebrahimpour 2014; Trapp et al. 2020), which basically replace the product in (3) by a sum. A particularly interesting approach is *deep structured mixtures of GPs* (Trapp et al. 2020), which exploits a sum-product network of local and independent GPs. Moreover, simple baseline methods for local methods are the *minimal variance (minVar)* and the *nearest expert (NE)* aggregation, where only the prediction from the expert with minimal variance and nearest expert is used, respectively. Although both methods show often surprisingly good performance, they suffer from the important disadvantage that there are serious discontinuities at the boundaries between the experts (see for instance Fig. 2) and thus often not useful in practice. This is also the main limitation of all local methods based only on the prediction of one single expert (e.g., deep structured mixture GPs (Trapp et al. 2020)), which was the main reason for introducing smooth PoEs with combined experts. We refer to Liu et al. (2020) for a recent overview.

## Correlated product of experts

In this section, we present our GP regression method *Correlated Product of Expert* CPoE$(C,\gamma )$, which is a generalization of the independent PoEs and sparse global GPs. The first generalization is the introduction of correlations between the experts, which can be adjusted by the parameter $1\le C\le J$ and allows to interpolate between local and global models. Secondly, similar to the sparse global approximation, our method allows to sparsify the inducing points by sparsity parameter $0<\gamma \le 1$. We refer to Table 1 in the Appendix for an overview of the used notation.

### Graphical model

Assuming $N=BJ$ data samples which are divided into *J* ordered partitions (or experts) of size *B*, i.e. $\mathcal {D} = \left\{ \varvec{y}_j, \varvec{X}_j \right\} _{j=1}^J$ with inputs $\varvec{X}_j\in \mathbb {R}^{B\times D}$ and outputs $\varvec{y}_j\in \mathbb {R}^B$. We denote $\varvec{f}_j = f(\varvec{X}_j)\in \mathbb {R}^B$ the corresponding latent function values on the GP *f*. We abbreviate $\varvec{y}=\varvec{y}_{1:J}\in \mathbb {R}^{N},\varvec{X}=\varvec{X}_{1:J}\in \mathbb {R}^{N\times D}$ and $\varvec{f}=\varvec{f}_{1:J}\in \mathbb {R}^{N}$.

#### Definition 1

(Local inducing points) We refer to local inducing points $\left\{ \varvec{a}_j, \varvec{A}_j\right\} _{j=1}^J$ with inducing inputs $\varvec{A}_j \in \mathbb {R}^{L\times D}$ and the corresponding inducing outputs $\varvec{a}_j = f(\varvec{A}_j)\in \mathbb {R}^{L}$ of size $L=\lfloor \gamma B \rfloor$ with $0<\gamma \le 1$.

These *L* local inducing points $\left( \varvec{a}_j, \varvec{A}_j\right)$ of expert *j* serve as local summary points for the data $\left( \varvec{y}_j, \varvec{X}_j\right)$, where the sparsity level can be adjusted by $\gamma$. If $\gamma =1$, the inducing inputs $\varvec{A}_j$ correspond exactly to $\varvec{X}_j$ and correspondingly $\varvec{a}_j = \varvec{f}_j$. We abbreviate $\varvec{a}= \varvec{a}_{1:J} \in \mathbb {R}^{M}$, where $M=LJ$, for all local inducing outputs with the corresponding local inducing inputs $\varvec{A}= \varvec{A}_{1:J} \in \mathbb {R}^{M\times D}$. Next, we model connections between the experts by a set of neighbour experts according to the given ordering.

#### Definition 2

(Predecessor and Correlation Index Sets) Let $\phi _{i}(j)\in \{1,\ldots ,j-1\}$ the index of the *i*th predecessor of the *j*th expert. For a given correlation parameter $1\le C\le J$, we introduce the *predecessor set* $\varvec{\pi }_C(j)=\bigcup _{i=1}^{I_j} \phi _{i}(j)$ satisfying

$$\begin{aligned} \varvec{\pi }_C(j) \subset \{1,\ldots ,j-1\} \quad \quad \text {and} \quad \quad \varvec{\pi }_{C+1}(j) = \varvec{\pi }_C(j) ~\cup ~ \phi _{C+1}(j), \end{aligned}$$

such that the size of the set $I_j = \vert \varvec{\pi }_C(j) \vert = \min (j-1,C-1)$.

Further, we define the region of correlation with the *correlation indices* as $\varvec{\psi }_C(j) = \varvec{\pi }_C(j) ~\cup ~j$ if $j > C$ and $\varvec{\psi }_C(j) = \varvec{\psi }_C(C) = \{1,\ldots ,C\}$ otherwise, so that $\vert \varvec{\psi }_C(j)\vert = C$ for all *j*.

The purpose of these predecessor and correlation indices is to model the local correlations among the experts of degree *C*. If for all *j* the indices $\varvec{\pi }_C(j)$ are the $C-1$ previous indices, we say that the predecessors are *consecutive* and *non-consecutive* otherwise. If *C* is clear from the context, $\varvec{\pi }_C(j)$ and $\varvec{\psi }_C(j)$ are abbreviated by $\varvec{\pi }(j)$ and $\varvec{\psi }(j)$, respectively. Details about the specific choices of the ordering, partition, inducing points and predecessor indices are given in Sect. 3.6.1.

**Fig. 3:**
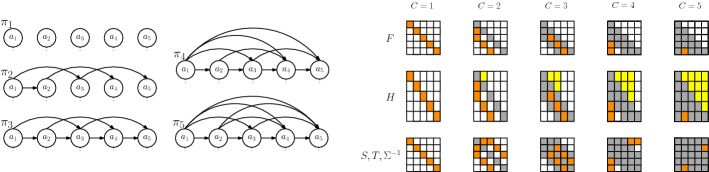
Correlation structure $\varvec{\pi }_C$ between the $J=5$ experts for different degrees of correlation $1\le C\le J$. Left: Graphical model among the local inducing points $\varvec{a}_j$. Right: Structure of sparse transition matrix $\varvec{F}$, projection matrix $\varvec{H}$, prior precision $\varvec{S}$, likelihood precision $\varvec{T}$ and posterior precision $\varvec{\Sigma }^{-1}$. Note that $\varvec{\pi }_C$ does not have to be consecutive, e.g $2 \notin \varvec{\pi }_2(3)$

#### Definition 3

(Graph) We define a directed graph $\mathcal {G}(V,E)$ with nodes $V= \varvec{a}\cup \varvec{f}\cup \varvec{y}$ and directed edges

$$\begin{aligned} E= \{~&\{ (\varvec{a}_{\varvec{\pi }_C^i(j)} ,\varvec{a}_j) \}_{i=1}^ {I_j} ~\cup ~ \{ (\varvec{a}_{\varvec{\psi }_C^i(j)} ,\varvec{f}_j) \}_{i=1}^ {C} ~\cup ~ (\varvec{f}_j,\varvec{y}_j) ~ \}_{j=1}^J, \end{aligned}$$

where $\varvec{\pi }_C^i(j)$ and $\varvec{\psi }_C^i(j)$ denote the *i*th element in the corresponding set.

**Fig. 4:**
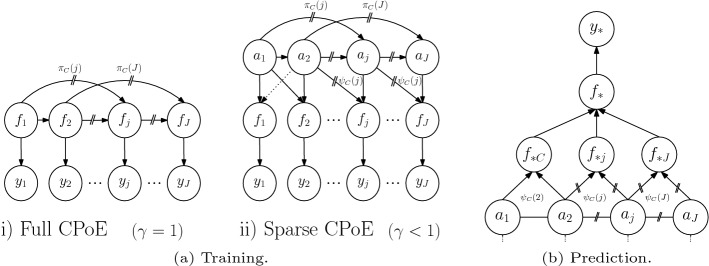
Graphical model for training and prediction of CPoE($C,\gamma$)

The directed graph $\mathcal {G}$ is depicted in Fig. 4aii), where the local inducing points of the *j*th expert are connected with the inducing points of the $I_j$ experts in $\varvec{\pi }_C(j)$. Further, the function values $\varvec{f}_j$ are connected in the region of correlation $\varvec{\psi }_C(j)$ to the local inducing points. The graph $\mathcal {G}=(V,E)$ can be equipped with a probabilistic interpretation, in particular, each node $\varvec{v}\in V$ and each incoming edge $(\varvec{v}_i,\varvec{v})\in E$ for all predecessors $i=1,\ldots ,I$ can be interpreted as a conditional probability density $p\left( \varvec{v}\vert \varvec{v}_1,\ldots ,\varvec{v}_{I}\right)$.

#### Proposition 1

(Graphical Model; Proof 1) We define a graphical model corresponding to the graph $\mathcal {G}(V,E)$ with the conditional probability distributions

5 $$\begin{aligned} p\left( \varvec{y}_j\vert \varvec{f}_{j}\right)&= \mathcal {N}\left( \varvec{y}_j\vert \varvec{f}_{j},\sigma ^2_n\mathbb {I}\right) , \end{aligned}$$

6 $$\begin{aligned} p\left( \varvec{f}_j\vert \varvec{a}_{\varvec{\psi }\left( j\right) }\right)&= \mathcal {N}\left( \varvec{f}_j\vert \varvec{H}_{j}\varvec{a}_{\varvec{\psi }\left( j\right) },\overline{\varvec{V}}_{j}\right) \end{aligned}$$

7 $$\begin{aligned} p\left( \varvec{a}_j\vert \varvec{a}_{\varvec{\pi }\left( j\right) }\right)&= \mathcal {N}\left( \varvec{a}_j\vert \varvec{F}_{j}\varvec{a}_{\varvec{\pi }\left( j\right) },\varvec{Q}_{j}\right) , \end{aligned}$$

where (5) is the usual Gaussian likelihood for GP regression with noise variance $\sigma ^2_n$, (6) the projection conditional and (7) the prior transition. Thereby, the matrices are defined as $\varvec{H}_{j} = \varvec{K}_{\varvec{X}_{j}\varvec{A}_{\varvec{\psi }\left( j\right) }} \varvec{K}_{\varvec{A}_{\varvec{\psi }\left( j\right) }\varvec{A}_{\varvec{\psi }\left( j\right) }}^{-1} \in \mathbb {R}^{B\times LC}$, $\overline{\varvec{V}}_{j} = Diag[ \varvec{K}_{\varvec{X}_j\varvec{X}_j} - \varvec{K}_{\varvec{X}_j\varvec{A}_{\varvec{\psi }\left( j\right) }} \varvec{K}_{\varvec{A}_{\varvec{\psi }\left( j\right) }\varvec{A}_{\varvec{\psi }\left( j\right) }}^{-1} \varvec{K}_{\varvec{A}_{\varvec{\psi }\left( j\right) }\varvec{X}_j} ] \in \mathbb {R}^{B\times B}$, $\varvec{F}_{j} = \varvec{K}_{\varvec{A}_{j}\varvec{A}_{\varvec{\pi }\left( j\right) }} \varvec{K}_{\varvec{A}_{\varvec{\pi }\left( j\right) }\varvec{A}_{\varvec{\pi }\left( j\right) }}^{-1} \in \mathbb {R}^{L\times LI_j}$, and $\varvec{Q}_{j} = \varvec{K}_{\varvec{A}_j\varvec{A}_j} - \varvec{K}_{\varvec{A}_j\varvec{A}_{\varvec{\pi }\left( j\right) }} \varvec{K}_{\varvec{A}_{\varvec{\pi }\left( j\right) }\varvec{A}_{\varvec{\pi }\left( j\right) }}^{-1} \varvec{K}_{\varvec{A}_{\varvec{\pi }\left( j\right) }\varvec{A}_j} \in \mathbb {R}^{L\times L}$ with $\varvec{F}_1 = \varvec{0}$ and $\varvec{Q}_1=\varvec{K}_{\varvec{A}_1\varvec{A}_1}$.

The two conditional distributions (6) and (7) can be derived from the true joint prior distribution $p(\varvec{a},\varvec{f},\varvec{y})$ as shown in Proof 1. Alternatively, a generalization of this model can be obtained when using a modified projection distribution $p\left( \varvec{f}_j\vert \varvec{a}_{\varvec{\psi }\left( j\right) }\right)$, so that for $C\rightarrow J$ and $\gamma <1$ our model recovers a range of well known global sparse GP methods as described in Sect. A.1 and Prop. 5. In any case, these local conditional distributions lead to the following joint distribution.

#### Definition 4

(Joint distribution) For the graphical model corresponding to graph $\mathcal {G}$, the joint distribution over all variables $\varvec{f}, \varvec{a},\varvec{y}$ can be written as

$$\begin{aligned} q_{c,\gamma }(\varvec{f}, \varvec{a},\varvec{y}) = \prod _{j=1}^J p\left( \varvec{y}_{j}\vert \varvec{f}_{j}\right) p\left( \varvec{f}_{j}\vert \varvec{a}_{\varvec{\psi }\left( j\right) } \right) p\left( \varvec{a}_{j}\vert \varvec{a}_{\varvec{\pi }\left( j\right) } \right) . \end{aligned}$$

In the case $\gamma =1$ and thus $\varvec{a}=\varvec{f}$, the joint distribution simplifies (Proof 2) to

$$\begin{aligned} q_{c,1}(\varvec{f},\varvec{y}) = \prod _{j=1}^J p\left( \varvec{y}_{j}\vert \varvec{f}_{j}\right) p\left( \varvec{f}_{j}\vert \varvec{f}_{\varvec{\pi }\left( j\right) } \right) . \end{aligned}$$

We use $q=q_{c,\gamma }$ instead of *p* in order to indicate that it is an approximate distribution. The joint distributions in Def. 4 and the corresponding graphical model in Fig. 4a allow interesting comparisons to other GP models in Fig. 1 and the corresponding formulas (1), (2), (4). Whereas the conditioning set for full GP are all the previous latent values $\varvec{f}_{1:j-1}$, for sparse GPs some global inducing points $\varvec{a}$ and for local independent experts the empty set, we propose to condition on the $C-1$ predecessors $\varvec{f}_{\varvec{\pi }\left( j\right) }$ (or a sparsified version in the general case). From this point of view, we can notice that our probabilistic model is equal to full GP, sparse GP and PoEs under certain circumstances, which are more precisely formulated in Prop. 5.

### Sparse and local prior approximation

The conditional independence assumptions between the experts induced by the predecessor structure $\varvec{\pi }_{C}$ lead to an approximate prior $q_{c,\gamma }(\varvec{a})$ and approximate projection $q_{c,\gamma }(\varvec{f}\vert \varvec{a})$ yielding a sparse and local joint prior $q_{c,\gamma }(\varvec{a},\varvec{f},\varvec{y})$.

#### Proposition 2

**Fig. 5:**
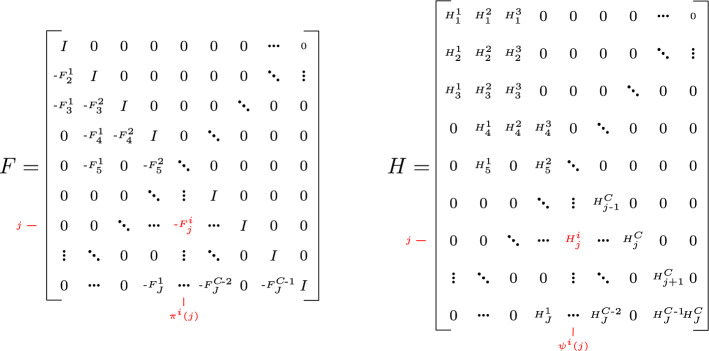
Sparse transition $\varvec{F}\in \mathbb {R}^{M\times M}$ and projection $\varvec{H}\in \mathbb {R}^{N\times M}$ matrices, where $\varvec{F}_j^i \in \mathbb {R}^{L\times L}$ and $\varvec{H}_j^i\in \mathbb {R}^{B\times L}$ are the *i*th part of $\varvec{F}_j \in \mathbb {R}^{L\times L(C-1)}$ and $\varvec{H}_j \in \mathbb {R}^{B\times LC}$, respectively, corresponding to the *i*th entries in $\varvec{\pi }^i(j)$ and $\varvec{\psi }^i(j)$

(Joint prior approximation, Proof 4) The prior over all local inducing points $\varvec{a}$ in our CPoE model is

$$\begin{aligned} q_{c,\gamma }(\varvec{a}) = \prod _{j=1}^J p\left( \varvec{a}_{j}\vert \varvec{a}_{\varvec{\pi }\left( j\right) } \right) = \mathcal {N}\left( \varvec{a}\vert \varvec{0},\varvec{S}_C^{-1}\right) , \end{aligned}$$

with prior precision $\varvec{S}_C = \varvec{S}=\varvec{F}^T \varvec{Q}^{-1} \varvec{F}\in \mathbb {R}^{M\times M}$, where $\varvec{Q}=\text {Diag}\left[ \varvec{Q}_1,\ldots ,\varvec{Q}_J\right] \in \mathbb {R}^{M\times M}$ and $\varvec{F}\in \mathbb {R}^{M\times M}$ is given as the sparse lower triangular matrix in Fig. 5. Moreover, the projection is

$$\begin{aligned} q_{c,\gamma }(\varvec{f}\vert \varvec{a}) = \prod _{j=1}^J p\left( \varvec{f}_{j}\vert \varvec{a}_{\varvec{\psi }\left( j\right) } \right) = \mathcal {N}\left( \varvec{f}\vert \varvec{H}\varvec{a},\overline{\varvec{V}}\right) , \end{aligned}$$

where $\varvec{H}\in \mathbb {R}^{N\times M}$ defined in Fig. 5 and $\overline{\varvec{V}} =\text {Diag}\left[ \overline{\varvec{V}}_1,\ldots ,\overline{\varvec{V}}_J\right] \in \mathbb {R}^{N\times N}$. Together with the exact likelihood $p\left( \varvec{y}\vert \varvec{f}\right) =\prod _{j=1}^J p(\varvec{y}_j\vert \varvec{f}_j )= \mathcal {N}\left( \varvec{y}\vert \varvec{f},\sigma _n^2\mathbb {I}\right)$ determines the joint approximate prior

$$\begin{aligned} q_{c,\gamma }(\varvec{a},\varvec{f},\varvec{y})&= p(\varvec{y}\vert \varvec{f})~ q_{c,\gamma }(\varvec{f}\vert \varvec{a})~ q_{c,\gamma }(\varvec{a}). \end{aligned}$$

Note that the joint prior $q_{c,\gamma }(\varvec{a},\varvec{f},\varvec{y})$ is Gaussian $\mathcal {N}\left( \varvec{0},\varvec{W}\right)$ with dense covariance $\varvec{W}$ and sparse precision $\varvec{Z}= \varvec{W}^{-1}$ as shown in Fig. C7 in the Appendix. If the predecessor set is consecutive, the matrix $\varvec{F}$ is a lower band (block)matrix with bandwidth *C* and in the non-consecutive case each row has exactly *C* non-zero blocks. The sparsity pattern of $\varvec{F}$ is inherited to the prior precision $\varvec{S}= \varvec{F}^T \varvec{Q}^{-1} \varvec{F}$, which is also a sparse matrix (see Fig. 3). For the consecutive case, $\varvec{S}$ is a block-band matrix with bandwidth $2C-1$. Note that, the inverse $\varvec{S}^{-1}$ is dense. The likelihood matrix $\varvec{H}$ is exact in the corner up to indices *C* which ensures that our model recovers sparse global GP in the limiting case $C=J$. The quality of the approximation of our CPoE$(C, \gamma )$ model is discussed in Sect. 3.5, where we show that $q_{c,\gamma }(\varvec{a},\varvec{f},\varvec{y})$ converges to the true prior $p(\varvec{a},\varvec{f},\varvec{y})$ for $C \rightarrow J$.

### Inference

For our model it is possible to infer analytically the posterior $q_{c,\gamma }(\varvec{a}\vert \varvec{y})$ and the marginal likelihood $q_{c,\gamma }(\varvec{y})$ used later for prediction and for hyperparameter estimation, respectively.

#### Proposition 3

(Posterior approximation; Proof 12) From the joint distribution, the latent function values $\varvec{f}$ can be integrated out yielding

$$\begin{aligned} q_{c,\gamma }( \varvec{a},\varvec{y})&= \int q_{c,\gamma }(\varvec{f}, \varvec{a},\varvec{y})\mathop {}\!\textrm{d}\varvec{f}=q_{c,\gamma }(\varvec{y}\vert \varvec{a}) q_{c,\gamma }(\varvec{a}) = \mathcal {N}\left( \varvec{y}\vert \varvec{H}\varvec{a},\varvec{V}\right) \mathcal {N}\left( \varvec{a}\vert \varvec{0},\varvec{S}^{-1}\right) \end{aligned}$$

with $\varvec{V}=\overline{\varvec{V}} + \sigma _n^2\mathbb {I}\in \mathbb {R}^{N\times N}.$ The posterior can be analytically computed by

$$\begin{aligned} q_{c,\gamma }( \varvec{a}\vert \varvec{y})&= \frac{q_{c,\gamma }( \varvec{a},\varvec{y})}{q_{c,\gamma }( \varvec{y})} \propto q_{c,\gamma }( \varvec{a},\varvec{y}) = \mathcal {N}\left( \varvec{a}\vert \varvec{\mu },\varvec{\Sigma }\right) = \mathcal {N}^{-1}(\varvec{a}\vert \varvec{\eta }, \varvec{\Lambda }), \end{aligned}$$

with $$\varvec{\Sigma }^{-1} = \varvec{\Lambda }= \varvec{T}+ \varvec{S}\in \mathbb {R}^{M\times M}$$, $$\varvec{\mu }= \varvec{\Sigma }\varvec{\eta }\in \mathbb {R}^{M}$$, $$\varvec{\eta }= \varvec{H}^T\varvec{V}^{-1}\varvec{y}\in \mathbb {R}^{M}$$ and $$\varvec{T}= \varvec{H}^T\varvec{V}^{-1}\varvec{H}\in \mathbb {R}^{M\times M}$$.

**Fig. 6:**
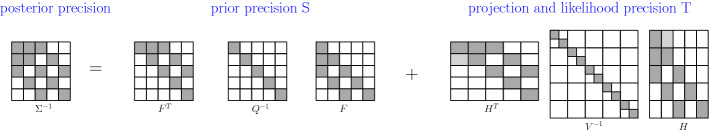
Sparse posterior precision approximation

The posterior precision matrix $\varvec{\Sigma }^{-1}= \varvec{T}+ \varvec{S}$ inherits the sparsity pattern of the prior, since the addition of the projection precision $\varvec{T}=\varvec{H}^T\varvec{V}^{-1}\varvec{H}$ has the same sparsity structure, as depicted in Figs. 3 and 6. On the other hand, the posterior covariance $\varvec{\Sigma }$ is dense, therefore it will be never explicitly fully computed. Instead, the sparse linear system of equations $\varvec{\Sigma }^{-1}\varvec{\mu }= \varvec{\eta }$ can be efficiently solved for $\varvec{\mu }= \varvec{\Sigma }\varvec{\eta }$. Further, in our CPoE model, the marginal likelihood $q_{c,\gamma }(\varvec{y}\vert \varvec{\theta })$ can be analytically computed by $\int q_{c,\gamma }(\varvec{y},\varvec{a}) \mathop {}\!\textrm{d}\varvec{a}= \mathcal {N}\left( \varvec{0},\varvec{P}\right)$ (see Proof 9) with the (dense) matrix $\varvec{P}=\varvec{H}\varvec{S}^{-1}\varvec{H}^T +\varvec{V}\in \mathbb {R}^{N\times N}$, which is used in Sect. 3.6.2 for hyperparameter optimization. The posterior approximation $q_{c,\gamma }(\varvec{a}\vert \varvec{y})$ as well as the approximate marginal likelihood $q_{c,\gamma }(\varvec{y})$ converge to the true distributions $p\left( \varvec{a}\vert \varvec{y}\right)$ and $p\left( \varvec{y}\right)$, respectively, for $C\rightarrow J$. In particular, they correspond exactly to the posterior and marginal likelihood of full GP and sparse global GP with $\lfloor \gamma N\rfloor$ inducing points for $C=J, \gamma =1$ and $C=J, \gamma <1$, respectively.

### Prediction

The final predictive posterior distribution is obtained by an adaptation of the PoE aggregation in (3). The main idea is to consistently aggregate weighted local predictions form the experts, such that the correlations between them are taken into account resulting in a smooth and continuous predictive distribution.

#### Proposition 4

(Prediction aggregation; Proof 17) Similarly to the PoE aggregation (3), we define the final predictive posterior distribution for a query point $\varvec{x}_*\in \mathbb {R}^D$ as

8 $$\begin{aligned} q_{c,\gamma }(f_*\vert \varvec{y}) = \prod _{j=C}^{J} q_{c,\gamma }(f_{*j}\vert \varvec{y}) ^{\beta _{*j}}, \end{aligned}$$

involving the local predictions $q_{c,\gamma }( f_{*j}\vert \varvec{y})=\mathcal {N}\left( m_{*j},v_{*j}\right)$ and weights $\beta _{*j}\in \mathbb {R}$ defined in Prop. 8 and Def. 5, respectively. Moreover, the distribution $q_{c,\gamma }(f_*\vert \varvec{y})=\mathcal {N}\left( m_*,v_*\right)$ with $m_* = v_{*} \sum _{j=C}^{J} \beta _{*j} \frac{m_{*j}}{v_{*j}}$ and $\frac{1}{v_*} = \sum _{j=C}^{J} \frac{\beta _{*j}}{v_{*j}}$ is analytically available. The final noisy prediction is $p\left( y_*\vert \varvec{y}\right) =\mathcal {N}\left( m_*,v_* + \sigma _n^2\right)$.

The graphical model corresponding to this prediction procedure is depicted in Fig. 4b and A3 in the Appendix. Further, the local predictions $q_{c,\gamma }(f_{*j}\vert \varvec{y})$ in Equation (8) are based on the region $\varvec{\psi }\left( j\right)$, where the correlations are modelled and can be computed as $q_{c,\gamma }(f_{*j}\vert \varvec{y}) = \int p\left( f_{*j}\vert \varvec{a}_{\varvec{\psi }\left( j\right) }\right) q_{c,\gamma }(\varvec{a}_{\varvec{\psi }\left( j\right) }\vert \varvec{y}) \mathop {}\!\textrm{d}\varvec{a}_{\varvec{\psi }\left( j\right) }$ involving the local posteriors $q_{c,\gamma }(\varvec{a}_{\varvec{\psi }\left( j\right) }\vert \varvec{y}) = \mathcal {N}\left( \varvec{\mu }_{\varvec{\psi }\left( j\right) }, \varvec{\Sigma }_{\varvec{\psi }\left( j\right) }\right)$ and the predictive conditional $p\left( f_{*j}\vert \varvec{a}_{\varvec{\psi }\left( j\right) }\right)$, as thoroughly shown in Proposition 8 in the Appendix. Thereby, the local posteriors with mean $\varvec{\mu }_{\varvec{\psi }\left( j\right) }$ and covariance entries $\varvec{\Sigma }_{\varvec{\psi }\left( j\right) }$ could be obtained from the corresponding entries $\varvec{\psi }\left( j\right)$ of $\varvec{\mu }$ and $\varvec{\Sigma }$. However, computing explicitly some entries in the dense covariance $\varvec{\Sigma }$ based on the sparse precision $\varvec{\Sigma }^{-1}$ is not straightforward since in the inverse the blocks are no longer independent. However, we can exploit the particular sparsity and block-structure of our precision matrix and obtain an efficient implementation of this part, which is key to achieve a competitive performance of our algorithm. More details are given in the Appendix in Sect. A.2.

#### Definition 5

(Aggregation weights) The input depending weights $\beta _{*j}=\beta _j(X_*)$ at query point $X_*$ model the impact of expert *j*. In particular, the unnormalized weights

$$\begin{aligned} \begin{aligned} \bar{\beta }_{*j}&= H[p\left( f_{*}\right) ] - H[p\left( f_{*j}\vert \varvec{y}\right) ] = \frac{1}{2} \log \left( \frac{v_{*0}}{v_{*j}} \right) , \end{aligned} \end{aligned}$$

are set to the difference in entropy *H* (B10) before and after seeing the data similarly proposed by Fleet (2014). Thereby, the predictive prior is $p\left( f_{*}\right) =\mathcal {N}\left( 0,v_{*0}\right)$ with $v_{*0} = \varvec{k}_{X_*X_*}$ and the predictive posterior defined in Prop. 8. The normalized weights are then obtained by $\beta _{*j} = b^{-1}\bar{\beta }_{*j}^Z$ where $b = \sum _{j=C}^J \bar{\beta }_{*j}^Z$ and $Z=\log (N) C$.

These weights bring the flexibility of increasing or reducing the importance of the experts based on the predictive uncertainty. However, independent of the particular weights, our aggregation of the predictions is consistent since it is based on the *covariance intersection* method (Julier and Uhlmann 1997), which is useful for combining several estimates of random variables with known mean and variance but unknown correlation between them.

### Properties

#### Proposition 5

(Equality; Proof 3) Our model correlated Product of Experts CPoE$(C,\gamma )$ is equal to full GP for $C=J$ and $\gamma =1$. For $\gamma <1$, our model correspond to sparse global GP with $M_g= \lfloor \gamma N \rfloor$ inducing points. Further, with $C=1$ and $\gamma =1$, our model is equivalent to independent PoEs. That is, we have

$$\begin{aligned} \text {CPoE}(J,1 ) = \text {GP}; \quad \text {CPoE}(J,\gamma ) = \text {SGP}(\lfloor \gamma N\rfloor ); \quad \text {CPoE}(1,1) = \text {GPoE}^*, \end{aligned}$$

where SGP refers to the FITC model Snelson and Ghahramani (2006) and GPoE$^*$ correspond to GPoE Fleet (2014) with slightly different weights ($Z=1$) in the prediction.

In Sect. A.1 in the Appendix we present a generalization of our model, so that CPoE($J,\gamma$) correspond to a range of other well known versions of sparse global GP by changing the projection distribution and adding a correction term in the log marginal likelihood similarly discussed in Schürch et al. (2020) for the global case. For instance, we can extend our model analogously to the variational version of Titsias (2009).

For correlations between the limiting cases $C=1$ and $C=J$, we investigate the difference in KL of the true GP model with CPoE$(C,\gamma )$ and CPoE$(C_2,\gamma )$ for $1\le C\le C_2\le J$. For that reason, we define the difference in KL between the true distribution of $\varvec{x}$ and two different approximate distributions, i.e.

$$\begin{aligned} \mathbb {D}_{(C,C_2)}[\varvec{x}] = KL[p\left( \varvec{x}\right) \mid \mid q_{c,\gamma }(\varvec{x})] - KL[p\left( \varvec{x}\right) \mid \mid q_{{c_2,\gamma }}(\varvec{x})]. \end{aligned}$$

Similarly, the difference in KL for a conditional distribution is defined in Eq. (B15). Using these definitions, we show that the approximation quality of the prior $q_{c,\gamma }(\varvec{a})$ and projection approximation $q_{c,\gamma }(\varvec{f}\vert \varvec{a})$ monotonically improves for $C\rightarrow J$, so that the KL between the true joint distribution $p(\varvec{a}, \varvec{f}, \varvec{y})$ and our approximate joint distribution $q_{c,\gamma }(\varvec{a}, \varvec{f}, \varvec{y})$ is decreasing for $C\rightarrow J$.

#### Proposition 6

(Decreasing KL; Proof 6) For any predecessor structure $\varvec{\pi }_C$ and any $0<\gamma \le 1$ and $1\le C\le C_2 \le J$, the difference in KL of the marginal prior, projection and data likelihood are non negative, i.e.

$$\begin{aligned} \mathbb {D}_{(C,C_2)}[\varvec{a}] \ge ~ 0, \quad \quad \mathbb {D}_{(C,C_2)}[\varvec{f}\vert \varvec{a}] \ge ~ 0, \quad \quad \mathbb {D}_{(C,C_2)}[\varvec{y}\vert \varvec{f}] = ~ 0, \end{aligned}$$

so that the joint difference in KL is also non-negative

$$\begin{aligned}&~\mathbb {D}_{(C,C_2)}[\varvec{a}, \varvec{f}, \varvec{y}] = \mathbb {D}_{(C,C_2)}[\varvec{a}] + \mathbb {D}_{(C,C_2)}[\varvec{f}\vert \varvec{a}] + \mathbb {D}_{(C,C_2)}[\varvec{y}\vert \varvec{f}] ~\ge ~ 0. \end{aligned}$$

Moreover, we can quantify the approximation quality, in particular $\mathbb {D}_{(C,C_2)}[\varvec{a}] =\frac{1}{2} \log \frac{\vert \varvec{Q}_{C}\vert }{\vert \varvec{Q}_{C_2}\vert }$ and $\mathbb {D}_{(C,C_2)}[\varvec{f}\vert \varvec{a}] = \frac{1}{2} \log \frac{\vert \bar{\varvec{V}}_{C}\vert }{\vert \bar{\varvec{V}}_{C_2}\vert }.$

**Fig. 7:**
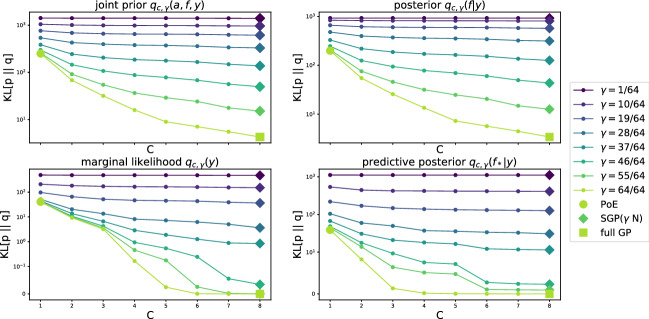
Decreasing KL$[p \vert \vert q]$ between true distribution *p* of full GP and approximate distribution $q=q_{c,\gamma }$ of CPoE for increasing values of *C* and $\gamma$ for the joint prior, posterior, marginal likelihood and predictive posterior for synthetic GP data ($N=1024, D=2$, SE kernel)

The last statement demonstrates that our CPoE model is a sound GP prior precision approximation, which converges monotonically to the true prior for $C\rightarrow J$. The decreasing KL of the joint prior is depicted in Fig. 7 together with the decreasing KL of the posterior, marginal likelihood and predictive posterior. More details and proofs are given in Appendix C.

### Computational details

#### Graph

**Fig. 8:**
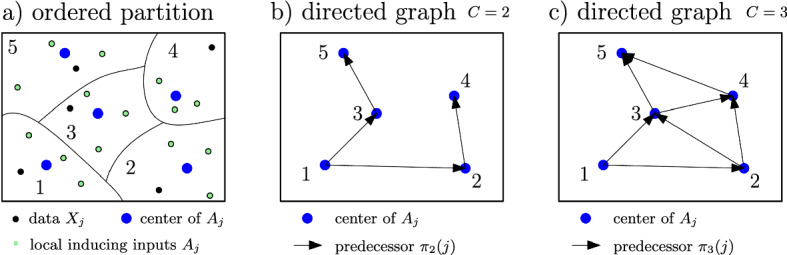
Toy example for partition, local inducing points, predecessors and directed graph illustrated for $D=2$ with $J=5$ experts/partitions each with $B=4$ samples, $\gamma =0.75$ and thus $L=3$ local inducing points. In a) the ordered partition with the data (black), local inducing points (green) and their mean (blue) are depicted. In b) and c) the directed graph for $C=2$ and $C=3$ are shown with corresponding predecessors $\varvec{\pi }_2(1)=\{\}$, $\varvec{\pi }_2(2)=\{1\}$, $\varvec{\pi }_2(3)=\{1\}$, $\varvec{\pi }_2(4)=\{2\}$, $\varvec{\pi }_2(5)=\{3\}$ and $\varvec{\pi }_3(1)=\{\}$, $\varvec{\pi }_3(2)=\{1\}$, $\varvec{\pi }_3(3)=\{1,2\}$, $\varvec{\pi }_3(4)=\{2,3\}$, $\varvec{\pi }_3(5)=\{3,4\}$, respectively. In the previous example, $\varvec{\pi }_3$ is consecutive and $\varvec{\pi }_2$ is non-consecutive

The graphical model in Sect. 3.1 is generically defined and several choices are left for completely specifying the graph $\mathcal {G}(V,E)$ for a particular dataset: the partition method, the ordering of the partition, the selection of the predecessors and the local inducing points. We tried to make these choices as simple and straightforward as possible with focus on computational efficiency, however, there might be more sophisticated heuristics. Concretely, we use KD-trees Maneewongvatana and Mount (2001) for partitioning the data $\mathcal {D}$ into *J* regions and the ordering starts with a random partition which is then greedily extended by the closest partition in euclidean distance (represented by the mean of the inducing points). The $L\le B$ inducing inputs $\varvec{A}_j\in \mathbb {R}^{L\times D}$ of the *j*th partition (or expert) can be in principle arbitrary, however, in this work they are chosen as a random subset of the data inputs $\varvec{X}_j\in \mathbb {R}^{B\times D}$ of the *j*th expert for the sake of simplicity. For the predecessors (block-)indices $\varvec{\pi }_C$, the $C-1$ closest partitions among the previous (according to the ordering) predecessors in euclidean distance are greedily selected. These concepts are illustrated for a toy example in Fig. 8.

#### Hyperparameter estimation

In Sect. 3, we introduced CPoE for fixed hyperparameters $\varvec{\theta }$ where implicitly all distributions are conditioned on $\varvec{\theta }$, however, we omitted the dependencies on $\varvec{\theta }$ in the most cases for the sake of brevity. Similar to full GP, sparse GP or PoEs, the *log marginal likelihood (LML)* can be used as an objective function for optimizing the few hyperparameters $\varvec{\theta }$. The log of the marginal likelihood of our model formulated in Sect. 3.3 is $\mathcal {L}(\varvec{\theta }) = \log q\left( \varvec{y}\vert \varvec{\theta }\right) = \log \mathcal {N}\left( \varvec{0},\varvec{P}\right)$ with $\varvec{P}=\varvec{H}\varvec{S}^{-1}\varvec{H}^T +\varvec{V}$ which can be efficiently computed as detailed in Sect. A.3 and can be used for *deterministic optimization* with full batch $\varvec{y}$ for moderate sample size *N*. However, in order to scale this parameter optimization part to larger number of samples *N* in a competitive time, *stochastic optimization* techniques exploiting subsets of data have to be developed similarly done for the global sparse GP model (SVI Hensman et al. (2013); REC Schürch et al. (2020); IF Kania et al. (2021)). We adapt the hybrid approach IF of Kania et al. (2021) where we can also exploit an independent factorization of the log marginal likelihood which decomposes into a sum of *J* terms, so that it can be used for stochastic optimization. This constitutes a very fast and accurate alternative for our method as shown in the Appendix A.3 and will also be exploited in Sect. 4 for large data sets. Alternatively to the log marginal likelihood (LML) maximization as presented above, the *maximum a posteriori* (MAP) estimator for $\varvec{\theta }$ can be used. This means, that some suitable prior on the hyperparameters are introduced, as explained in Sect. A.3.3 and an example is presented in Sect. 4.4.

#### Complexity

The time complexity for computing the posterior and the marginal likelihood in our algorithm is dominated by *J* operations which are cubic in *LC* (inversion, matrix-matrix multiplication, determinants). This leads to $\mathcal {O}(NB^2\alpha ^3)$ and $\mathcal {O}(NB\alpha ^2)$ for time and space complexity, respectively, where we define the approximation quality parameter $\alpha =C\gamma$. Similarly, for $N_t$ testing points the time and space complexities are $\mathcal {O}(N B\alpha ^2 N_t)$ and $\mathcal {O}(N \alpha N_t)$ (an approach to remove the dependency of *N* is outlined in A.4). In Table 1, the asymptotic complexities of our model together with other GP algorithms are indicated. It is interesting that for $\alpha = 1$, our algorithm has the same asymptotic complexity for training as sparse global GP with $M_g=B$ global inducing points but we can have $M = LJ=\gamma BJ=\gamma N$ total local inducing points! Thus, our approach allows much more total local inducing points *M* in the order of *N* (e.g., $M=0.5N$ with $C=2$) whereas for sparse global GP usually $M_{g} \ll N$. This has the consequence that the local inducing points can cover the input space much better and therefore represent much more complicated functions. As a consequence, there is also no need to optimize the local inducing points resulting in much fewer parameters to optimize. Consider the following example with $N=10'000$ in $D=10$ dimensions. Suppose a sparse global GP model with $M_g=500$ global inducing points. A CPoE model with the same asymptotic complexity has a batch size $B=M_g=500$ and $\alpha =1$. Therefore, we have $J=\frac{N}{B}=20$ experts and we choose $C=2$ and $\gamma =\frac{1}{2}$ such that we obtain $L=\gamma B=250$ local inducing points per experts and $M = \gamma N = 5'000$ total local inducing points! Further, the number of hyperparameters to optimize with a SE kernel is for global sparse GP $M_g D+\vert \varvec{\theta }\vert = 5012$, whereas for CPoE there are only $\vert \varvec{\theta }\vert = 12$. For an extended version of this section consider A.4 in the Appendix.

**Table 1:** Complexity for training, pointwise predictions for $N_t$ points and number of optimization parameters for different GP algorithms

	Full GP	Sparse GP	PoE	CPoE
Time	$\mathcal {O}(N^3)$	$\mathcal {O}(NM_g^2)$	$\mathcal {O}(NB^2)$	$\mathcal {O}(NB^2\alpha ^3)$
Space	$\mathcal {O}(N^2)$	$\mathcal {O}(NM_g)$	$\mathcal {O}(NB)$	$\mathcal {O}(NB\alpha ^2)$
Time$_{t}$	$\mathcal {O}(N^2 N_t)$	$\mathcal {O}(M_g^2 N_t)$	$\mathcal {O}(NBN_t)$	$\mathcal {O}(NBN_t \alpha ^2)$
Space$_{t}$	$\mathcal {O}(N N_t)$	$\mathcal {O}(M_g N_t)$	$\mathcal {O}(NN_t)$	$\mathcal {O}(NN_t \alpha )$
$\#$pars	$\vert \varvec{\theta }\vert$	$MD+ \vert \varvec{\theta }\vert$	$\vert \varvec{\theta }\vert$	$\vert \varvec{\theta }\vert$

## Comparison

In this section, we compare the performance with competitor methods for GP approximations using *synthetic* and several *real world* datasets as summarized in Table 3. Moreover, we provide a comparison to non-GP regression methods as well as an application about probabilistic time series prediction both exploiting non-trivial kernels. More details about the experiments and implementations are provided in Sects. A.6, A.7 and F in the Appendix.

### Synthetic data

**Fig. 9:**
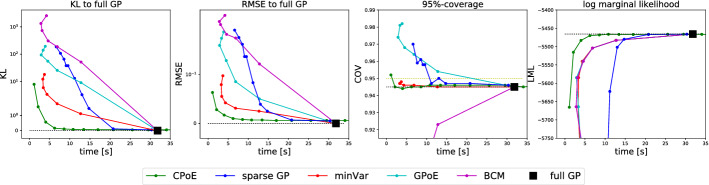
Average accuracy versus time performance of different GP algorithms

First, we examine the accuracy vs. time performance of different GP algorithms for fixed hyperparameters in a simulation study with *synthetic GP data*. We generated $N=8192$ data samples in $D=2$ with 5 repetitions from the sum of two SE kernels with a shorter and longer lengthscale such that both global and local patterns are present in the data (compare Fig. A5). In Fig. 9 the mean results are shown for the KL and RMSE to full GP, the 95%-coverage and the log marginal likelihood against time in seconds. The results for sparse GP with increasing number of global inducing points *M* are shown in blue, the results for minVar, GPoE and BCM for increasing number of experts *J* are depicted in red, cyan and magenta, respectively. For CPoE, the results for increasing correlations *C* are shown in green. We observe superior performance of our method compared to competitors in terms of accuracy compared to full GP versus time. Moreover, one can observe that the confidence information of our model are reliable already for small approximation orders since it is based on the consistent covariance intersection method. A precise description of the experiment is provided in Sect. A.7.1 in the Appendix.

### **Real world data**

Second, we benchmark our method with 10 real world datasets as summarized in Table 3 and more details are given in Sect. A.7.2 in the Appendix (e.g., how to access and pre-process the data). For the 5 smaller datasets in the first block we use deterministic parameter optimization for which the average results over 10 training/testing splits are depicted in Table 2. In particular, the KL to full GP (left) and time (right) for different GP methods are shown. Similarly, the average accuracy and times for the 4 larger datasets in the second block where stochastic parameter optimization is exploited can be found in Table A4 in the Appendix.

In general, the local methods perform better than the global sparse method. Further, the performance of our correlated PoEs is superior to the one of independent PoEs for all datasets. In particular, the KL to full GP can be continuously improved for increasing degree of correlation, i.e. larger *C* values. The time for CPoE(1) is comparable with the independent PoEs and for increasing *C*, our approximation has a moderate increase in time with a significant decrease in KL. For more details about the experiments consider Sect. A.7.2 in the Appendix and more results including standard deviations are provided in Appendix F.

**Table 2:** Average KL to full GP (left) and time (right) for different GP methods and 5 datasets with 10 repetitions. More results are provided in Appendix F

	KL					Time				
	Concrete	mg	Space	Abalone	Kin	Concrete	mg	Space	Abalone	Kin
fullGP	0.0	0.0	0.0	0.0	0.0	7.3	25.5	114.8	237.9	161.5
SGP(100)	352.9	9.9	108.1	15.6	603.7	36.4	14.4	46.6	58.9	42.2
minVar	122.2	19.4	63.6	25.1	211.0	1.5	2.0	7.2	6.4	9.3
GPoE	174.4	54.2	98.0	50.3	342.3	1.4	1.9	7.2	6.3	9.4
GRBCM	224.6	69.1	105.6	36.4	129.8	1.7	2.3	6.5	7.6	11.9
CPoE(1)	111.1	12.2	63.0	16.8	152.4	1.5	2.1	7.8	6.4	9.2
CPoE(2)	89.6	8.4	36.5	8.1	79.9	2.1	2.8	10.6	7.5	12.9
CPoE(3)	82.2	7.8	36.3	6.2	46.9	2.5	3.1	12.9	9.3	19.8
CPoE(4)	**79.5**	**7.6**	**36.0**	**4.7**	**32.8**	2.8	3.3	14.9	10.4	27.8

**Table 3:**
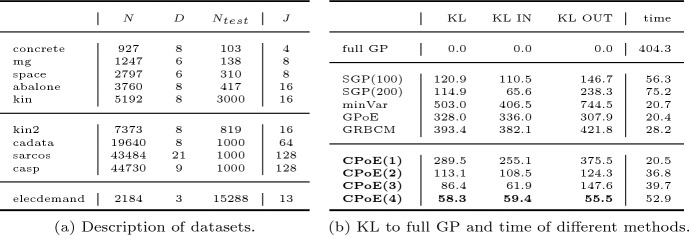
Summary of used datasets and results for the *elecdemand* time series

### Comparison to non-GP methods

Third, we compare our probabilistic regression method CPoE to other popular non-GP regression methods, in particular, dense neural networks (MLPs), eXtreme Gradient Boosting (XGboost) and linear regression^1^. We use three different architectures for the neural networks, that is, MLP(100, 100), MLP(500, 500), *MLP*(100, 100, 100), where the numbers in the parentheses correspond to the number of hidden nodes per hidden layer. Moreover, we used ADAM optimizer with learning rate 0.01. For XGboost$(max\_depth, n\_estimators, learning\_rate)$, we use XGboost(3, 100, 0.1). All these hyperparameters are chosen in primary experiments so that those methods obtain advantageous test performance. For our CPoE method, we use a SE kernel as in the previous sections, and in addition, we run the algorithm with a more flexible kernel, namely

9 $$\begin{aligned} k_{\theta }(\varvec{x}_1,\varvec{x}_2) = k_{SM_1}(\varvec{x}_1,\varvec{x}_2)+ k_{SM_2}(\varvec{x}_1,\varvec{x}_2)+ k_{MLP}(\varvec{x}_1,\varvec{x}_2)+ k_{LIN}(\varvec{x}_1,\varvec{x}_2), \end{aligned}$$

where $k_{SM_i}$ is a spectral-mixture kernel (Wilson and Adams 2013), $k_{MLP}$ an (infinite) wide 1-hidden-layer neural network kernel (Neal 1995) and $k_{LIN}$ a linear kernel. We run full GP for smaller datasets as comparison. The average *RMSE*, *ABSE* and *time* results are provided in Tables 4, F22 and F23, respectively. For instance in Table 4, we can observed that the GP approximation methods using either a SE kernel or a more flexible kernel achieve competitive performance.

Finally, we would like to emphasize that our probabilistic CPoE model provides a predictive *distribution*, that is, it models the predictive uncertainty and can thus provide reliable credible-intervals. Computing also the predictive variances is a harder task than only computing the predictive means, as the most other regression algorithms do. Therefore, the slightly higher computational times (Table F23) for similar accuracy (Tables 4 and F22) are very reasonable in our opinion. More detailed results are given in Tables F15–F21 and on github.^2^

**Table 4:** Average *RMSE* for our CPoE methods compared to non-GP regression methods

	Concrete	mg	Space	Abalone	Kin	Cadata	Sarcos	Casp
fullGP-SE	0.311	0.511	0.471	0.635	0.267			
fullGP-FLEX	0.254	0.509	0.455	0.638	0.28			
CPoE(1)-SE	0.333	**0.508**	0.506	0.637	0.31	0.476	0.099	0.597
CPoE(2)-SE	0.326	0.512	0.49	0.634	0.292	0.47	0.1	0.59
CPoE(3)-SE	0.323	0.513	0.489	**0.634**	**0.28**	0.47	0.099	0.59
CPoE(1)-FLEX	0.266	0.511	0.631	0.687	0.334	0.456	0.094	0.525
CPoE(2)-FLEX	0.259	0.515	0.446	0.669	0.315	0.423	0.094	0.522
CPoE(3)-FLEX	**0.255**	0.516	**0.444**	0.659	0.303	**0.42**	**0.092**	**0.522**
MLP(100-100)	0.289	0.525	0.482	0.652	0.287	0.456	0.117	0.591
MLP(500-500)	0.292	0.522	0.475	0.761	0.284	0.485	0.097	0.577
MLP(100-100-100)	0.285	0.531	0.476	0.762	0.299	0.485	0.106	0.585
XGboost	0.323	0.545	0.543	0.65	0.667	0.474	0.251	0.767
LinReg	0.626	0.633	0.645	0.66	0.765	0.605	0.27	0.854

The methods ending with SE were run with a squared-exponential and a flexible kernel (9), respectively. Best method (beside GP full) is indicated in bold

### Time series application

**Fig. 10:**
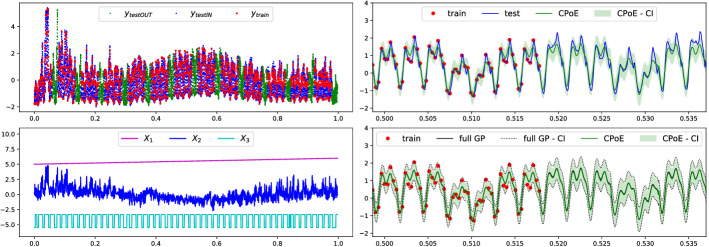
Time series data with covariates and prior on hyperparameters

In this section, our method is applied on time series data with covariates using a non-stationary kernel together with priors on the hyperparameters as discussed in Sect. A.3.3 by using MAP estimation. A recent work Corani et al. (2021) demonstrates that GPs constitute a competitive method for modelling time series using a sum of kernels including priors on the hyperparameters, which are previously learnt from a large set of different time series. We adapt their idea by using the same priors and a slightly modified kernel. In particular, for two data points $\varvec{x}_1=[t_1, x_{1,2}, \ldots ,x_{1,D}]$ and $\varvec{x}_2=[t_2, x_{2,2}, \ldots ,x_{2,D}]$, we model the kernel as the sum of 4 components

$$\begin{aligned} k_{\varvec{\theta }}(\varvec{x}_1,\varvec{x}_2)&= k_{P_1}(t_1,t_2) + k_{P_2}(t_1,t_2) + k_{SM}(t_1,t_2) + k_{SE}(\varvec{x}_1,\varvec{x}_2), \end{aligned}$$

where $k_{P_1}$ and $k_{P_1}$ are standard periodic kernels with period $p_1$ and $p_2$, respectively, $k_{SM}$ a spectral-mixture kernel and $k_{SE}$ a squared-exponential kernel. Note that, the former 3 kernels only depend on the first variable corresponding to time, whereas the SE-kernel depends on all variables, thus models the influence of the additional variables. With our CPoE model it is straightforward to handle time series with covariates, as opposed to other time series methods (Benavoli and Corani (2021), Corani et al. (2021), Sarkka et al. (2013), Hyndman and Athanasopoulos (2018)). We demonstrate the MAP estimation for $\varvec{\theta }$ on the *elecdemand* time series (Hyndman (2020), Table 3), which contains the electricity demand as response *y* together with the time as the first variable $x_1$, the the corresponding temperature as $x_2$ and the variable whether it is a working day as $x_3$ which is depicted in the plots in Fig. 10 on the left, where we shifted the first and third variable in the second plot for the sake of clarity. Similarly as in the previous section, we run full GP, SGP, PoEs and CPoE and optimized the hyperparameter deterministically using the MAP as objective function taking into account the priors. The results are provided in Table 3 and in Fig. 10 on the right, which again show very competitive performance also for a general kernel with priors on the hyperparameters. More details about the experiment is given in Sect. A.7.3 in the Appendix.

## Conclusion

In this paper, we introduce a novel GP approximation algorithm CPoE, where the degree of approximation can be adjusted by a locality and a sparsity parameter, so that the proposed method recovers independent PoEs, sparse global GP and full GP. Thereby, our method consistently approximates full GP, in particular, we proved that increasing the correlations between the experts decreases monotonically the KL of the joint prior of full GP to them of our model. The presented algorithm has only a few hyperparameters, which allows an efficient deterministic and stochastic optimization. Further, our presented algorithm works with a general kernel, with several variables and also priors on the hyperparameters can be included. Moreover, the time and space complexity is linear in the number of experts and number of data samples, which makes it highly scalable. This is demonstrated with efficient implementations, so that a dataset with several ten thousands of samples can be processed in around a minute on a standard laptop. In several experiments with synthetic and real world data, superior performance in a accuracy vs. time sense compared to state-of-the-art methods, is demonstrated, which makes our algorithm a competitive GP regression approximation method.

Our approach could be enhanced in several directions. The first improvement would be more practical. While the current implementation of our algorithm works very competitively for moderate large datasets (on a standard laptop), further work has been done to scale it up to very large datasets. The current limitations are particularly factorizing the sparse block Cholesky matrices. We are convinced, that the theoretical properties of our algorithm—in particular the linearity in the number of experts and data samples—enables large scale implementations when exploiting more low level linear algebra tools. Another interesting direction would be to investigate the connection of our sparse precision matrix to state space systems, such that sequential learning algorithm could be exploited, which is briefly outlined in D. Further, it would be interesting to apply variational methods to our model, so that a connection to full GP in a posterior sense might be established, where some ideas are outlined in A.1.

## Supplementary Information

Below is the link to the electronic supplementary material.

Supplementary file 1 (pdf 1433 KB)

## Data Availability

The data used in this paper is available on public data repositories as indicated in the supplementary material.

## Footnotes

1 We use the algorithms in https://scikit-learn.org.

2 https://github.com/manschuer/CPoE/blob/main/experiments/comparison_non_GP.ipynb.

## Code availability

We provide the code of the proposed algorithms in the paper on https://github.com/manschuer/CPoE including descriptions how to use it and the performed experiments.
