# Supplementary material for: Correlated product of experts for sparse Gaussian process regression
Source: Mach Learn. 2023 Jan 25;112(5):1411–32. doi: 10.1007/s10994-022-06297-3 (PMC10163145; doi:10.1007/s10994-022-06297-3)
Supplement: Supplementary file 1 — (pdf 1433 KB) [file 10994_2022_6297_MOESM1_ESM.pdf]

Appendix A Extensions and Details

A.1 Generalized CPoE

Alternatively to the graphical model defined in Def. 4 (and more precisely in Prop. 1 with Proof 1) which recovers sparse global GP model FITC [7] in the limiting case $C \to J$ (as shown in Prop. 5), we present in this section a generalization of our CPoE model such that it recovers other sparse global GP models such as VFE [5] or PEP [6]. As shown by the authors in [11] for the global case, these model differ in the training only by the choice of the projection matrix $\overline{\boldsymbol{V}}_j$ in Def. 4 and in the hyperparameter optimization by a modification of the log marginal likelihood $\mathcal{L}(\boldsymbol{\theta}) = \log q(\boldsymbol{y}|\boldsymbol{\theta})$ in Section 3.6.2. These two changes can also be made for our local sparse CPoE model. In particular, using $\overline{\boldsymbol{V}}_j$ and λ_j according to the values in Table A2 in the projection conditional

$$p\left(\boldsymbol{f}_j|\boldsymbol{a}_{\psi(j)}\right) = \mathcal{N}\left(\boldsymbol{f}_j|\boldsymbol{H}_j\boldsymbol{a}_{\psi(j)}, \overline{\boldsymbol{V}}_j\right)$$

and in a lower bound to the log marginal likelihood

$$\tilde{\mathcal{L}}(\boldsymbol{\theta}) = \mathcal{L}(\boldsymbol{\theta}) - \sum_{j=1}^{J} \lambda_j(\boldsymbol{\theta})$$

and $\tilde{l}_j(\boldsymbol{\theta}) = l_j(\boldsymbol{\theta}) - \lambda_j(\boldsymbol{\theta})$ in the deterministic and stochastic case, respectively, generalizes the CPoE method such that for $C \to J$ we recover the mentioned method global methods in Table A2. Thereby, we used

$$\boldsymbol{D}_j = \boldsymbol{K}_{\boldsymbol{X}_j\boldsymbol{X}_j} - \boldsymbol{K}_{\boldsymbol{X}_j\boldsymbol{A}_{\psi(j)}}\boldsymbol{K}^{-1}_{\boldsymbol{A}_{\psi(j)}\boldsymbol{A}_{\psi(j)}}\boldsymbol{K}_{\boldsymbol{A}_{\psi(j)}\boldsymbol{X}_j}$$

which is the difference of the true and local approximated covariance.

The setting in VFE [5] is particularly interesting, since it constitutes in the global case a direct posterior approximation derived via a variational maximization of the lower bound of the log marginal likelihood. Moving a bit away from the true marginal likelihood of full GP has the effect that overfitting (w.r.t. full GP) can not happen when optimizing the hyperparameters with the lower bound. This is particularly important when all inducing inputs are optimized as it is usually recommended in sparse global methods which is not the case for our model since it allows to have a number of inducing points in the order of the number of data samples. In the adapted 'local VFE' CPoE model when using $\overline{\boldsymbol{V}}_j = 0$ and minimize also $\lambda_j = tr\{\boldsymbol{D}_j\}$ has the effect that the model is locally variationally optimal, however, it would be interesting to directly derive a lower bound analogously to [5] so that the posterior of our CPoE model is rigorously connected to full GP. Since this is not a straight-forward extension, we postpone this task to future work. Below, we present the connection to full GP for this adapted model in the joint prior sense analogously to Prop. 6 for the local FITC model.

variable	domain	explanation
N	$\mathbb{N}^+$	number of data samples
D	$\mathbb{N}^+$	number of variables/dimension of data
J	$\{1,\ldots,N\}$	number of experts/partitions
B	$\{1,\ldots,N\}$	size of expert/partition
L	$\{1,\ldots,B\}$	number of local inducing points
M	$\{1,\ldots,N\}$	number of total local inducing points
C	$\{1,\ldots,J\}$	degree of correlation
γ	$(0,1]$	sparsity parameter
α	$(0,J]$	approximation quality parameter
y_i	$\mathbb{R}$	individual data output
$\boldsymbol{y}_j$	$\mathbb{R}^B$	data output of expert j
$\boldsymbol{y}_{k:j}$	$\mathbb{R}^{B(j-k1+)}$	data output of experts k up to j
y_*	$\mathbb{R}$	pointwise noisy prediction output
$\boldsymbol{y}$	$\mathbb{R}^N$	all data output
$\boldsymbol{x}_i$	$\mathbb{R}^D$	individual data indput
$\boldsymbol{X}_j$	$\mathbb{R}^{B\times D}$	data input of expert j
$\boldsymbol{X}_{k:j}$	$\mathbb{R}^{B(j-k+1)\times D}$	data input of experts k up to j
$\boldsymbol{X}$	$\mathbb{R}^{N\times D}$	all data input
$\boldsymbol{x}_*$	$\mathbb{R}^D$	query input for prediction
$\boldsymbol{f}_j$	$\mathbb{R}^B$	latent function outputs of expert j
$\boldsymbol{f}_{k:j}$	$\mathbb{R}^{B(j-k+1)}$	latent function outputs of experts k up to j
$\boldsymbol{f}$	$\mathbb{R}^N$	all latent function outputs
f_*	$\mathbb{R}$	pointwise (latent) prediction output
$f(\boldsymbol{X}_j)$	$\mathbb{R}^B$	GP evaluation for input matrix
$\boldsymbol{a}_j$	$\mathbb{R}^L$	local inducing outputs of expert j
$\boldsymbol{a}_{k:j}$	$\mathbb{R}^{L(j-k+1)}$	local inducing outputs of experts k up to j
$\boldsymbol{a}$	$\mathbb{R}^M$	all local inducing outputs
$\boldsymbol{A}_j$	$\mathbb{R}^{L\times D}$	local inducing inputs of expert j
$\boldsymbol{A}_{k:j}$	$\mathbb{R}^{L(j-k+1)\times D}$	local inducing inputs of experts k up to j
$\boldsymbol{A}$	$\mathbb{R}^{M\times D}$	all local inducing inputs
I_j	$\{1,\ldots,C-1\}$	number of predecessors of expert j
$\boldsymbol{\phi}_i(j)$	$\{1,\ldots,j-1\}$	ith predecessor of expert j
$\boldsymbol{\pi}(j)$	$\{1,\ldots,j-1\}^{I_j}$	predecessor index set
$\boldsymbol{\pi}^+(j)$	$\{1,\ldots,j\}^{I_j+1}$	predecessor index set including j
$\boldsymbol{\psi}(j)$	$\{1,\ldots,max(j,C)\}^C$	correlation index set
σ_n^2	$\mathbb{R}^+$	observation noise variance
$\boldsymbol{\theta}$	$\mathbb{R}^{\lvert\boldsymbol{\theta}\rvert}$	kernel hyperparameters including σ_n^2
$k_{\boldsymbol{\theta}}(\boldsymbol{x},\boldsymbol{x}')$	$\mathbb{R}$	kernel evaluation for 2 query points
$\boldsymbol{K}_{\boldsymbol{AB}}$	$\mathbb{R}^{M_a\times M_b}$	kernel matrix of two query matrices
$p(\boldsymbol{z})$	$\mathbb{R}$	evaluation of (true) probability density
$q(\boldsymbol{z})$	$\mathbb{R}$	evaluation of approximated probability density
$\boldsymbol{S}$	$\mathbb{R}^{M\times M}$	prior precision matrix
$\boldsymbol{T}$	$\mathbb{R}^{M\times M}$	projection precision matrix
$\boldsymbol{\Sigma}^{-1}$	$\mathbb{R}^{M\times M}$	posterior precision matrix
$\boldsymbol{\Sigma}$	$\mathbb{R}^{M\times M}$	posterior covariance matrix
$\boldsymbol{\mu}$	$\mathbb{R}^M$	posterior mean vector
$\boldsymbol{\mu}_{\boldsymbol{\psi}(j)}$	$\mathbb{R}^{CL}$	local posterior mean
$\boldsymbol{\Sigma}_{\boldsymbol{\psi}(j)}$	$\mathbb{R}^{CL\times CL}$	local posterior covariance
$\boldsymbol{F}$	$\mathbb{R}^{M\times M}$	prior transition matrix
$\boldsymbol{Q}$	$\mathbb{R}^{M\times M}$	prior noise matrix
$\boldsymbol{H}$	$\mathbb{R}^{N\times M}$	projection matrix
$\overline{\boldsymbol{V}}$	$\mathbb{R}^{N\times N}$	projection noise matrix
$\boldsymbol{V}$	$\mathbb{R}^{N\times N}$	projection noise matrix including observation noise
$\boldsymbol{P}$	$\mathbb{R}^{N\times N}$	marginal likelihood covariance matrix
J_2	$\mathbb{N}^+$	number of prediction experts
$\bar{\beta}_{*j}$	$\mathbb{R}^+$	unnormalized predictive weight of expert j at $\boldsymbol{x}_*$
β_{*j}	$\mathbb{R}^+$	normalized predictive weight of expert j at $\boldsymbol{x}_*$
m_{*j}	$\mathbb{R}$	predictive mean of expert j at $\boldsymbol{x}_*$
v_{*j}	$\mathbb{R}^+$	predictive variance of expert j at $\boldsymbol{x}_*$
$\mathbb{D}_{[C,C_2]}$	$\mathbb{R}^+$	difference in KL between two approximate models

Table 1: Overview of notation.

	$\overline{\boldsymbol{V}}_j$	λ_j
DTC	0	0
FITC	$Diag[\boldsymbol{D}_j]$	0
PITC	$\boldsymbol{D}_j$	0
VFE	0	$\frac{1}{2\sigma_n^2} tr[\boldsymbol{D}_j]$
PEP	$\alpha Diag[\boldsymbol{D}_j]$	$\frac{1-\alpha}{2\alpha} \sum_i \log\left(1 + \frac{\alpha}{\sigma_n^2} \boldsymbol{D}_j^{(i)}\right)$
PEP_B	$\alpha \boldsymbol{D}_j$	$\frac{1-\alpha}{2\alpha} \sum_i \log \lvert \mathbb{I} + \frac{\alpha}{\sigma_n^2} \boldsymbol{D}_j \rvert$

Table A2: Generalizations of CPoE model.

Proposition 7 (Local VFE) *Using a deterministic projection* $q(\boldsymbol{f}_j | \boldsymbol{a}_{\psi(j)}) = \mathcal{N}\left(\boldsymbol{f}_j | \boldsymbol{H}_j \boldsymbol{a}_{\psi(j)}, \overline{\boldsymbol{V}}_j\right)$ *in the graphical model in Def. 4 and Prop. 1, that is, setting the covariance* $\overline{\boldsymbol{V}}_j = 0$ *in the projection step recovers global VFE for* $C \to J$*. Moreover, the difference in KL to full GP of the joint prior is also decreasing. In particular, the difference in KL of the prior of the local VFE model for* $1 \le C \le C_2 \le J$ *is*

$$\mathbb{D}_{(C,C_2)}[\boldsymbol{a}] = \frac{1}{2} \log \frac{|\boldsymbol{Q}_C|}{|\boldsymbol{Q}_{C_2}|} \ge 0.$$

Further, the difference in KL of the projection is

$$\mathbb{D}_{(C,C_2)}[\boldsymbol{y}|\boldsymbol{a}] = \frac{1}{2\sigma_n^2} tr\{\bar{\boldsymbol{V}}_C - \bar{\boldsymbol{V}}_{C_2}\} \ge 0.$$

The overall prior approximation quality is

$$\mathbb{D}_{(C,C_2)}[\boldsymbol{a}, \boldsymbol{y}] = \frac{1}{2} \log \frac{|\boldsymbol{Q}_C|}{|\boldsymbol{Q}_{C_2}|} + \frac{1}{2\sigma_n^2} tr\{\bar{\boldsymbol{V}}_C - \bar{\boldsymbol{V}}_{C_2}\}$$

where

$$tr\{\bar{\boldsymbol{V}}_C\} = \sum_{i=1}^{N} K_{\boldsymbol{X}_i \boldsymbol{X}_i} - K_{\boldsymbol{X}_i \boldsymbol{A}_{\boldsymbol{\psi}(j_i)}} K^{-1}_{\boldsymbol{A}_{\boldsymbol{\psi}(j_i)} \boldsymbol{A}_{\boldsymbol{\psi}(j_i)}} K_{\boldsymbol{A}_{\boldsymbol{\psi}(j_i)} \boldsymbol{X}_i}.$$

Compared to the FITC model is the difference in the trace instead of the fraction of the log-determinants.

A.2 Solving Linear System & Partial Inversion

For solving the sparse linear system $\boldsymbol{\Sigma}^{-1}\boldsymbol{\mu} = \boldsymbol{\eta}$ in Prop. 3, sparse Cholesky decomposition is exploited, that is, $\boldsymbol{M}\boldsymbol{\Sigma}^{-1}\boldsymbol{M}^T = \boldsymbol{L}\boldsymbol{L}^T =: \boldsymbol{Y}$ is computed so that $\boldsymbol{\nu}$ and $\overline{\boldsymbol{\mu}}$ can be efficiently obtained via solving $\boldsymbol{L}\boldsymbol{\nu} = \boldsymbol{\eta}$ and $\boldsymbol{L}^T\overline{\boldsymbol{\mu}} = \boldsymbol{\nu}$, respectively, where $\boldsymbol{M}$ is a so-called fill-reduction permutation matrix such that the Cholesky matrix $\boldsymbol{L}$ is as sparse as possible and thus $\boldsymbol{\mu} = \boldsymbol{M}^{-1}\overline{\boldsymbol{\mu}}$. Note that $\boldsymbol{M}$ is computed only via the structure on the block level which is only J dimensional instead of JL. Additionally to the mean $\boldsymbol{\mu}$, also some entries $\boldsymbol{\Sigma}_{\boldsymbol{\psi}(j)}$ in the covariance matrix $\boldsymbol{\Sigma}$ has to be explicitly computed, which are needed for computing local predictions used in Section 3.4.

Proposition 8 (Local Predictions, Proof 18) *The local prediction* $q_{c,\gamma}(f_{*j}|\boldsymbol{y}) = \mathcal{N}\left(m_{*j}, v_{*j}\right)$ *of the* j*th expert are based on the region* $\boldsymbol{\psi}(j)$*, where the correlations are modelled and can be computed as*

$$q_{c,\gamma}(f_{*j}|\boldsymbol{y}) = \int p\left(f_{*j}|\boldsymbol{a}_{\boldsymbol{\psi}(j)}\right) q_{c,\gamma}(\boldsymbol{a}_{\boldsymbol{\psi}(j)}|\boldsymbol{y})\,\mathrm{d}\boldsymbol{a}_{\boldsymbol{\psi}(j)} = \mathcal{N}\left(\boldsymbol{h}_*\boldsymbol{\mu}_{\boldsymbol{\psi}(j)}, \boldsymbol{h}_*^T\boldsymbol{\Sigma}_{\boldsymbol{\psi}(j)}\boldsymbol{h}_* + v_*\right)$$

involving the local posteriors $q_{c,\gamma}(\boldsymbol{a}_{\boldsymbol{\psi}(j)}|\boldsymbol{y}) = \mathcal{N}\left(\boldsymbol{\mu}_{\boldsymbol{\psi}(j)}, \boldsymbol{\Sigma}_{\boldsymbol{\psi}(j)}\right)$ *and the predictive conditional* $p\left(f_{*j}|\boldsymbol{a}_{\boldsymbol{\psi}(j)}\right) = \mathcal{N}\left(\boldsymbol{h}_*\boldsymbol{a}_{\boldsymbol{\psi}(j)}, v_*\right)$ *(which is exactly defined in Proof 18).*

For computing the local posteriors $q_{c,\gamma}(\boldsymbol{a}_{\boldsymbol{\psi}(j)}|\boldsymbol{y}) = \mathcal{N}\left(\boldsymbol{\mu}_{\boldsymbol{\psi}(j)}, \boldsymbol{\Sigma}_{\boldsymbol{\psi}(j)}\right)$, we need to compute the entries $\boldsymbol{\Sigma}_{\boldsymbol{\psi}(j)}$ in the covariance matrix $\boldsymbol{\Sigma}$, which correspond to the non-zeros in the precision matrix $\boldsymbol{\Sigma}^{-1}$. Computing efficiently these entries is not straightforward since in the inverse the blocks are no longer independent. However, we can exploit the particular sparsity and block-structure of our precision matrix and obtain an efficient implementation of this part which is key to achieve a competitive performance of our algorithm.
Computing some entries in $\boldsymbol{Z} = \boldsymbol{Y}^{-1}$ is also known as *partial inversion.* We adapted the approach in [38] where the recursive equations with J blocks for computing the full inverse $\boldsymbol{Z}$ are provided

$$\boldsymbol{Z}_{B_j} = -\boldsymbol{Z}_{C_j}\boldsymbol{L}_{B_j}\boldsymbol{L}_{A_j}^{-1} \qquad \text{and} \qquad \boldsymbol{Z}_{A_j} = \boldsymbol{L}_{A_j}^{-T}\boldsymbol{L}_{A_j}^{-1} - \boldsymbol{Z}_{B_j}^T\boldsymbol{L}_{B_j}\boldsymbol{L}_{A_j}^{-1}$$

where the recursion starts from $j = J$ with $\boldsymbol{Z}_{A_J} = \boldsymbol{L}_{A_J}^{-T}\boldsymbol{L}_{A_J}^{-1}$.

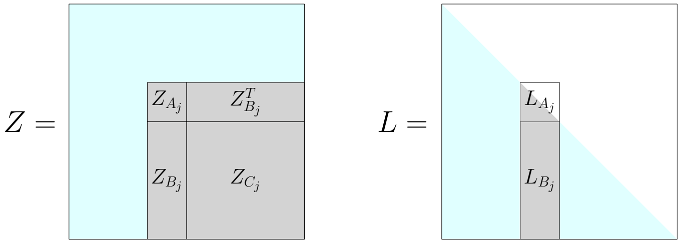

Instead of computing the full inverse using this recursion, we exploited the block-sparsity structure of our posterior precision matrix in order to gain significant speed-up. We only computed the entries in the inverse $\boldsymbol{Z}$ which are symbolically non-zero in $\boldsymbol{L}$. In Algorithm 1 in the Appendix we provide efficient pseudo-code using sparse-block-matrices in the block-sparse-row format.

Alternatively for computing the Cholesky factor of $\boldsymbol{\Sigma}^{-1} = \boldsymbol{S} + \boldsymbol{H}\boldsymbol{V}^{-1}\boldsymbol{H}$, we could directly exploit that the prior precision $\boldsymbol{S} = \boldsymbol{F}^T\boldsymbol{Q}^{-1}\boldsymbol{F}$ is already decomposed into a upper/lower-triangular form since $\boldsymbol{F}$ lower triangular. However, when updating the Cholesky factor with $\boldsymbol{H}\boldsymbol{V}^{-1}\boldsymbol{H}$ needs quadratic time in the number of nonzeros for each expert.

Algorithm 1 Partial Sparse Block Inversion

Require: Cholesky matrix $\boldsymbol{L}$ of size $JB \times JB$ in block-sparse-row (bsr) format with $J \times J$ total blocks, block-size B and N non-zero blocks. Data array d of size $N \times B \times B$, the column-block-indices r of size N, row-block-pointer p of length $J+1$, and lookup table M of dimension $J \times J$.

Ensure: The lower part of the symmetric partial inversion is computed in bsr-format with the same row-block-indices r and row-block-pointer p and data array z of size $N \times B \times B$.

```
for i ∈ {J, ..., 1} do
    L_A^{-1} ← d[M[i,i], :, :]^{-1}
    z[M[i,i]] ← (L_A^{-1})^T · L_A^{-1}
    for j ∈ {r[p[i+1]], r[p[i+1] − 1], ..., r[p[i]]} do
        Q ← 0
        for l ∈ {r[p[i]], r[p[i] + 1], ..., r[p[i+1]]} do
            R ← z[M[j,l]]
            if l > j then
                R ← R^T
            end if
            Q ← Q + R · d[M[l,i]] · L_A^{-1}
        end for
        z[M[i,j]] ← z[M[i,j]] − Q
    end for
end for
```

A.3 Hyperparameter Estimation

In Section 3, we introduced CPoE for fixed hyperparameters $\boldsymbol{\theta}$ where implicitly all distributions are conditioned on $\boldsymbol{\theta}$, however, we omitted the dependencies on $\boldsymbol{\theta}$ in the most cases for the sake of brevity. Similar to full GP, sparse GP or PoEs, the *log marginal likelihood (LML)* can be used as an objective function for optimizing the few hyperparameters $\boldsymbol{\theta}$.

A.3.1 Deterministic Optimization

The log of the marginal likelihood of our model formulated in Section 3.3 can be written as

$$\mathcal{L}(\boldsymbol{\theta}) = \log q\left(\boldsymbol{y}|\boldsymbol{\theta}\right) = \log \mathcal{N}\left(\boldsymbol{0}, \boldsymbol{P}\right) = -\frac{1}{2}\left(\boldsymbol{y}^T \boldsymbol{P}^{-1} \boldsymbol{y} + \log |\boldsymbol{P}| + N \log 2\pi\right)$$

with $\boldsymbol{P} = \boldsymbol{H}\boldsymbol{S}^{-1}\boldsymbol{H}^T + \boldsymbol{V}$. Since $\boldsymbol{P}$ is dense, we can apply the inversion (B2) and determinant lemma (B3) to $\boldsymbol{P}$ and exploit $|\boldsymbol{F}| = 1$ yielding

$$\begin{aligned} \mathcal{L}(\boldsymbol{\theta}) &= -\frac{1}{2}\left(\boldsymbol{y}^T\boldsymbol{V}^{-1}\boldsymbol{y} - \boldsymbol{\mu}^T\boldsymbol{\Sigma}^{-1}\boldsymbol{\mu} + \log\frac{|\boldsymbol{\Sigma}^{-1}|\,|\boldsymbol{V}|}{|\boldsymbol{S}|} + N\log 2\pi\right) \\ &= -\frac{1}{2}\left(\boldsymbol{y}^T\boldsymbol{V}^{-1}\boldsymbol{y} - \boldsymbol{\mu}^T\boldsymbol{\Sigma}^{-1}\boldsymbol{\mu} + \log|\boldsymbol{\Sigma}^{-1}|\,|\boldsymbol{V}|\,|\boldsymbol{Q}| + N\log 2\pi\right) \end{aligned} \tag{A1}$$

so that all involved quantities $\boldsymbol{\Sigma}^{-1}$, $\boldsymbol{V}$ and $\boldsymbol{Q}$ are sparse. For efficient parameter minimization, the derivative of the log marginal likelihood with respect to each parameter in $\boldsymbol{\theta}$ is needed for which the derivations are provided in Appendix C.3. Thereby also some parts of the covariance matrix $\boldsymbol{\Sigma}$ are needed which is explained in Section A.2. Alternatively to the marginal likelihood, we can maximize a lower bound of it which is a generalization of our model so that we recover a range of well known sparse global GP models for $C \to J$ as discussed is Section A.1. [6, 11].

For moderate sample size N, *deterministic optimization* with full batch $\boldsymbol{y}$ can be performed. That means, the log marginal likelihood for the whole data is computed for which the sparse system of equations with the sparse posterior precision as well as the partial inversion of the posterior covariance has to be solved. In particular, the functions for computing $\mathcal{L}(\boldsymbol{\theta})$ and $\frac{\partial\mathcal{L}(\boldsymbol{\theta})}{\partial\boldsymbol{\theta}}$ for each $\boldsymbol{\theta}$ and full data $\boldsymbol{y}$ are repetitively called by a numerical minimizer. Fig. A1 illustrates the performance of this deterministic batch hyperparameter optimization where the convergence for the log marginal likelihood, average KL divergence, 95%-coverage (both quantities exactly defined in Appendix F) for different number of experts J compared to full GP are depicted. The $N = 2048$ data samples are generated with a $D = 2$-dimensional SE-kernel and the test KL and coverage mean values are reported for $N_{test} = 1000$ samples with 5 repetitions. We used $\gamma = 1$ and $C \in \{1, \ldots, 7\}$. We observe that the log marginal likelihood and KL are getting better for increasing C, and the deterministic parameter estimates converge to the ones of full GP for increasing function calls. It is interesting to observe that also for smaller C values, the coverage of our methods are consistent. In particular, they are slightly too big, meaning our confidence information are conservative. This is due to the aggregation based on the covariance intersection method with normalized weights, which guarantees consistent second order information.

A.3.2 Stochastic Optimization

The presented method in the previous section works fine for small datasets, however, in order to scale this parameter optimization part to larger number of samples N in a competitive time, *stochastic optimization* techniques has to be exploited similarly done for the global sparse GP model (SVI [9]; REC [11]; IF [10]). In the approximation method REC [11], the recursive derivatives are exactly propagated which would also be possible for our model, however, it

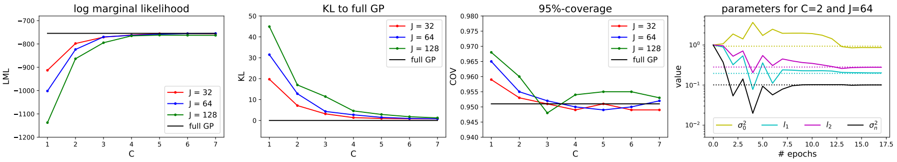

Fig. A1: Convergence of deterministic batch hyperparameter optimization for increasing C and trace of the parameters (solid) compared to the optimal values of full GP (dotted).

turned out that in practice the differences in accuracy are very small when using instead the hybrid approach IF of [10]. Thereby, the independent factorization of the log marginal likelihood is used for the computations of the optimization part, whereas the exact posterior is used for inference and prediction. Adapted to our setting, the independent factorized log marginal likelihood $\log q(\boldsymbol{y}|\boldsymbol{\theta})$ can be approximated by

$$\begin{aligned}\log q(\boldsymbol{y}|\boldsymbol{\theta}) &\approx \log \prod_{j=1}^{J} \int q\left(\boldsymbol{y}_j|\boldsymbol{a}_j\right) q(\boldsymbol{a}_j)\, \mathrm{d}\boldsymbol{a}_j \\ &= \log \prod_{j=1}^{J} \int \mathcal{N}(\boldsymbol{H}_j\boldsymbol{a}_j, \boldsymbol{V}_j)\, \mathcal{N}\left(\boldsymbol{0}, \boldsymbol{S}_j^{-1}\right) \mathrm{d}\boldsymbol{a}_j \\ &= \sum_{j=1}^{J} \log \mathcal{N}(\boldsymbol{0}, \boldsymbol{P}_j) =: \tilde{\mathcal{L}}(\boldsymbol{\theta})\end{aligned}$$

where $\boldsymbol{P}_j = \boldsymbol{H}_j\boldsymbol{S}_j^{-1}\boldsymbol{H}_j^T + \boldsymbol{V}_j$ with $\boldsymbol{S}_j = \boldsymbol{K}_{\boldsymbol{A}_j\boldsymbol{A}_j}^{-1}$. The difference compared to the deterministic case in (A1) and to [10] for the global sparse model is the independent prior $q(\boldsymbol{a}_j)$ instead of $q(\boldsymbol{a})$ and $p(\boldsymbol{a})$, respectively. In the approximate case, we can write

$$\tilde{\mathcal{L}}(\boldsymbol{\theta}) = -\frac{1}{2} N \log(2\pi) + \sum_{j=1}^{J} l_j(\boldsymbol{\theta})$$

with $l_j(\boldsymbol{\theta}) = -\frac{1}{2}\left(\boldsymbol{y}_j^T\boldsymbol{P}_j^{-1}\boldsymbol{y}_j + \log|\boldsymbol{P}_j|\right)$ which has the advantage that it decomposes into the J terms l_j in the sum, so that it can be used for stochastic optimization. This constitutes a very fast and accurate alternative for our method as shown in Figure A2 and is exploited in Section 4 for large data sets.

A.3.3 Prior on Hyperparameters

Alternatively to the log marginal likelihood (LML) maximization as presented above, the *maximum a posteriori* (MAP) estimator for $\boldsymbol{\theta}$ can be used. It is the log of the posterior distribution $p(\boldsymbol{\theta}|\boldsymbol{y}) \propto p(\boldsymbol{y}|\boldsymbol{\theta})\, p(\boldsymbol{\theta})$ where $p(\boldsymbol{\theta})$ is a suitable prior on the hyperparameters yielding $\log p(\boldsymbol{\theta}|\boldsymbol{y}) = \log p(\boldsymbol{y}|\boldsymbol{\theta}) + \log p(\boldsymbol{\theta})$.

In the following, we assume $p(\boldsymbol{\theta}) = \prod_j p(\boldsymbol{\theta}_j)$ and a log-normal prior for each hyperparameter $p(\boldsymbol{\theta}_j) = \log \mathcal{N}\left(\boldsymbol{\theta}_j | \nu_j, \lambda_j^2\right)$ for means ν_j and variances λ_j^2. For the deterministic case, the MAP estimator can be straightforwardly computed by just adding the log prior on $\boldsymbol{\theta}$ to the batch log marginal likelihood, i.e. $\log p(\boldsymbol{\theta}|\boldsymbol{y}) = \mathcal{L}(\boldsymbol{\theta}) + \log p(\boldsymbol{\theta})$. Similarly for the stochastic case, the stochastic MAP can be decomposed as $\log p(\boldsymbol{\theta}|\boldsymbol{y}) \approx \sum_{j=1}^{J} \left(l_j(\boldsymbol{\theta}) + \frac{1}{J}\log p(\boldsymbol{\theta})\right)$, where $l_j(\boldsymbol{\theta})$ is the jth term in the stochastic marginal likelihood , so that it can be used again for stochastic mini-batch optimization. An example using priors for the hyperparameters is presented in Section 4.4.

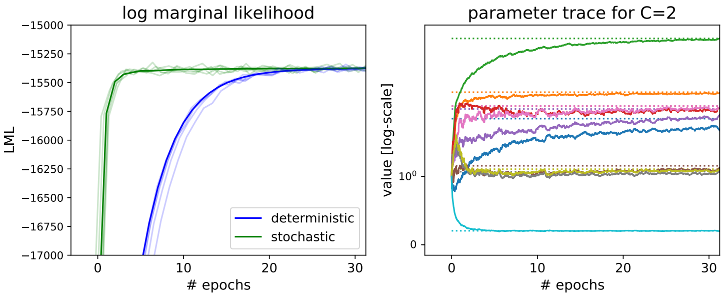

Fig. A2: Convergence of stochastic vs. deterministic hyperparameter optimization of our model CPoE. This experiment compares the convergence of stochastic vs. deterministic hyperparameter optimization for the log marginal likelihood and the trace of the 10 parameters $\boldsymbol{\theta}_j$ for the dataset *cadata* with $N_{tot} = 20640$ and $D = 8$. We used 5 different splits with $N = 0.9N_{tot}$ training data and the rest for testing. The values for our algorithm are $C = 2$, $J = 64$, $\gamma = 1$ and learning rate $\delta = 0.01$. In the right plot, the dotted horizontal lines and the solid traces correspond to the final deterministic value and the current stochastic values, respectively. We note that the stochastic LML and trace of hyperparameters converge faster to a very similar value as in the deterministic case.

A.4 Complexity

The time complexity for computing the posterior and the marginal likelihood in our algorithm is dominated by J operations which are cubic in LC (inversion, matrix-matrix multiplication, determinants). This leads to $\mathcal{O}(J(LC)^3) = \mathcal{O}(J(BC\gamma)^3) = \mathcal{O}(NB^2\alpha^3)$ where we define the approximation quality parameter $\alpha = C\gamma$. Similarly for the needed space $\mathcal{O}(J(LC)^2) = \mathcal{O}(J(BC\gamma)^2) = \mathcal{O}(NB\alpha^2)$. For N_t testing points, the time for (pointwise) predictions is dominated by J inversions of matrices with dimension LC and matrix multiplications with dimensions $LC \times LC \times N_t$ leading to $\mathcal{O}(J(LC)^3 + J(LC)^2N_t) = \mathcal{O}(J(B\gamma C)^3 + J(B\gamma C)^2N_t) = \mathcal{O}(NB^2\alpha^3 + NB\alpha^2N_t)$ where the operations independent of the test points can be precomputed in the inference part leading to $\mathcal{O}(NB\alpha^2N_t)$ for testing. Similarly for the space. A further reduction in complexity would be achieved if the product over all experts

in Prop. 4 is approximated only with the $W < J$ nearest experts, leading to $\mathcal{O}((LC)^2 N_t W) = \mathcal{O}((B\gamma C)^2 N_t W) = \mathcal{O}(N\frac{W}{J}B\alpha^2 N_t)$ time complexity for testing. This might be interesting if we want to make fast predictions for many points N_t. For reasonable values of W, for instance $W = 1$, $W = C$ or $W = Z = \log(N)C$ (used in prediction aggregation), preliminary experiments show very comparable performance. Note that the consistency properties for covariance intersection method are preserved as long as the weights are normalized over the used W experts. Table 1 compares the asymptotic complexities with other GP algorithms.

It is interesting that for $\alpha = 1$, our algorithm has the same asymptotic complexity for training as sparse global GP with $M_g = B$ global inducing points but we can have $M_l = LJ = \gamma BJ = \gamma N$ total local inducing points! Thus, our approach allows much more total local inducing points M in the order of N (e.g. $M = 0.5N$ with $C = 2$) whereas for sparse global GP usually $M_g \ll N$. This has the consequence that the local inducing points can cover the input space much better and therefore represent much more complicated functions. As a consequence, there is also no need to optimize the local inducing points resulting in much fewer parameters to optimize. Consider the following example with $N = 10'000$ in $D = 10$ dimensions. Suppose a sparse global GP model with $M_g = 500$ global inducing points. A CPoE model with the same asymptotic complexity has a batch size $B = M_g = 500$ and $\alpha = 1$. Therefore, we have $J = \frac{N}{B} = 20$ experts and we choose $C = 2$ and $\gamma = \frac{1}{2}$ such that we obtain $L = \gamma B = 250$ local inducing points per experts and $M = \gamma N = 5'000$ total inducing points! Further, the number of hyperparameters to optimize for a SE kernel is for global sparse GP $M_g D + |\boldsymbol{\theta}| = 5012$, whereas for CPoE there are only $|\boldsymbol{\theta}| = 12$.

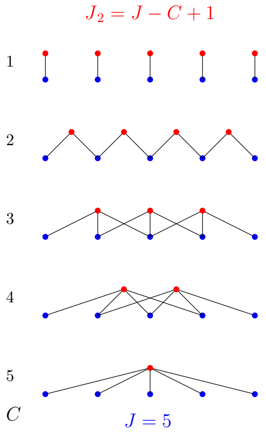

Fig. A3: Prediction Aggregation in CPoE(C, γ) model with J base experts and $J_2 = J - C + 1$ predictive experts.

For our method, the time and space complexity is linear in the number of samples N and the number of experts J which makes our approach highly scalable. The approximation quality parameter $\alpha = C\gamma$ appears cubic/quadratic in the time/space complexity. The optimal approximation quality (and thus equivalent to full GP) is achieved for $\alpha = J$ which implies $C = J$ and $\gamma = 1$. However, it is clear that this is not feasible for big datasets and thus some moderate values of C and γ have to be selected to trade off time and accuracy which is illustrated in the Appendix in Table A3 and Fig. A4.

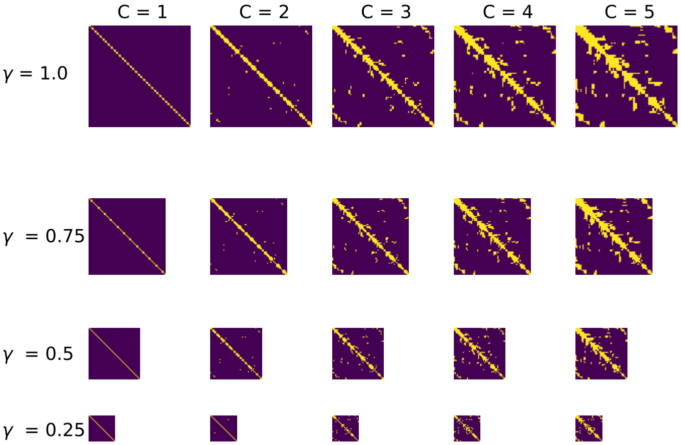

Fig. A4: Influence of the approximation order C and sparseness parameter γ to the non-zeros and size of the posterior precision matrix $\mathbf{\Sigma}^{-1}$. for an example with synthetic GP data with $D = 2$, $N = 8192$, $J = 64$ and $B = 128$ and a SE-kernel. Compare also Table A3.

**KL**	C=1	C=2	C=3	C=4	C=5
$\gamma = 1/4$	12.3	5.0	1.3	0.9	0.7
$\gamma = 1/2$	12.2	4.9	1.0	0.8	0.6
$\gamma = 3/4$	12.1	4.9	0.9	0.7	0.5
$\gamma = 1$	12.1	4.8	0.9	0.6	0.4
**time**	C=1	C=2	C=3	C=4	C=5
$\gamma = 1/4$	0.2	0.4	0.9	1.2	1.4
$\gamma = 1/2$	0.4	0.7	1.9	2.7	3.8
$\gamma = 3/4$	0.9	2.4	4.1	5.7	9.1
$\gamma = 1$	1.5	3.0	6.4	12.4	15.7

Table A3: KLs to full GP (above) and times (below) of our method CPoE for varying C and γ for experiment in Section 3.6.3. Compare also Fig. A4 .

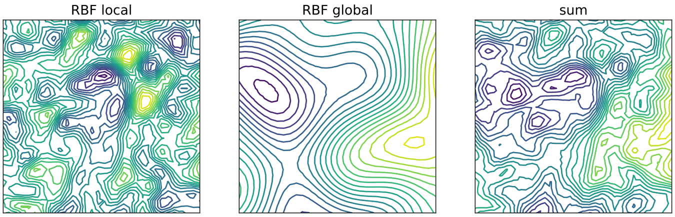

Fig. A5: Generated data with a sum of two SE-kernels with local and global length-scales for experiment in Section 4.

A.5 Relation to Deep Structured Mixtures of GPs

There are several approaches exploiting local experts, as discussed in Section 2.2. In particular, the recent approach *Deep Structured Mixtures of Gaussian Processes* (DSMGP) [29] shares some similarities with our approach. However, our approach has a main advantages against DSMGP, namely, the continuous predictive distribution, as illustrated in Figure A6. In the following, we briefly summaries their approach, discuss the similarities and differences to our approach, and compare them in two illustrative experiments.

The GP approximation approach DSMGP exploits a sum-product network of local GPs, which corresponds to a mixture of independent base experts as in the ordinary PoEs. This approach allows analytic posterior inference, however, in order to compute efficient predictions, the posterior of the DSMGP is projected to the closest GP, i.e. the GP with minimal KL divergence from the DSMGP. This has the effect that the aggregated predictive distribution might discontinuous, as illustrated in Figure A6. This is a common disadvantage of local aggregation methods based on predictions form a single region. DSMGP uses independent base experts similarly as the local PoE methods discussed in Section 2.2. However, the predictions are averaged by a mixture over different partitions of the input space. On the other hand, our approach directly models correlations between the base experts, which recovers the exact dependencies between the different partitions, leading to smooth predictions.

We compared both methods on two illustrative examples. In particular, we used the data from the main example on the GitHub repository[3] of their approach, which is illustrated on the left in Figure A6. The $N = 100$ data samples are generated from a sine and the number of splits per product nodes is $K = 4$, the number of children per sum node is $V = 3$, and the minimum number of experts is $M = 20$ corresponding to $J = 5$ base experts. We optimized the hyperparameters with full GP and fixed them for all approximation methods. The results for this kind of data are consistent with the findings in [29], where DSMGP can improve over the other local independent methods (minVar, GPoE, BCM). However, the global sparse method and in particular our method CPoE with $C = 2$ significantly outperforms DSMGP, as demonstrated with the KL to full GP. Similarly, we run their algorithm on the example of Figure 2, where the generated data has a smaller lengthscale and bigger observation noise. The parameters for DSMGP were set to $K = 4$, $V = 3$, $M = 10$, which correspond to $J = 6$ independent experts. This more complex setting illustrates the main limitation of DSMGP with the discontinuous predictions, as depicted in Figure A6 on the right. Moreover, this is also reflected in the accuracy in term of KL to full GP. We run some preliminary comparisons of the experiment depicted in Figure 9, with similar performance as obtained in the both examples shown in Figure A6. This comparison can be found on our github repository.[4] However, it is hard to compare the running times of both algorithms based on *Python* and *Julia*, respectively. Therefore, it would require

---

[3] https://github.com/trappmartin/DeepStructuredMixtures

[4] https://github.com/manschuer/CPoE/experiments/comparisonDSM_py.ipynb

to reimplement their algorithm in Python for proper empirical comparisons in a time vs. accuracy sense.

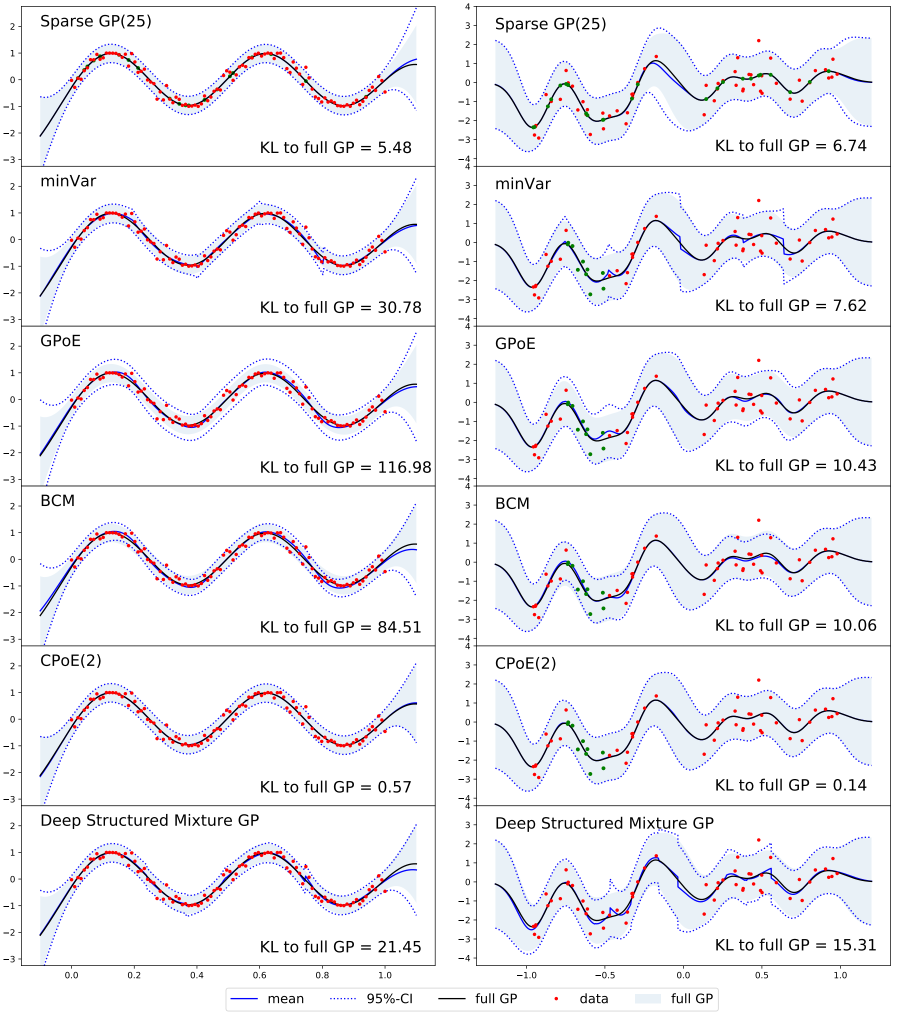

Fig. A6: The data and the example on the left corresponds exactly to the example provided on the GitHub repository of the method DSMGPs, wheres the example on the right correspons to the Example in Figure 2.

A.6 Implementation Details

All experiments were run on a standard Laptop (IntelCore i7, 8 CPU 1.9GHz). Our code is implemented in Python and is available on GitHub[5]. It contains

[5]https://github.com/manschuer/CPoE

several examples[6] [7] how to use it together with experiments from this paper. For solving the sparse linear system of equations, we used Cholmod [39] in the Python package scikit-sparse which relies on sparse Cholesky decomposition. It would be advantageous to use/implement a sparse block Cholesky decomposition and solver which exploits directly our structure. This was indeed needed for computing some entries in the posterior covariance, since with available implementation of *partial matrix inversion* we could not exploit the block sparsity and thus did not obtain competitive performance as discussed in Section A.2. An efficient implementation of this part is presented in Algorithm 1.
In our current implementation the size of each partition has to be equal; which is in theory not necessary, but it allows more efficient implementations since then the block character can be easily exploited in the computation of the sparse posterior precision. Using the KD-tree construction with $J = 2^K$, the sizes of the partitions differ at most by 1. Thus, if the partitions are not equal, the number of local inducing points are set to $L = min(B_j)_{j=1}^J$.
Our implementation exploits the kernel and likelihood functions of GPy [40]. For the optimization of the hyperparameters we used the L-BFGS-B algorithm in the Python package scipy in the deterministic full batch case. For stochastic optimization we used the stochastic optimizer ADAM [41] (implemented from scratch) with appropriate learning rates which are learnt in preliminary experiments.

For the competitor methods we used the implementation in GPy [40] for full and sparse global GP (the approach of [5]). For PoE, GPoE, BCM, RBCM and GRBCM we implemented the corresponding aggregation algorithms based on the GPy implementations for the independent experts in Python for the sake of comparisons. For the stochastic version of SGP, the hybrid information filter approach in [10] and their implementations are used. We also run the approaches REC [11] and SVI [9], however the former approach shows superior accuracy vs. time performance in preliminary experiments.
For the sparse global GP model there is the choice of optimized or fixed inducing points. For the same number of inducing points the accuracy is obviously better with optimized inducing points, however taking into account the time for optimizing them, we found in the experiments with batch optimization (i.e. also smaller datasets) that the fixed random subset approach was superior. Therefore we report here the results for fixed (random subset of data) inducing points in the deterministic case and optimized in the stochastic case. The reason for that is that the sparse global approximation with unknown inducing inputs has $MD + |\boldsymbol{\theta}|$ (variational) parameters to optimize in the batch version. In the stochastic version REC & IF there are as well $MD + |\boldsymbol{\theta}|$ parameters, whereas SVI has even $M + 0.5M^2 + MD + |\boldsymbol{\theta}|$ number of parameters since the posterior mean and covariance has to be optimized.

[6] https://github.com/manschuer/CPoE/experiments/example_1D.ipynb
[7] https://github.com/manschuer/CPoE/experiments/example_2D.ipynb

On the other hand, full GP has only a few kernel hyperparameters $|\boldsymbol{\theta}|$ to optimize. Similarly, our method CPoE (and also independent PoEs) inherit this property because there is no necessity to optimize the local inducing points since the total amount of them can be in the order of N. This is also true for the stochastic version of our algorithm. Assume for instance $D = 8$ and $M = 100$, the number of parameters with a SE kernel for full GP and CPoE are only $|\boldsymbol{\theta}| = 10$ parameters to optimize, whereas for batch SGP, REC & IF 810 and even 5910 for SVI. For fixed inducing points, SGP and IF also only have $|\boldsymbol{\theta}| = 10$ hyperparameters which allows to have more inducing points but speed-up the optimization a lot and makes the accuracy vs. time comparison more competitive.

We used the KD-partition for our method as discussed in 3.1 while in the PoE-literature [12–16], often K-Means is used for partitioning. However, for large J and N this is quite inefficient and often the partition sizes for each expert differs significantly which introduces an imbalance among the experts in the prediction aggregation as well as in the stochastic optimization. Therefore we also used the KD-tree partition for these algorithms for the sake of comparisons.

For assessing the quality of the different algorithms in the next sections, we report the two quantities *the Kullback-Leibler-(KL)-divergence* to full GP and the *Continuous Ranked Probability Score (CRPS)* both depending on the pointwise predictive distributions $p(f_*|\boldsymbol{y})$. The reported values correspond always to an average of N_{test} prediction points which are not contained in the training data.

A.7 Experiments

In this section, we provide more details about the experiments in Section 4.

A.7.1 Synthetic Data

In this simulation study with synthetic GP data, we examine the accuracy vs. time performance of different GP algorithms for fixed hyperparameters. We generated $N = 8192$ data samples in $D = 2$ with 5 repetitions from the sum of two SE kernels with a shorter and longer lengthscale ($l_s = 0.125, v_s = 0.2$ and $l_l = 0.5, v_l = 1.1$; see Fig. A5) such that both global and local patterns are present in the data. In Fig. 9 the mean results are shown for the KL and RMSE to full GP, the 95%-coverage and the log marginal likelihood against time in seconds.
For the sparse GP, we use different number of fixed global inducing points $M = \{50, \dots, 1000\}$ for which the results are shown in blue.[8] From the PoE-family, the results for minVar, GPoE and BCM are depicted for different number of

[8] We also run sparse GP with optimized inducing points, however the performance compared to time was worse.

Springer Nature 2021 LaTeX template

	**CRPS**				**time**			
	kin2	cadata	sarcos	casp	kin2	cadata	sarcos	casp
SGP(500)	0.183	0.253	0.069	0.329	112.1	346.9	730.1	632.9
SGP(1000)	0.166	0.252	0.063	0.325	244.1	727.6	1718.5	1362.5
minVar	0.173	0.257	0.052	0.294	14.4	28.2	71.3	45.8
GPoE	0.193	0.289	0.086	0.302	14.4	28.3	71.4	45.6
GRBCM	0.164	0.262	0.060	0.310	16.5	33.5	84.6	59.4
**CPoE(1)**	0.163	0.259	0.052	0.289	13.8	24.5	45.4	45.1
**CPoE(2)**	0.155	0.251	0.051	0.287	18.9	33.4	67.3	70.3
**CPoE(3)**	**0.151**	**0.249**	**0.051**	**0.282**	31.7	52.0	134.3	123.8

Table A4: Average CRPS (left) and time (right) for different GP methods and 4 datasets with 5 repetitions. More details and results are provided in Sections A.7.2 and F in the Appendix.

experts $J = \{1, 2, 4, \dots, 128\}$ in red, cyan and magenta, respectively. For our correlated PoEs, the results for the correlations $C = \{1, \dots, 12\}$ are shown in green for $J = 32$ and $\gamma = 0.5$.
In the first two plots, the superior performance of our method compared to competitors in accuracy to full GP vs. time can be observed. Our method constitutes a fast and accurate method for a range of different approximation qualities. Moreover, in the third plot, one can observe that the confidence informations are reliable already for small approximation orders since it is based on the consistent covariance intersection method. This experiment is available in a notebook[9] on github.

A.7.2 Real World Data

In this section, we provide more details about the experiments with real world data as summarized in Section 4. The used datasets in this paper, as summarized in Table 3a, are from public data repositories and can be downloaded as shown in a jupyter notebook [10] on github. In particular, it shows where and how to download the raw datasets, which variables are used as input and the target variables, where we followed the remarks on the repositories or other authors previously worked with these datasets. Finally, the preprocessing of the data is shown, where we standardized all variables to mean zero and standard deviation of one (for *elecdemand* see details below). We use $N = min(0.9N_{tot}, 1000)$ data sample for training, the rest for testing; except for *kin* and *elecdemand* we run experiments with $N_{test} = 3000$ and $N_{test} = 15288$ such that we could also run full GP a standard Laptop. For each dataset we fixed the number J of experts (given in Table 3a) such that the partitions/mini-batches have a reasonable size (≈ 500).
For the deterministic SGP we used $M = 100$ and for the stochastic SGP $M \in \{500, 1000\}$ inducing points (more results are provided in Appendix F).

[9] https://github.com/manschuer/CPoE/experiments/syntheticData.ipynb
[10] https://github.com/manschuer/CPoE/experiments/download_data.ipynb

For our method CPoE we run the algorithm for $C \in \{1,2,3,4\}$ for the small and $C \in \{1,2,3\}$ for the large datasets with always $\gamma = 1$. For the stochastic versions we used learning rates $\delta = 0.03$ for the dataset *kin2* and $\delta = 0.01$ for the remaining for all methods. The maximum number of epochs is set to 15 together with a relative stopping criteria of $1e^{-2}$. We use a SE-kernel with a different lengthscale per dimension and initialized all hyperparameters to 1, and the global inducing point to a random subset of the data. These experiments are provided at github. [11] [12]

A.7.3 Application

This section contains additional details to the application described in Section 4.4, where our method is applied to the *elecdemand* time series [37], which contains the half-hourly measured electricity demand together with the corresponding temperature and the variable whether it is a working day for 1 year. In particular, the preprocessed dataset contains the standardized electricity demand (mean=0, sd=1) as the response variable y, the normalized time as the first variable $X_1 \in [0,1]$, the standardized temperature and indicators as X_2 and X_3, respectively. The data is depicted in the first two plots in Fig. 10, where we shifted the first and third variable in the second plot for the sake of clarity. We removed the last day resulting in 364 days = 52 weeks = 13 "months" consisting of 4 weeks. In each of the 13 "months", we used the first 3 weeks for training and the last week for testing the out-of-sample accuracy. In order that it is possible to run full GP as comparison, we only used every 6th sample (corresponding to a measurement every 3h) of the training weeks for the actual training and the remaining for testing the in-sample accuracy. This gives $N = 2184$, $N_{IN} = 10920$ and $N_{OUT} = 4368$ samples as depicted in the first plot in Fig. 10.
With our CPoE model it is straightforward to handle time series with covariates, as opposed to other time series methods [33–36]. The kernel $k_{\boldsymbol{\theta}}$ depends on several hyperparameters $\boldsymbol{\theta}$, for which we use the parametrization in [33]. We assume a log-normal prior on $\boldsymbol{\theta}$ as described in Section A.3.3 in which the corresponding means and variances are taken from Table 1 in [33]. Similarly as in the previous section, we run full GP, SGP and PoEs and CPoE and optimized the hyperparameter deterministically using the MAP as objective function taking into account the priors. For SGP we used $M \in \{100, 200\}$ fixed inducing points, for PoEs and CPoE we used $J = 13$ partitions which are obtained by splitting the first variable into J blocks. For CPoE we used $C \in \{1,2,3,4\}$ and $\gamma = 1$. The results are provided in Table 3b which again shows very competitive performance also for a general kernel with priors on the hyperparameters.

[11] https://github.com/manschuer/CPoE/experiments/realData1.ipynb
[12] https://github.com/manschuer/CPoE/experiments/realData2.ipynb

Appendix B Useful properties

B.0.1 Inversion Lemma

Given invertible matrices $\boldsymbol{A} \in \mathbb{R}^{B\times B}$, $\boldsymbol{C} \in \mathbb{R}^{M\times M}$ and matrices $\boldsymbol{U} \in \mathbb{R}^{B\times M}$, $\boldsymbol{V} \in \mathbb{R}^{M\times B}$, it holds

$$\left(\boldsymbol{A}+\boldsymbol{UCV}\right)^{-1} = \boldsymbol{A}^{-1} - \boldsymbol{A}^{-1}\boldsymbol{U}\left(\boldsymbol{C}^{-1}+\boldsymbol{VA}^{-1}\boldsymbol{U}\right)^{-1}\boldsymbol{VA}^{-1}. \tag{B2}$$

B.0.2 Determinant Lemma

Given invertible matrices $\boldsymbol{A} \in \mathbb{R}^{B\times B}$, $\boldsymbol{C} \in \mathbb{R}^{M\times M}$ and matrices $\boldsymbol{U} \in \mathbb{R}^{B\times M}$, $\boldsymbol{V} \in \mathbb{R}^{M\times B}$, it holds

$$|\boldsymbol{A}+\boldsymbol{UCV}| = |\boldsymbol{C}^{-1}+\boldsymbol{VA}^{-1}\boldsymbol{U}||\boldsymbol{C}||\boldsymbol{A}|. \tag{B3}$$

B.0.3 Block Inversion

Given an invertible, symmetric block matrix

$$\boldsymbol{M} = \begin{bmatrix} \boldsymbol{A} & \boldsymbol{B} \\ \boldsymbol{B}^T & \boldsymbol{D} \end{bmatrix},$$

the inverse can be computed as

$$\boldsymbol{M}^{-1} = \begin{bmatrix} \boldsymbol{A}^{-1}+\boldsymbol{A}^{-1}\boldsymbol{BZ}^{-1}\boldsymbol{B}^T\boldsymbol{A}^{-1} & -\boldsymbol{A}^{-1}\boldsymbol{BZ}^{-1} \\ -\boldsymbol{Z}^{-1}\boldsymbol{B}^T\boldsymbol{A}^{-1} & \boldsymbol{Z}^{-1} \end{bmatrix} \tag{B4}$$

with $\boldsymbol{Z} = \boldsymbol{D} - \boldsymbol{B}^T\boldsymbol{A}^{-1}\boldsymbol{B}$.

B.0.4 Block Determinant

Given an invertible, symmetric block matrix

$$\boldsymbol{M} = \begin{bmatrix} \boldsymbol{A} & \boldsymbol{B} \\ \boldsymbol{B}^T & \boldsymbol{D} \end{bmatrix},$$

the determinant can be computed as

$$|\boldsymbol{M}| = |\boldsymbol{A}|\;|\boldsymbol{D}-\boldsymbol{B}^T\boldsymbol{A}^{-1}\boldsymbol{B}| = |\boldsymbol{D}|\;|\boldsymbol{A}-\boldsymbol{BD}^{-1}\boldsymbol{B}^T|. \tag{B5}$$

B.0.5 Conditional Gaussians

From the joint Gaussian $[\boldsymbol{a},\boldsymbol{b}]^T \sim \mathcal{N}\left(\boldsymbol{0},\boldsymbol{K}_{[\boldsymbol{A},\boldsymbol{B}][\boldsymbol{A},\boldsymbol{B}]}\right)$, the conditional can be computed as follows

$$\boldsymbol{a}|\boldsymbol{b} \sim \mathcal{N}\left(\boldsymbol{K}_{\boldsymbol{AB}}\boldsymbol{K}_{\boldsymbol{BB}}^{-1}\boldsymbol{b}, \boldsymbol{K}_{\boldsymbol{AA}} - \boldsymbol{K}_{\boldsymbol{AB}}\boldsymbol{K}_{\boldsymbol{BB}}^{-1}\boldsymbol{K}_{\boldsymbol{BA}}\right) = \mathcal{N}\left(\boldsymbol{H}_{AB}\boldsymbol{b}, \boldsymbol{V}_{\boldsymbol{AA}}^{\boldsymbol{B}}\right) \tag{B6}$$

B.0.6 Marginalization/Integration

Given the densities $p(\boldsymbol{a}) = \mathcal{N}(\boldsymbol{\mu}, \boldsymbol{\Sigma})$ and $p(\boldsymbol{b}|\boldsymbol{a}) = \mathcal{N}(\boldsymbol{F}\boldsymbol{a} + \boldsymbol{v}, \boldsymbol{Q})$, then

$$p(\boldsymbol{b}) = \int p(\boldsymbol{a}, \boldsymbol{b})\, \mathrm{d}\boldsymbol{a} = \int p(\boldsymbol{b}|\boldsymbol{a})\, p(\boldsymbol{a})\, \mathrm{d}\boldsymbol{a} = \mathcal{N}\left(\boldsymbol{F}\boldsymbol{\mu} + \boldsymbol{v}, \boldsymbol{F}\boldsymbol{\Sigma}\boldsymbol{F}^T + \boldsymbol{Q}\right) \quad \text{(B7)}$$

B.0.7 Gaussian & Bayes

Given the densities $p(\boldsymbol{a}) = \mathcal{N}(\boldsymbol{\mu}, \boldsymbol{\Sigma})$ and $p(\boldsymbol{b}|\boldsymbol{a}) = \mathcal{N}(\boldsymbol{F}\boldsymbol{a} + \boldsymbol{v}, \boldsymbol{Q})$, applying Bayes' formula yields

$$p(\boldsymbol{a}|\boldsymbol{b}) = \mathcal{N}\left(\boldsymbol{P}\left(\boldsymbol{F}^T\boldsymbol{Q}^{-1}(\boldsymbol{b} - \boldsymbol{v}) + \boldsymbol{\Sigma}^{-1}\boldsymbol{\mu}\right), \boldsymbol{P}\right), \quad \text{(B8)}$$

with $\boldsymbol{P} = \left(\boldsymbol{\Sigma}^{-1} + \boldsymbol{F}^T\boldsymbol{Q}^{-1}\boldsymbol{F}\right)^{-1}$.

B.0.8 Product of Gaussians

Assume J Gaussians $p_J(\boldsymbol{x}) = \mathcal{N}\left(\boldsymbol{x}|\boldsymbol{\mu}_j, \boldsymbol{\Sigma}_j\right)$ and $a_j \in \mathbb{R}$. Then the product can be written as

$$\prod_{j=1}^{J} p_j(\boldsymbol{x})^{a_j} = \mathcal{N}(\boldsymbol{x}|\boldsymbol{\mu}, \boldsymbol{\Sigma}) \quad \text{(B9)}$$

with

$$\boldsymbol{\Sigma} = \left(\sum_{j=1}^{J} a_j \boldsymbol{\Sigma}_j^{-1}\right)^{-1} \quad \text{and} \quad \boldsymbol{\mu} = \boldsymbol{\Sigma}\left(\sum_{j=1}^{J} a_j \boldsymbol{\Sigma}_j^{-1}\boldsymbol{\mu}_j\right)$$

as long as $\boldsymbol{\Sigma}$ positive-semi-definite (if $a_j \equiv 1$ then always the case).

B.0.9 Entropy of Gaussian

The *Entropy* H of $p(\boldsymbol{x}) = \mathcal{N}(\boldsymbol{x}|\boldsymbol{\mu}, \boldsymbol{\Sigma})$ with $|\boldsymbol{x}| = B$ is defined as

$$H[\boldsymbol{x}] = H[p(\boldsymbol{x})] = \frac{1}{2}\left(\log|\boldsymbol{\Sigma}| + B(1 + \log 2\pi)\right), \quad \text{(B10)}$$

where we use *log* as the natural logarithm and thus the entropy is measured in nats (natural units).

B.0.10 Kullback-Leibler-Divergence (KL)

The KL between $p_0(\boldsymbol{x})$ and $p_1(\boldsymbol{x})$ is defined as

$$KL[p_0(\boldsymbol{x}) \mid p_1(\boldsymbol{x})] = \int p_0(\boldsymbol{x}) \log \frac{p_0(\boldsymbol{x})}{p_1(\boldsymbol{x})}\, \mathrm{d}\boldsymbol{x}. \quad \text{(B11)}$$

B.0.11 KL between 2 Gaussians

The *Kullback-Leibler-Divergence (KL)* between $p_0(\boldsymbol{x}) = \mathcal{N}(\boldsymbol{\mu}_0, \boldsymbol{\Sigma}_0)$ and $p_1(\boldsymbol{x}) = \mathcal{N}(\boldsymbol{\mu}_1, \boldsymbol{\Sigma}_1)$ with $|\boldsymbol{x}| = B$ can be computed by

$$\begin{aligned} KL[p_0(\boldsymbol{x}) \mid p_1(\boldsymbol{x})] = \frac{1}{2} \bigg(tr(\boldsymbol{\Sigma}_1^{-1}\boldsymbol{\Sigma}_0) - B \\ + (\boldsymbol{\mu}_1 - \boldsymbol{\mu}_0)^T \boldsymbol{\Sigma}_1^{-1} (\boldsymbol{\mu}_1 - \boldsymbol{\mu}_0) + \log \frac{|\boldsymbol{\Sigma}_1|}{|\boldsymbol{\Sigma}_0|} \bigg). \end{aligned} \tag{B12}$$

B.0.12 Difference in KL of Gaussian with Zero Mean

The difference in KL between $p_1(\boldsymbol{x}) = \mathcal{N}(\mathbf{0}, \boldsymbol{\Sigma}_1)$ and $p_2(\boldsymbol{x}) = \mathcal{N}(\mathbf{0}, \boldsymbol{\Sigma}_2)$ with same base distribution $p_0(\boldsymbol{x}) = \mathcal{N}(\mathbf{0}, \boldsymbol{\Sigma}_0)$ can be computed by

$$KL[p_0(\boldsymbol{x}) \mid p_1(\boldsymbol{x})] - KL[p_0(\boldsymbol{x}) \mid p_2(\boldsymbol{x})] = \frac{1}{2} \left(tr((\boldsymbol{\Sigma}_1^{-1} - \boldsymbol{\Sigma}_2^{-1})\boldsymbol{\Sigma}_0) + \log \frac{|\boldsymbol{\Sigma}_1|}{|\boldsymbol{\Sigma}_2|} \right). \tag{B13}$$

B.0.13 General Difference in KL

Let $1 \le C < J$ and $0 < \gamma \le 1$ be fixed. For any $C_2 \in \{C, \dots, J\}$ we define the difference in KL, denoted as $\mathbb{D}_{(C,C_2)}[\boldsymbol{x}]$, between the true distribution of $\boldsymbol{x}$ and two different approximate distributions, i.e.

$$\mathbb{D}_{(C,C_2)}[\boldsymbol{x}] = KL[p(\boldsymbol{x}) \,||\, q_{c,\gamma}(\boldsymbol{x})] - KL[p(\boldsymbol{x}) \,||\, q_{c_2,\gamma}(\boldsymbol{x})] = \mathbb{E}_{p(\boldsymbol{x})} \left[\log \frac{q_{c_2,\gamma}(\boldsymbol{x})}{q_{c,\gamma}(\boldsymbol{x})} \right] \tag{B14}$$

using the definition of KL (B11). Similarly, we define the the difference in KL, denotes as $\mathbb{D}_{(C,C_2)}[\boldsymbol{x}|\boldsymbol{y}]$, of a conditional distribution $\boldsymbol{x}|\boldsymbol{y}$ to be

$$\begin{aligned} KL[p(\boldsymbol{x}|\boldsymbol{y}) \,||\, q_{c,\gamma}(\boldsymbol{x}|\boldsymbol{y})] - KL[p(\boldsymbol{x}|\boldsymbol{y}) \,||\, q_{c_2,\gamma}(\boldsymbol{x}|\boldsymbol{y})] \\ = \mathbb{E}_{p(\boldsymbol{y})} \left[\mathbb{E}_{p(\boldsymbol{x}|\boldsymbol{y})} \left[\log \frac{q_{c_2,\gamma}(\boldsymbol{x}|\boldsymbol{y})}{q_{c,\gamma}(\boldsymbol{x}|\boldsymbol{y})} \right] \right]. \end{aligned} \tag{B15}$$

which follows from the the definition of KL (B11).

Appendix C Proofs and Additional Results

C.1 Additional Results

Proposition 9 (Marginal Likelihood; Proof 13) *The marginal likelihood is*

$$q_{C,\gamma}(\boldsymbol{y}|\boldsymbol{\theta}) = q_{C,\gamma}(\boldsymbol{y}) = \int q_{C,\gamma}(\boldsymbol{y}, \boldsymbol{a})\, \mathrm{d}\boldsymbol{a} = \mathcal{N}(\mathbf{0}, \boldsymbol{P})$$

with $\boldsymbol{P} = \boldsymbol{H}\boldsymbol{S}^{-1}\boldsymbol{H}^T + \boldsymbol{V} \in \mathbb{R}^{N \times N}$ *where all dependencies on* $\boldsymbol{\theta}$ *of the matrices are omitted.*

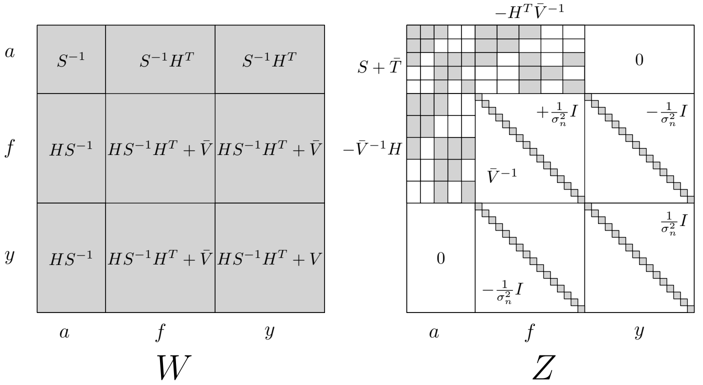

Fig. C7: Covariance $\boldsymbol{W}$ and precision $\boldsymbol{Z} = \boldsymbol{W}^{-1}$ of joint prior approximation $q_{c,\gamma}(\boldsymbol{a}, \boldsymbol{f}, \boldsymbol{y} = \mathcal{N}(\boldsymbol{0}, \boldsymbol{W})$ of CPoE model. Compare Proof 19.

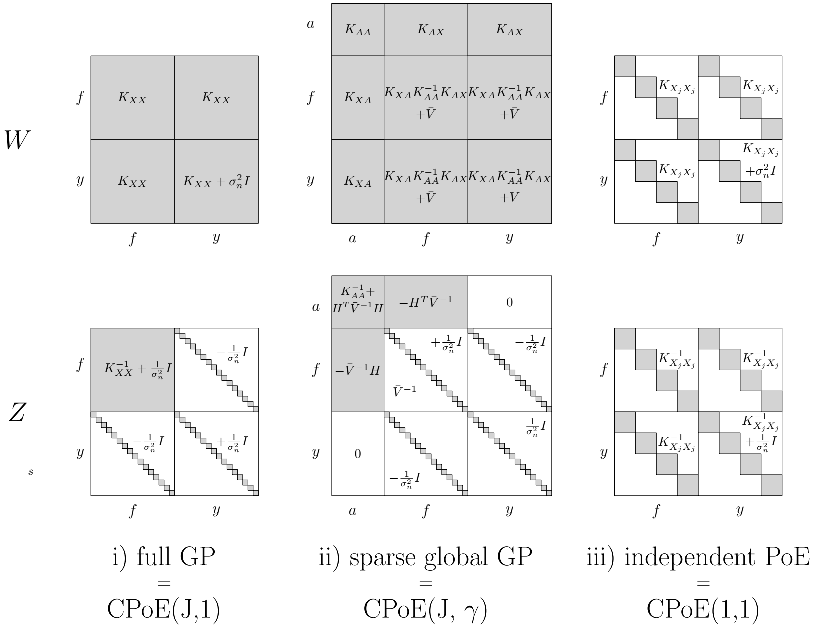

Fig. C8: Covariance $\boldsymbol{W}$ and precision $\boldsymbol{Z} = \boldsymbol{W}^{-1}$ of joint prior of different GP models. Compare Figure C7 for the corresponding covariance and precision matrices for CPoE model. Note that we used $\boldsymbol{H} = \boldsymbol{K_{XA}}\boldsymbol{K_{AA}^{-1}}$ and $\bar{\boldsymbol{V}}$ the same as in the local CPoE.

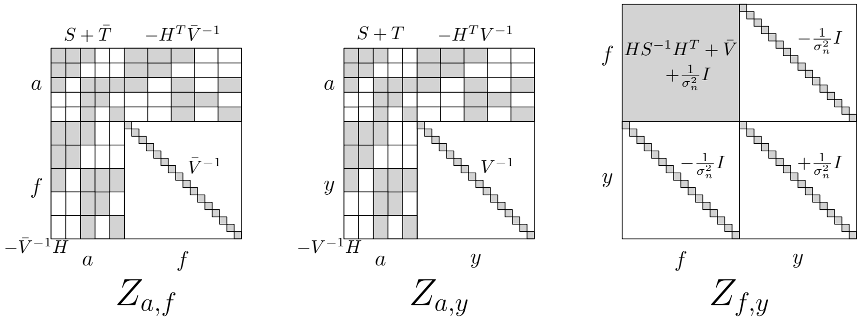

Fig. C9: Marginalized precision corresponding to $q_{c,\gamma}(\boldsymbol{a},\boldsymbol{f}) = \mathcal{N}\left(\mathbf{0}, \boldsymbol{Z}_{\boldsymbol{a},\boldsymbol{f}}^{-1}\right)$, $q_{c,\gamma}(\boldsymbol{a},\boldsymbol{y}) = \mathcal{N}\left(\mathbf{0}, \boldsymbol{Z}_{\boldsymbol{a},\boldsymbol{y}}^{-1}\right)$ and $q_{c,\gamma}(\boldsymbol{f},\boldsymbol{a}) = \mathcal{N}\left(\mathbf{0}, \boldsymbol{Z}_{\boldsymbol{f},\boldsymbol{y}}^{-1}\right)$, respectively. Thereby we used the notation $\boldsymbol{V} = \bar{\boldsymbol{V}} + \sigma_n^2\mathbb{I}$, $\bar{\boldsymbol{T}} = \boldsymbol{H}^T\bar{\boldsymbol{V}}^{-1}\boldsymbol{H}$ and $\boldsymbol{T} = \boldsymbol{H}^T\boldsymbol{V}^{-1}\boldsymbol{H}$. Note that the corresponding dense covariance matrices are directly obtained from $\boldsymbol{W}$ in Fig. C7 by selecting the corresponding entries.

Proposition 10 (Prior Approximation II; Proof 15) *Alternatively to Proposition 2, the prior approximation* $q(\boldsymbol{a}) = \mathcal{N}\left(\boldsymbol{a}|\mathbf{0}, \boldsymbol{S}^{-1}\right)$ *can be equivalently written as*

$$q(\boldsymbol{a}) = \prod_{j=1}^{J} p\left(\boldsymbol{a}_j|\boldsymbol{a}_{\boldsymbol{\pi}(j)}\right) = \prod_{j=1}^{J} \mathcal{N}\left(\boldsymbol{a}_{\boldsymbol{\pi}^+(j)}|\mathbf{0}, \boldsymbol{S}_{(j)}^{-1}\right)$$

with $\boldsymbol{S}_{(j)} = \tilde{\boldsymbol{F}}_j^T\boldsymbol{Q}_j^{-1}\tilde{\boldsymbol{F}}_j$, $\tilde{\boldsymbol{F}}_j = \left[-\boldsymbol{F}_j \ \mathbb{I}\right]$ *and* $\boldsymbol{\pi}^+(j) = \boldsymbol{\pi}(j) \cup j$. *Further, the prior precision matrix can also be written as*

$$\boldsymbol{S} = \sum_{j=1}^{J} \overline{\boldsymbol{S}}_{(j)}$$

where $\overline{\boldsymbol{S}}_{(j)} \in \mathbb{R}^{M\times M}$ *is the augmented matrix consisting of* $\boldsymbol{S}_{(j)} \in \mathbb{R}^{LC\times LC}$ *at the entries* $[\boldsymbol{\pi}^+(\boldsymbol{j}), \boldsymbol{\pi}^+(\boldsymbol{j})]$ *and* 0 *otherwise.*

Proposition 11 (Prior Approximation III; Proof 16) *Alternatively to Prop. 2 and Prop. 10 the prior approximation* $q(\boldsymbol{a})$ *can be equivalently written as*

$$q(\boldsymbol{a}) = \prod_{j=1}^{J} \frac{p\left(\boldsymbol{a}_j, \boldsymbol{a}_{\boldsymbol{\pi}(j)}\right)}{p\left(\boldsymbol{a}_{\boldsymbol{\pi}(j)}\right)} = \prod_{j=C}^{J} \frac{p\left(\boldsymbol{a}_{\boldsymbol{\pi}^+(j)}\right)}{p\left(\boldsymbol{a}_{\boldsymbol{\pi}(j)}\right)}$$

which is a Gaussian $\mathcal{N}\left(\boldsymbol{a}|\mathbf{0}, \boldsymbol{S}^{-1}\right)$ *with prior precision*

$$\boldsymbol{S} = \sum_{j=1}^{J} \overline{\boldsymbol{K}}_{\boldsymbol{A}_{\boldsymbol{\pi}^+(j)}\boldsymbol{A}_{\boldsymbol{\pi}^+(j)}}^{-1} - \overline{\boldsymbol{K}}_{\boldsymbol{A}_{\boldsymbol{\pi}(j)}\boldsymbol{A}_{\boldsymbol{\pi}(j)}}^{-1}$$

where $\overline{\boldsymbol{K}}_{\boldsymbol{A}_{\boldsymbol{\phi}}\boldsymbol{A}_{\boldsymbol{\phi}}}^{-1} \in \mathbb{R}^{M\times M}$ *is the augmented matrix consisting of* $\boldsymbol{K}_{\boldsymbol{A}_{\boldsymbol{\phi}}\boldsymbol{A}_{\boldsymbol{\phi}}}^{-1} \in \mathbb{R}^{T\times T}$ *at the entries* $[\boldsymbol{\phi}, \boldsymbol{\phi}]$ *and* 0 *otherwise with* $T = |\boldsymbol{\phi}|$.

Proposition 12 (Exact Diagonal of Prior; Proof 14) *The precision matrix* $\boldsymbol{S}_C$ *of the prior approximation* $q_C(\boldsymbol{a})$ *is exact on the diagonal, that is,*

$$tr(\boldsymbol{S}_C \boldsymbol{K}_{\boldsymbol{A}\boldsymbol{A}}) = JL$$

where JL *is the dimension of the matrices.*

Proposition 13 (Band-Diagonal Approximation) *In the* ***consecutive*** *case, i.e.* $\boldsymbol{\psi}(j) = \{j - C + 1, \ldots, j\}$*, the block-entries*

$$\boldsymbol{S}^{-1}_{[\boldsymbol{\psi}(j),\boldsymbol{\psi}(j)]} = \boldsymbol{K}_{\boldsymbol{A}\boldsymbol{A}[\boldsymbol{\psi}(j),\boldsymbol{\psi}(j)]},$$

are equal which means that the block-band-diagonals $-C+1, \ldots, 0, \ldots, C-1$ *of the both matrices are the same. For the case* $C = J$ *it holds* $\boldsymbol{S}^{-1} = \boldsymbol{K}_{\boldsymbol{A}\boldsymbol{A}}$.

Proposition 14 (Decreasing Prior Entropy; Proof 10) *For any predecessor structure* $\boldsymbol{\pi}_C$ *as in Def. 2, the entropy* H *of the approximate prior* $q_C(\boldsymbol{a})$ *is decreasing for* $C \to J$*, in particular*

$$H\left[q_1(\boldsymbol{a})\right] \geq \cdots \geq H\left[q_j(\boldsymbol{a})\right] \geq \cdots \geq H\left[q_J(\boldsymbol{a})\right]$$

where it holds $H\left[q_J(\boldsymbol{a})\right] = H\left[p(\boldsymbol{a})\right]$ *and*

$$H[q_j(\boldsymbol{a})] = \frac{1}{2}\log|\boldsymbol{Q}_C| + \frac{M}{2}(1 + \log 2\pi).$$

Similar results can be obtained for the joint prior $q_C(\boldsymbol{a}, \boldsymbol{f}, \boldsymbol{y})$.

From the last proposition we know that increasing the degree of correlation C add always more information to the prior. In particular, the prior of complete independent PoEs (i.e. $C = 1$) encodes the least of information since all correlations between the experts are missing, whereas the prior of full GP incorporates the most information since all correlations are modeled.

Proposition 15 (Prior Quality II) *The prior approximation quality improvement* $\mathbb{D}_{(C,T)}[\boldsymbol{a}]$ *in Prop. 6 can be equivalently written as*

$$\begin{aligned}\mathbb{D}_{(C,T)} &= \frac{1}{2}\log\frac{|\boldsymbol{S}_{C+T}|}{|\boldsymbol{S}_C|} = \frac{1}{2}\sum_{j=1}^{J}\log\frac{|\boldsymbol{Q}_{j|\boldsymbol{\pi}_j}|\ |\boldsymbol{Q}_{\boldsymbol{\phi}_j|\boldsymbol{\pi}_j}|}{|\boldsymbol{Q}_{j\cup\boldsymbol{\phi}_j|\boldsymbol{\pi}_j}|} \\ &= \frac{1}{2}\sum_{j=1}^{J}\log\frac{|\boldsymbol{K}_{\boldsymbol{A}_{j\cup\pi_j}\boldsymbol{A}_{j\cup\pi_j}}|\ |\boldsymbol{K}_{\boldsymbol{A}_{\phi_j\cup\pi_j}\boldsymbol{A}_{\phi_j\cup\pi_j}}|}{|\boldsymbol{K}_{\boldsymbol{A}_{j\cup\phi_j\cup\pi_j}\boldsymbol{A}_{j\cup\phi_j\cup\pi_j}}|\ |\boldsymbol{K}_{\boldsymbol{A}_{\pi_j}\boldsymbol{A}_{\pi_j}}|}\end{aligned}$$

where $\boldsymbol{\pi}_j = \boldsymbol{\pi}_C(j)$, $\boldsymbol{\phi}_j = \cup_{i=C+1}^{C+T}\boldsymbol{\phi}_i(j)$ *and* $\boldsymbol{Q}_{\varphi_1|\varphi_2} = \boldsymbol{K}_{\boldsymbol{A}_{\varphi_1}\boldsymbol{A}_{\varphi_1}} - \boldsymbol{K}_{\boldsymbol{A}_{\varphi_1}\boldsymbol{A}_{\varphi_2}}\boldsymbol{K}^{-1}_{\boldsymbol{A}_{\varphi_2}\boldsymbol{A}_{\varphi_2}}\boldsymbol{K}_{\boldsymbol{A}_{\varphi_2}\boldsymbol{A}_{\varphi_1}}$.

C.2 Proofs

Proof 1 (Proof of Prop. 1; Joint Distribution) . The matrices in the conditional distributions (6) and (7) in Prop. 1 can be obtained via Gaussian conditioning (B6) from the assumed joint densities

$$p\left(\boldsymbol{f}^i_j, \boldsymbol{a}_{\boldsymbol{\psi}(j)}\right) = \mathcal{N}\left(\boldsymbol{0}, \boldsymbol{K}_{[\boldsymbol{X}^i_j;\ \boldsymbol{A}_{\boldsymbol{\psi}(j)}][\boldsymbol{X}^i_j;\ \boldsymbol{A}_{\boldsymbol{\psi}(j)}]}\right);$$

$$p\left(\boldsymbol{a}_j, \boldsymbol{a}_{\boldsymbol{\pi}(j)}\right) = \mathcal{N}\left(\mathbf{0}, \boldsymbol{K}_{[\boldsymbol{A}_j, \boldsymbol{A}_{\boldsymbol{\pi}(j)}][\boldsymbol{A}_j, \boldsymbol{A}_{\boldsymbol{\pi}(j)}]}\right),$$

resulting in

$$\begin{aligned}
\boldsymbol{H}_j &= \boldsymbol{K}_{\boldsymbol{X}_j \boldsymbol{A}_{\boldsymbol{\psi}(j)}} \boldsymbol{K}^{-1}_{\boldsymbol{A}_{\boldsymbol{\psi}(j)} \boldsymbol{A}_{\boldsymbol{\psi}(j)}};\\
\overline{\boldsymbol{V}}_j &= Diag[\boldsymbol{K}_{\boldsymbol{X}_j \boldsymbol{X}_j} - \boldsymbol{K}_{\boldsymbol{X}_j \boldsymbol{A}_{\boldsymbol{\psi}(j)}} \boldsymbol{K}^{-1}_{\boldsymbol{A}_{\boldsymbol{\psi}(j)} \boldsymbol{A}_{\boldsymbol{\psi}(j)}} \boldsymbol{K}_{\boldsymbol{A}_{\boldsymbol{\psi}(j)} \boldsymbol{X}_j}];\\
\boldsymbol{F}_j &= \boldsymbol{K}_{\boldsymbol{A}_j \boldsymbol{A}_{\boldsymbol{\pi}(j)}} \boldsymbol{K}^{-1}_{\boldsymbol{A}_{\boldsymbol{\pi}(j)} \boldsymbol{A}_{\boldsymbol{\pi}(j)}};\\
\boldsymbol{Q}_j &= \boldsymbol{K}_{\boldsymbol{A}_j \boldsymbol{A}_j} - \boldsymbol{K}_{\boldsymbol{A}_j \boldsymbol{A}_{\boldsymbol{\pi}(j)}} \boldsymbol{K}^{-1}_{\boldsymbol{A}_{\boldsymbol{\pi}(j)} \boldsymbol{A}_{\boldsymbol{\pi}(j)}} \boldsymbol{K}_{\boldsymbol{A}_{\boldsymbol{\pi}(j)} \boldsymbol{A}_j}
\end{aligned}$$

with $\boldsymbol{F}_1 = \mathbf{0}$ and $\boldsymbol{Q}_1 = \boldsymbol{K}_{\boldsymbol{A}_1 \boldsymbol{A}_1}$.

Proof 2 (Proof used in Def. 4; Joint Distribution II) In the case $\gamma = 1$, thus $\boldsymbol{a}_j = \boldsymbol{f}_j$ and $\boldsymbol{a} = \boldsymbol{f}$, the joint distribution can be written as $q(\boldsymbol{f}, \boldsymbol{a}, \boldsymbol{y}) = q(\boldsymbol{f}, \boldsymbol{f}, \boldsymbol{y}) = q(\boldsymbol{f}, \boldsymbol{y})$ is

$$\begin{aligned}
q(\boldsymbol{f}, \boldsymbol{y}) &= \prod_{j=1}^{J} p(\boldsymbol{y}_j|\boldsymbol{f}_j)\, p\left(\boldsymbol{f}_j|\boldsymbol{a}_{\boldsymbol{\psi}(j)}\right) p\left(\boldsymbol{a}_j|\boldsymbol{a}_{\boldsymbol{\pi}(j)}\right)\\
&= \prod_{j=1}^{J} p(\boldsymbol{y}_j|\boldsymbol{f}_j)\, p\left(\boldsymbol{f}_j|\boldsymbol{f}_{\boldsymbol{\psi}(j)}\right) p\left(\boldsymbol{f}_j|\boldsymbol{f}_{\boldsymbol{\pi}(j)}\right)\\
&= \prod_{j=1}^{J} p(\boldsymbol{y}_j|\boldsymbol{f}_j) \frac{p\left(\boldsymbol{f}_j \boldsymbol{f}_{\boldsymbol{\psi}(j)}\right)}{p\left(\boldsymbol{f}_{\boldsymbol{\psi}(j)}\right)} p\left(\boldsymbol{f}_j|\boldsymbol{f}_{\boldsymbol{\pi}(j)}\right)\\
&= \prod_{j=1}^{J} p(\boldsymbol{y}_j|\boldsymbol{f}_j)\, p\left(\boldsymbol{f}_j|\boldsymbol{f}_{\boldsymbol{\pi}(j)}\right)
\end{aligned}$$

since

$$\frac{p\left(\boldsymbol{f}_j, \boldsymbol{f}_{\boldsymbol{\psi}(j)}\right)}{p\left(\boldsymbol{f}_{\boldsymbol{\psi}(j)}\right)} = \frac{p\left(\boldsymbol{f}_j, \boldsymbol{f}_j, \boldsymbol{f}_{\boldsymbol{\psi}(j)\setminus j}\right)}{p\left(\boldsymbol{f}_j, \boldsymbol{f}_{\boldsymbol{\psi}(j)\setminus j}\right)} = 1.$$

Proof 3 (Proof of Prop. 5; Equality to Full GP) **Full GP:** For $\gamma = 1$, the joint distribution of our model is formulated in Def. 4 and Proof 2. For $C = J$, we have

$$q_J(\boldsymbol{f}, \boldsymbol{y}) = \prod_{j=1}^{J} p(\boldsymbol{y}_j|\boldsymbol{f}_j)\, p\left(\boldsymbol{f}_j|\boldsymbol{f}_{\boldsymbol{\pi}_J(j)}\right),$$

where the predecessor set $\boldsymbol{\pi}_J(j)$ correspond to $\{1, \ldots, j-1\}$ and thus the conditional variables $\boldsymbol{f}_{\boldsymbol{\pi}(j)} = \boldsymbol{f}_{1:j-1}$. The posterior $q_J(\boldsymbol{f}|\boldsymbol{y})$ is proportional to the joint distribution $q_J(\boldsymbol{f}, \boldsymbol{y})$ (see Proof 12), thus we have

$$q_J(\boldsymbol{f}|\boldsymbol{y}) \propto \prod_{j=1}^{J} p(\boldsymbol{y}_j|\boldsymbol{f}_j)\, p(\boldsymbol{f}_j|\boldsymbol{f}_{1:j-1})$$

which is equal to the posterior distribution of full GP (1). Also the hyperparameter optimization is the same since the marginal likelihood $q_J(\boldsymbol{y})$ can be derived from the joint $q_C(\boldsymbol{f}, \boldsymbol{y})$ (see Proof 13). Further, in the prediction step, for $C = J$

we have $J_2 = C - J + 1 = 1$ predictive expert which is based on the full region $\psi(J) = \{1, \dots, J\}$. Therefore we conclude that the two models in considerations are the same.

Sparse global GP: Similarly, for $C = J$ but $\gamma < 1$, we have

$$\begin{aligned} q_J(\boldsymbol{f}, \boldsymbol{y}) &= \prod_{j=1}^{J} p\left(\boldsymbol{y}_j | \boldsymbol{f}_j\right) p\left(\boldsymbol{f}_j | \boldsymbol{a}_{\psi(j)}\right) p\left(\boldsymbol{a}_j | \boldsymbol{a}_{\boldsymbol{\pi}(j)}\right) \\ &= \prod_{j=1}^{J} p\left(\boldsymbol{y}_j | \boldsymbol{f}_j\right) p\left(\boldsymbol{f}_j | \boldsymbol{a}_{1:J}\right) p\left(\boldsymbol{a}_j | \boldsymbol{a}_{1:-j-1}\right) \\ &= \prod_{j=1}^{J} p\left(\boldsymbol{y}_j | \boldsymbol{f}_j\right) p\left(\boldsymbol{f}_j | \boldsymbol{a}\right) p\left(\boldsymbol{a}\right) \\ &= p\left(\boldsymbol{y} | \boldsymbol{f}\right) p\left(\boldsymbol{f} | \boldsymbol{a}\right) p\left(\boldsymbol{a}\right) \end{aligned}$$

so that the posterior correspond to that of sparse GP in (2.1). The prediction simplifies also to 1 predictive expert based on the full region. Also the marginal likelihood is the same for $C = J$ and could be adapted as illustrated in Section A.1.

Independent local GP: For $C = \gamma = 1$ we have

$$q_1(\boldsymbol{f}, \boldsymbol{y}) = \prod_{j=1}^{J} p\left(\boldsymbol{y}_j | \boldsymbol{f}_j\right) p\left(\boldsymbol{f}_j | \boldsymbol{f}_{\oslash}\right) = \prod_{j=1}^{J} p\left(\boldsymbol{y}_j | \boldsymbol{f}_j\right) p\left(\boldsymbol{f}_j\right)$$

which is equal to (4). Prediction and hyperparameters similar as above.

Proof 4 (Proof of Prop. 2; Prior Approximation) Here we prove the first part for the prior over $\boldsymbol{a}$, the second part is proved in Proof 5.

Using Prop. 10 (with Proof 15), the prior $q\left(\boldsymbol{a}\right)$ can be equivalently written as

$$\prod_{j=1}^{J} \mathcal{N}\left(\boldsymbol{a}_{\boldsymbol{\pi}^+(j)} | \boldsymbol{0}, \boldsymbol{S}_{(j)}^{-1}\right) \propto -\frac{1}{2} \boldsymbol{a}_{\boldsymbol{\pi}^+(j)}^T \tilde{\boldsymbol{F}}_j^T \boldsymbol{Q}_j^{-1} \tilde{\boldsymbol{F}}_j \boldsymbol{a}_{\boldsymbol{\pi}^+(j)}$$

with $\boldsymbol{S}_{(j)} = \tilde{\boldsymbol{F}}_j^T \boldsymbol{Q}_j^{-1} \tilde{\boldsymbol{F}}_j \in \mathbb{R}^{LC \times LC}$ and $\tilde{\boldsymbol{F}}_j = \left[-\boldsymbol{F}_j \ \mathbb{I}\right] \in \mathbb{R}^{L \times LC}$. This LC-dimensional Gaussian for $\boldsymbol{a}_{\boldsymbol{\pi}^+(j)}$ can be augmented to a M-dimensional Gaussian for $\boldsymbol{a}$ proportional to

$$-\frac{1}{2} \boldsymbol{a}^T \bar{\boldsymbol{F}}_j^T \bar{\boldsymbol{Q}}_j^{-1} \bar{\boldsymbol{F}}_j \boldsymbol{a} \quad \propto \quad \mathcal{N}\left(\boldsymbol{a} | \boldsymbol{0}, \left(\bar{\boldsymbol{F}}_j^T \bar{\boldsymbol{Q}}_j^{-1} \bar{\boldsymbol{F}}_j\right)^{-1}\right)$$

where $\bar{\boldsymbol{Q}}_j^{-1} \in \mathbb{R}^{M \times M}$ a zero matrix except $\boldsymbol{Q}_j^{-1} \in \mathbb{R}^{L \times L}$ at the entries $[\boldsymbol{\pi}^+(j), \boldsymbol{\pi}^+(j)]$. Further, the matrix $\bar{\boldsymbol{F}}_j \in \mathbb{R}^{M \times M}$ has one sparse row at j, that is,

$$\begin{bmatrix} 0 & 0 & 0 & 0 & 0 & 0 & 0 & 0 & 0 & 0 \\ \vdots & \vdots & \vdots & \vdots & \vdots & \vdots & \vdots & \vdots & \vdots & \vdots \\ 0 & \cdots & -\boldsymbol{F}_j^1 & 0 & -\boldsymbol{F}_j^i & \cdots & -\boldsymbol{F}_j^{I_j} & \mathbb{I} & \cdots & 0 \\ \vdots & \vdots & \vdots & \vdots & \vdots & \vdots & \vdots & \vdots & \vdots & \vdots \\ 0 & 0 & 0 & 0 & 0 & 0 & 0 & 0 & 0 & 0 \end{bmatrix}$$

where $\boldsymbol{F}_j^i \in \mathbb{R}^{L\times L}$ is the ith part of $\boldsymbol{F}_j \in \mathbb{R}^{L\times L(C-1)}$ which correspond to the contribution of the ith predecessor $\boldsymbol{\pi}^i(j)$.
By using the property in (B9), the original product $q(\boldsymbol{a})$ is then

$$\prod_{j=1}^{J} \mathcal{N}\left(\boldsymbol{a}|\boldsymbol{0}, \left(\bar{\boldsymbol{F}}_j^T \bar{\boldsymbol{Q}}_j^{-1} \bar{\boldsymbol{F}}_j\right)^{-1}\right) = \mathcal{N}\left(\boldsymbol{a}|\boldsymbol{0}, \left(\sum_{j=1}^{J} \bar{\boldsymbol{F}}_j^T \bar{\boldsymbol{Q}}_j^{-1} \bar{\boldsymbol{F}}_j\right)^{-1}\right)$$

$$= \mathcal{N}\left(\boldsymbol{a}|\boldsymbol{0}, \left(\boldsymbol{F}^T \boldsymbol{Q}^{-1} \boldsymbol{F}\right)^{-1}\right) = \mathcal{N}\left(\boldsymbol{a}|\boldsymbol{0}, \boldsymbol{S}^{-1}\right)$$

with $\boldsymbol{Q}^{-1} = Diag[\boldsymbol{Q}_1^{-1}, \ldots, \boldsymbol{Q}_J^{-1}]$ and $\boldsymbol{F}$ correspond then to the matrix depicted in Fig. 5. Note that $\boldsymbol{S}$ is positive definite since $\boldsymbol{Q}^{-1}$ positive definite because each $\boldsymbol{Q}_j^{-1}$ is positive definite which concludes the proof.

Proof 5 ((Sub)proof of Prop. 2 (Projection Approximation)) The projection $q\left(\boldsymbol{f}|\boldsymbol{a}\right) = q_C(\boldsymbol{f}|\boldsymbol{a})$ is

$$q_C(\boldsymbol{f}|\boldsymbol{a}) = \prod_{j=1}^{J} p\left(\boldsymbol{f}_j|\boldsymbol{a}_{\boldsymbol{\psi}(j)}\right) = \prod_{j=1}^{J} \mathcal{N}\left(\boldsymbol{f}_j|\boldsymbol{H}_j\boldsymbol{a}_{\boldsymbol{\psi}(j)}, \overline{\boldsymbol{V}}_j\right),$$

where $\boldsymbol{H}_j \in \mathbb{R}^{B\times LC}$ and $\overline{\boldsymbol{V}}_j \in \mathbb{R}^{B\times B}$. The log of this density in $\boldsymbol{f}_j \in \mathbb{R}^B$ is proportional to

$$\propto -\frac{1}{2}(\boldsymbol{f}_j - \boldsymbol{H}_j\boldsymbol{a}_{\boldsymbol{\psi}(j)})^T \overline{\boldsymbol{V}}_j^{-1}(\boldsymbol{f}_j - \boldsymbol{H}_j\boldsymbol{a}_{\boldsymbol{\psi}(j)})$$

which can be equivalently written as

$$-\frac{1}{2}(\mathbb{I}_j\boldsymbol{f} - \bar{\boldsymbol{H}}_j\boldsymbol{a})^T \bar{\overline{\boldsymbol{V}}}_j^{-1}(\mathbb{I}_j\boldsymbol{f} - \bar{\boldsymbol{H}}_j\boldsymbol{a})$$

with $\bar{\overline{\boldsymbol{V}}}_j \in \mathbb{R}^{M\times M}$ with $\overline{\boldsymbol{V}}_j$ at $[\boldsymbol{\psi}(j), \boldsymbol{\psi}(j)]$ and $\bar{\boldsymbol{H}}_j \in \mathbb{R}^{BJ\times M}$ the following matrix

$$\begin{bmatrix} 0 & 0 & 0 & 0 & 0 & 0 & 0 & 0 & 0 & 0 \\ \vdots & \vdots & \vdots & \vdots & \vdots & \vdots & \vdots & \vdots & \vdots & \vdots \\ 0 & \cdots & \boldsymbol{H}_j^1 & 0 & \boldsymbol{H}_j^i & \cdots & \boldsymbol{H}_j^{C-1} & \boldsymbol{H}_j^C & \cdots & 0 \\ \vdots & \vdots & \vdots & \vdots & \vdots & \vdots & \vdots & \vdots & \vdots & \vdots \\ 0 & 0 & 0 & 0 & 0 & 0 & 0 & 0 & 0 & 0 \end{bmatrix}$$

where jth row not empty with $\boldsymbol{H}_j^i \in \mathbb{R}^{B\times L}$ the ith entry in $\boldsymbol{H}_j$ which correspond to to $\boldsymbol{\psi}^i(j)$. Further, $\mathbb{I}_j \in \mathbb{R}^{BJ\times BJ}$ a zero matrix with $\mathbb{I} \in \mathbb{R}^{B\times B}$ at $[j,j]$. For the original product of the projections

$$q_C(\boldsymbol{f}|\boldsymbol{a}) = \prod_{j=1}^{J} \mathcal{N}\left(\boldsymbol{0}|\mathbb{I}_j^T\boldsymbol{f} - \bar{\boldsymbol{H}}_j\boldsymbol{a}, \bar{\overline{\boldsymbol{V}}}_j\right),$$

using the product rule of Gaussians in (B9), we obtain

$$\mathcal{N}\left(\boldsymbol{0}|\overline{\boldsymbol{V}}\sum_{j}^{J}\bar{\overline{\boldsymbol{V}}}_j^{-1}\left(\mathbb{I}_j\boldsymbol{f} - \bar{\boldsymbol{H}}_j\boldsymbol{a}\right), \left(\sum_{j}^{J}\bar{\overline{\boldsymbol{V}}}_j^{-1}\right)^{-1}\right)$$

$$= \mathcal{N}\left(\mathbf{0}|\overline{\boldsymbol{V}}\overline{\boldsymbol{V}}^{-1}\sum_j^J\left(\mathbb{I}_j\boldsymbol{f}-\bar{\boldsymbol{H}}_j\boldsymbol{a}\right),\overline{\boldsymbol{V}}\right) = \mathcal{N}\left(\mathbf{0}|\sum_j^J\left(\mathbb{I}_j\right)\boldsymbol{f}-\sum_j^J\left(\bar{\boldsymbol{H}}_j\right)\boldsymbol{a},\overline{\boldsymbol{V}}\right)$$
$$= \mathcal{N}\left(\mathbf{0}|\mathbb{I}\boldsymbol{f}-\boldsymbol{H}\boldsymbol{a},\overline{\boldsymbol{V}}\right) = \mathcal{N}\left(\boldsymbol{f}|\boldsymbol{H}\boldsymbol{a},\overline{\boldsymbol{V}}\right).$$

Since $\overline{\boldsymbol{V}}$ positive definite this concludes the statement.

Proof 6 (Proof of Prop. 6; Decreasing Prior KL) We first show the decomposition

$$\mathbb{D}_{(C,T)}[\boldsymbol{f},\boldsymbol{a},\boldsymbol{y}] = \mathbb{D}_{(C,T)}[\boldsymbol{a}] + \mathbb{D}_{(C,T)}[\boldsymbol{f}|\boldsymbol{a}] + \mathbb{D}_{(C,T)}[\boldsymbol{y}|\boldsymbol{f}].$$

Starting with the definition in Def. B14 we get

$$\begin{aligned}
&\mathbb{D}_{(C,T)}[\boldsymbol{f},\boldsymbol{a},\boldsymbol{y}]\\
&= \mathbb{E}_{p(\boldsymbol{f},\boldsymbol{a},\boldsymbol{y})}\left[\log\frac{q_{C+T}(\boldsymbol{f},\boldsymbol{a},\boldsymbol{y})}{q_C(\boldsymbol{f},\boldsymbol{a},\boldsymbol{y})}\right]\\
&= \mathbb{E}_{p(\boldsymbol{y}|\boldsymbol{f})p(\boldsymbol{f}|\boldsymbol{a})p(\boldsymbol{a})}\left[\log\frac{q_{C+T}(\boldsymbol{y}|\boldsymbol{f})q_{C+T}(\boldsymbol{f}|\boldsymbol{a})q_{C+T}(\boldsymbol{a})}{q_C(\boldsymbol{y}|\boldsymbol{f})q_C(\boldsymbol{f}|\boldsymbol{a})q_C(\boldsymbol{a})}\right]\\
&= \int p(\boldsymbol{y}|\boldsymbol{f})p(\boldsymbol{f}|\boldsymbol{a})p(\boldsymbol{a})\log\frac{q_{C+T}(\boldsymbol{y}|\boldsymbol{f})q_{C+T}(\boldsymbol{f}|\boldsymbol{a})q_{C+T}(\boldsymbol{a})}{q_C(\boldsymbol{y}|\boldsymbol{f})q_C(\boldsymbol{f}|\boldsymbol{a})q_C(\boldsymbol{a})}\,\mathrm{d}\boldsymbol{a}\,\mathrm{d}\boldsymbol{f}\,\mathrm{d}\boldsymbol{y}\\
&= \int p(\boldsymbol{a})\left(\int p(\boldsymbol{f}|\boldsymbol{a})\left[\int p(\boldsymbol{y}|\boldsymbol{f})\log\frac{q_{C+T}(\boldsymbol{y}|\boldsymbol{f})}{q_C(\boldsymbol{y}|\boldsymbol{f})}\,\mathrm{d}\boldsymbol{y}\,\cdots\right.\right.\\
&\qquad\left.\left. +\ \log\frac{q_{C+T}(\boldsymbol{f}|\boldsymbol{a})}{q_C(\boldsymbol{f}|\boldsymbol{a})}\right]\mathrm{d}\boldsymbol{f} + \log\frac{q_{C+T}(\boldsymbol{a})}{q_C(\boldsymbol{a})}\right)\mathrm{d}\boldsymbol{a}\\
&= \int p(\boldsymbol{a})\log\frac{q_{C+T}(\boldsymbol{a})}{q_C(\boldsymbol{a})}\,\mathrm{d}\boldsymbol{a} + \int p(\boldsymbol{a})\int p(\boldsymbol{f}|\boldsymbol{a})\log\frac{q_{C+T}(\boldsymbol{f}|\boldsymbol{a})}{q_C(\boldsymbol{f}|\boldsymbol{a})}\,\mathrm{d}\boldsymbol{f}\\
&\qquad + \int p(\boldsymbol{f})\int p(\boldsymbol{y}|\boldsymbol{f})\log\frac{q_{C+T}(\boldsymbol{y}|\boldsymbol{f})}{q_C(\boldsymbol{y}|\boldsymbol{f})}\,\mathrm{d}\boldsymbol{y}\,\mathrm{d}\boldsymbol{f}\\
&= \mathbb{E}_{p(\boldsymbol{a})}\left[\log\frac{q_{C+T}(\boldsymbol{a})}{q_C(\boldsymbol{a})}\right] + \mathbb{E}_{p(\boldsymbol{a})}\left[\mathbb{E}_{p(\boldsymbol{f}|\boldsymbol{a})}\left[\log\frac{q_{C+T}(\boldsymbol{f}|\boldsymbol{a})}{q_C(\boldsymbol{f}|\boldsymbol{a})}\right]\right]\\
&\qquad + \mathbb{E}_{p(\boldsymbol{f})}\left[\mathbb{E}_{p(\boldsymbol{y}|\boldsymbol{f})}\left[\log\frac{q_{C+T}(\boldsymbol{y}|\boldsymbol{f})}{q_C(\boldsymbol{y}|\boldsymbol{f})}\right]\right]\\
&= \mathbb{D}_{(C,T)}[\boldsymbol{a}] + \mathbb{D}_{(C,T)}[\boldsymbol{f}|\boldsymbol{a}] + \mathbb{D}_{(C,T)}[\boldsymbol{y}|\boldsymbol{f}],
\end{aligned}$$

where we used the definitions in Def. B14. We also immediately see that

$$\mathbb{D}_{(C,T)}[\boldsymbol{y}|\boldsymbol{f}] = 0$$

since $q_C(\boldsymbol{y}|\boldsymbol{f}) = q_{C+T}(\boldsymbol{y}|\boldsymbol{f}) = p(\boldsymbol{y}|\boldsymbol{f})$ is exact. The proofs for $\mathbb{D}_{(C,T)}[\boldsymbol{a}] \geq 0$ and $\mathbb{D}_{(C,T)}[\boldsymbol{f}|\boldsymbol{a}] \geq 0$ are given in Proof 7, 8 and 8, respectively.

Proof 7 (Proof of Subproof I of Proof 6) We prove

$$\mathbb{D}_{(C,T)}[\boldsymbol{a}] = \mathbb{E}_{p(\boldsymbol{a})}\left[\log\frac{q_{C+T}(\boldsymbol{a})}{q_C(\boldsymbol{a})}\right] \geq 0.$$

We abbreviate $q_1(\boldsymbol{a}) = q_C(\boldsymbol{a})$ and $q_2(\boldsymbol{a}) = q_{C+T}(\boldsymbol{a})$. The difference $\mathbb{D}_{(C,T)}[\boldsymbol{a}]$ is

$$\int p(\boldsymbol{a})\log\frac{q_2(\boldsymbol{a})}{q_1(\boldsymbol{a})}\,\mathrm{d}\boldsymbol{a} = \int p(\boldsymbol{a})\log\frac{\prod_{j=1}^J p\left(\boldsymbol{a}_j|\boldsymbol{a}_{\boldsymbol{\pi}_2(j)}\right)}{\prod_{j=1}^J p\left(\boldsymbol{a}_j|\boldsymbol{a}_{\boldsymbol{\pi}_1(j)}\right)}\,\mathrm{d}\boldsymbol{a}$$

$$= \int p(\boldsymbol{a}) \sum_{j=1}^{J} \log \frac{p\left(\boldsymbol{a}_j | \boldsymbol{a}_{\boldsymbol{\pi}_2(j)}\right)}{p\left(\boldsymbol{a}_j | \boldsymbol{a}_{\boldsymbol{\pi}_1(j)}\right)} \,\mathrm{d}\boldsymbol{a} = \sum_{j=1}^{J} \int p(\boldsymbol{a}) \log \frac{p\left(\boldsymbol{a}_j | \boldsymbol{a}_{\boldsymbol{\pi}_2(j)}\right)}{p\left(\boldsymbol{a}_j | \boldsymbol{a}_{\boldsymbol{\pi}_1(j)}\right)} \,\mathrm{d}\boldsymbol{a}$$

We recall property (iii) in Def. 2, thus we have $\boldsymbol{\pi}_2(j) = \boldsymbol{\pi}_1(j) \cup \phi(j)$ where $\phi(j)$ is the additional predecessor of expert j in the model 2 compared to model 1. In the following, we abbreviate $\boldsymbol{\pi}_1(j) = \boldsymbol{\pi}(j)$ yielding

$$\begin{aligned}
&\sum_{j=1}^{J} \int p(\boldsymbol{a}) \log \frac{p\left(\boldsymbol{a}_j | \boldsymbol{a}_{\boldsymbol{\pi}(j)}, \boldsymbol{a}_{\boldsymbol{\phi}(j)}\right)}{p\left(\boldsymbol{a}_j | \boldsymbol{a}_{\boldsymbol{\pi}(j)}\right)} \,\mathrm{d}\boldsymbol{a} \\
&= \sum_{j=1}^{J} \int p(\boldsymbol{a}) \log \frac{p\left(\boldsymbol{a}_j | \boldsymbol{a}_{\boldsymbol{\pi}(j)}, \boldsymbol{a}_{\boldsymbol{\phi}(j)}\right) p\left(\boldsymbol{a}_{\boldsymbol{\phi}(j)} | \boldsymbol{a}_{\boldsymbol{\pi}(j)}\right)}{p\left(\boldsymbol{a}_j | \boldsymbol{a}_{\boldsymbol{\pi}(j)}\right) p\left(\boldsymbol{a}_{\boldsymbol{\phi}(j)} | \boldsymbol{a}_{\boldsymbol{\pi}(j)}\right)} \,\mathrm{d}\boldsymbol{a} \\
&= \sum_{j=1}^{J} \int p(\boldsymbol{a}) \log \frac{p\left(\boldsymbol{a}_j, \boldsymbol{a}_{\boldsymbol{\phi}(j)} | \boldsymbol{a}_{\boldsymbol{\pi}(j)}\right)}{p\left(\boldsymbol{a}_j | \boldsymbol{a}_{\boldsymbol{\pi}(j)}\right) p\left(\boldsymbol{a}_{\boldsymbol{\phi}(j)} | \boldsymbol{a}_{\boldsymbol{\pi}(j)}\right)} \,\mathrm{d}\boldsymbol{a} \\
&= \sum_{j=1}^{J} \int p(\tilde{\boldsymbol{a}}_j) \log \frac{p\left(\boldsymbol{a}_j, \boldsymbol{a}_{\boldsymbol{\phi}(j)} | \boldsymbol{a}_{\boldsymbol{\pi}(j)}\right)}{p\left(\boldsymbol{a}_j | \boldsymbol{a}_{\boldsymbol{\pi}(j)}\right) p\left(\boldsymbol{a}_{\boldsymbol{\phi}(j)} | \boldsymbol{a}_{\boldsymbol{\pi}(j)}\right)} \,\mathrm{d}\tilde{\boldsymbol{a}}_j \\
&= \sum_{j=1}^{J} I\left(\boldsymbol{a}_j, \boldsymbol{a}_{\boldsymbol{\phi}(j)} | \boldsymbol{a}_{\boldsymbol{\pi}(j)}\right) \geq 0
\end{aligned}$$

where $\tilde{\boldsymbol{a}}_j = \boldsymbol{a}_j \cup \boldsymbol{a}_{\boldsymbol{\phi}(j)} \cup \boldsymbol{a}_{\boldsymbol{\pi}(j)}$ and $I\left(\boldsymbol{a}_j, \boldsymbol{a}_{\boldsymbol{\phi}(j)} | \boldsymbol{a}_{\boldsymbol{\pi}(j)}\right)$ the conditional mutual information which is always positive [42, p. 30] and therefore concludes the first part of the proof.

Proof 8 (Subproof II of Proof 6) We prove

$$\mathbb{D}_{(C,T)}[\boldsymbol{f}|\boldsymbol{a}] = \mathbb{E}_{p(\boldsymbol{a})}\left[\mathbb{E}_{p(\boldsymbol{f}|\boldsymbol{a})}\left[\log \frac{q_{C+T}(\boldsymbol{f}|\boldsymbol{a})}{q_C(\boldsymbol{f}|\boldsymbol{a})}\right]\right] \geq 0.$$

We abbreviate $q_1(\boldsymbol{f}|\boldsymbol{a}) = q_C(\boldsymbol{f}|\boldsymbol{a})$ and $q_2(\boldsymbol{f}|\boldsymbol{a}) = q_{C+T}(\boldsymbol{f}|\boldsymbol{a})$. The difference $\mathbb{D}_{(C,T)}[\boldsymbol{f}|\boldsymbol{a}]$ is

$$\int p(\boldsymbol{a}) \int p(\boldsymbol{f}|\boldsymbol{a}) \log \frac{q_2(\boldsymbol{f}|\boldsymbol{a})}{q_1(\boldsymbol{f}|\boldsymbol{a})} \,\mathrm{d}\boldsymbol{f}\,\mathrm{d}\boldsymbol{a} = \int p(\boldsymbol{a}, \boldsymbol{f}) \log \frac{\prod_{j=1}^{J} p\left(\boldsymbol{f}_j | \boldsymbol{a}_{\boldsymbol{\psi}_2(j)}\right)}{\prod_{j=1}^{J} p\left(\boldsymbol{f}_j | \boldsymbol{a}_{\boldsymbol{\psi}_1(j)}\right)} \,\mathrm{d}\boldsymbol{f}\,\mathrm{d}\boldsymbol{a}$$

$$= \int p(\boldsymbol{a}, \boldsymbol{f}) \sum_{j=1}^{J} \log \frac{p\left(\boldsymbol{f}_j | \boldsymbol{a}_{\boldsymbol{\psi}_2(j)}\right)}{p\left(\boldsymbol{f}_j | \boldsymbol{a}_{\boldsymbol{\psi}_1(j)}\right)} \,\mathrm{d}\boldsymbol{f}\,\mathrm{d}\boldsymbol{a} = \sum_{j=1}^{J} \int p(\boldsymbol{a}, \boldsymbol{f}) \log \frac{p\left(\boldsymbol{f}_j | \boldsymbol{a}_{\boldsymbol{\psi}_2(j)}\right)}{p\left(\boldsymbol{f}_j | \boldsymbol{a}_{\boldsymbol{\psi}_1(j)}\right)} \,\mathrm{d}\boldsymbol{f}\,\mathrm{d}\boldsymbol{a}$$

We recall the definition of $\boldsymbol{\psi}_C(j)$ in Def. 2, where we have $\boldsymbol{\psi}_C(j) = \boldsymbol{\pi}_C(j) \cup \{j, \ldots, C\}$ if $j < C$ and $\boldsymbol{\psi}_C(j) = \boldsymbol{\pi}_C(j) \cup j$ otherwise. Further, we have $\boldsymbol{\pi}_2(j) = \boldsymbol{\pi}_1(j) \cup \phi(j)$ where $\phi(j)$ is the additional predecessor of expert j in the model 2 compared to model 1. Therefore, we have $\boldsymbol{\psi}_2(j) = \boldsymbol{\psi}_1(j) \cup \phi(j)$ for all j.

[Proof: If $j < C$, we have $\boldsymbol{\pi}_1(j) = \boldsymbol{\pi}_2(j)$ since $\phi(j)$ empty. Therefore, we have $\boldsymbol{\psi}_1(j) = \boldsymbol{\pi}_1(j) \cup \{j, \ldots, C\} = \boldsymbol{\pi}_2(j) \cup \{j, \ldots, C\} = \boldsymbol{\psi}_2(j)$ for all $j = 1, \ldots, C-1$. If $j \geq C$, we have $\boldsymbol{\psi}_1(j) = \boldsymbol{\pi}_1(j) \cup j$ and $\boldsymbol{\psi}_2(j) = \boldsymbol{\pi}_2(j) \cup j = \boldsymbol{\pi}_1(j) \cup \phi(j) \cup j = \boldsymbol{\psi}_1(j) \cup \phi(j)$ for all $j = C, \ldots, J$.]

We abbreviate $\boldsymbol{\psi}_1(j) = \boldsymbol{\psi}(j)$ and substitute $\boldsymbol{\psi}_2(j) = \boldsymbol{\psi}(j) \cup \phi(j)$ yielding

$$\begin{aligned}
&\sum_{j=1}^{J} \int p(\boldsymbol{a}, \boldsymbol{f}) \log \frac{p\left(\boldsymbol{f}_j | \boldsymbol{a}_{\boldsymbol{\psi}(j)}, \boldsymbol{a}_{\boldsymbol{\phi}(j)}\right)}{p\left(\boldsymbol{f}_j | \boldsymbol{a}_{\boldsymbol{\psi}(j)}\right)} \, \mathrm{d}\boldsymbol{f} \, \mathrm{d}\boldsymbol{a} \\
=& \sum_{j=1}^{J} \int p(\boldsymbol{a}, \boldsymbol{f}) \log \frac{p\left(\boldsymbol{f}_j | \boldsymbol{a}_{\boldsymbol{\psi}(j)}, \boldsymbol{a}_{\boldsymbol{\phi}(j)}\right) p\left(\boldsymbol{a}_{\boldsymbol{\phi}(j)} | \boldsymbol{a}_{\boldsymbol{\psi}(j)}\right)}{p\left(\boldsymbol{f}_j | \boldsymbol{a}_{\boldsymbol{\psi}(j)}\right) p\left(\boldsymbol{a}_{\boldsymbol{\phi}(j)} | \boldsymbol{a}_{\boldsymbol{\psi}(j)}\right)} \, \mathrm{d}\boldsymbol{a} \\
=& \sum_{j=1}^{J} \int p(\boldsymbol{a}, \boldsymbol{f}) \log \frac{p\left(\boldsymbol{f}_j, \boldsymbol{a}_{\boldsymbol{\phi}(j)} | \boldsymbol{a}_{\boldsymbol{\psi}(j)}\right)}{p\left(\boldsymbol{f}_j | \boldsymbol{a}_{\boldsymbol{\psi}(j)}\right) p\left(\boldsymbol{a}_{\boldsymbol{\phi}(j)} | \boldsymbol{a}_{\boldsymbol{\psi}(j)}\right)} \, \mathrm{d}\boldsymbol{a} \\
=& \sum_{j=1}^{J} \int p(\tilde{\boldsymbol{a}}_j, \boldsymbol{f}_j) \log \frac{p\left(\boldsymbol{f}_j, \boldsymbol{a}_{\boldsymbol{\phi}(j)} | \boldsymbol{a}_{\boldsymbol{\psi}(j)}\right)}{p\left(\boldsymbol{f}_j | \boldsymbol{a}_{\boldsymbol{\psi}(j)}\right) p\left(\boldsymbol{a}_{\boldsymbol{\phi}(j)} | \boldsymbol{a}_{\boldsymbol{\psi}(j)}\right)} \, \mathrm{d}\tilde{\boldsymbol{a}}_j \\
=& \sum_{j=1}^{J} I\left(\boldsymbol{f}_j, \boldsymbol{a}_{\boldsymbol{\phi}(j)} | \boldsymbol{a}_{\boldsymbol{\psi}(j)}\right) \geq 0
\end{aligned}$$

where $\tilde{\boldsymbol{a}}_j = \boldsymbol{a}_{\boldsymbol{\phi}(j)} \cup \boldsymbol{a}_{\boldsymbol{\psi}(j)}$ and $I\left(\boldsymbol{f}_j, \boldsymbol{a}_{\boldsymbol{\phi}(j)} | \boldsymbol{a}_{\boldsymbol{\psi}(j)}\right)$ the conditional mutual information which is always positive [42, p. 30] and therefore concludes the first part of the proof.

Moreover, the difference in the joint prior is

$$\begin{aligned}
\mathbb{D}_{(C,T)}[\boldsymbol{f}, \boldsymbol{a}, \boldsymbol{y}] = \mathbb{D}_{(C,T)}[\boldsymbol{f}, \boldsymbol{a}] = \mathbb{D}_{(C,T)}[\boldsymbol{a}] + \mathbb{D}_{(C,T)}[\boldsymbol{f}|\boldsymbol{a}] \\
= \frac{1}{2} \log \frac{|\bar{\boldsymbol{V}}_C||\boldsymbol{S}_C^{-1}|}{|\bar{\boldsymbol{V}}_{C+T}|\boldsymbol{S}_{C+T}^{-1}||} = \frac{1}{2} \log \frac{|\bar{\boldsymbol{V}}_C||\boldsymbol{Q}_{C+T}|}{|\bar{\boldsymbol{V}}_{C+T}|\boldsymbol{Q}_C||} \geq 0.
\end{aligned}$$

Proof 9 (Subproof III of Proof 6; Prior KL) For the second part, we use (B13) where the difference in KL of 2 Gaussians with zero mean and same base distribution is formulated. In our case we have

$$\frac{1}{2}\left(tr((\boldsymbol{S}_C - \boldsymbol{S}_{C+T})\boldsymbol{K}_{\boldsymbol{AA}}) + \log \frac{|\boldsymbol{S}_{C+T}|}{|\boldsymbol{S}_C|}\right)$$

where the trace is 0 by Prop. 12 and thus $\mathbb{D}_{(C,T)} = \frac{1}{2} \log \frac{|\boldsymbol{S}_{C+T}|}{|\boldsymbol{S}_C|}$. Since $\boldsymbol{S}_C = \boldsymbol{F}^T \boldsymbol{Q}^{-1} \boldsymbol{F}$ and $|\boldsymbol{F}| = 1$, we have $\mathbb{D}_{(C,T)} = \frac{1}{2} \log \frac{|\boldsymbol{Q}_C|}{|\boldsymbol{Q}_{C+T}|}$ which concludes the proof.

Proof 10 (Proof of Prop. 14; Decreasing Prior Entropy) For the third part of the statement, the entropy H of $q_C(\boldsymbol{a})$ is

$$H[q_C(\boldsymbol{a})] = \frac{1}{2}\left(-\log|\boldsymbol{S}_C| + JL(1 + \log 2\pi)\right) = \frac{1}{2}\left(\log|\boldsymbol{Q}_C| + JL(1 + \log 2\pi)\right)$$

where we used Eq. (B10) and $|\boldsymbol{F}| = 1$. The second part follows from Prop. 5. Using Proof 9 which states

$$\mathbb{D}_{(C,T)} = \frac{1}{2} \log \frac{|\boldsymbol{Q}_C|}{|\boldsymbol{Q}_{C+T}|} \geq 0,$$

it follows

$$\log |\boldsymbol{Q}_C| \geq \log |\boldsymbol{Q}_{C+T}|$$

for any $T \in \{1, \ldots, C-1\}$ and therefore

$$H[q_{C+T}(\boldsymbol{a})] \leq H[q_C(\boldsymbol{a})]$$

which concludes the proof.

Proof 11 ((Sub)Proof of Prop. 3; Marginalized Joint Distribution) From the joint distribution in Def. 4 over all variables, the latent function values $\boldsymbol{f}$ can be integrated out resulting in

$$q(\boldsymbol{a}, \boldsymbol{y}) = \int q(\boldsymbol{f}, \boldsymbol{a}, \boldsymbol{y}) \,\mathrm{d}\boldsymbol{f} = \int p(\boldsymbol{y}|\boldsymbol{f})\, q(\boldsymbol{f}|\boldsymbol{a}) \,\mathrm{d}\boldsymbol{f} \; q(\boldsymbol{a}),$$

where the integral can be computed via (B7) yielding

$$q(\boldsymbol{a}, \boldsymbol{y}) = \int p(\boldsymbol{y}|\boldsymbol{f})\, q(\boldsymbol{f}|\boldsymbol{a}) \,\mathrm{d}\boldsymbol{f} = \int \mathcal{N}\left(\boldsymbol{y}|\boldsymbol{f}, \sigma_n^2 \mathbb{I}\right) \mathcal{N}\left(\boldsymbol{f}|\boldsymbol{H}\boldsymbol{f}, \overline{\boldsymbol{V}}\right) \mathrm{d}\boldsymbol{f} = \mathcal{N}(\boldsymbol{y}|\boldsymbol{H}\boldsymbol{a}, \boldsymbol{V})$$

with $\boldsymbol{V} = \overline{\boldsymbol{V}} + \sigma_n^2 \mathbb{I}$ and thus

$$q(\boldsymbol{a}, \boldsymbol{y}) = q(\boldsymbol{y}|\boldsymbol{a})\, q(\boldsymbol{a}) = \mathcal{N}(\boldsymbol{y}|\boldsymbol{H}\boldsymbol{a}, \boldsymbol{V})\, \mathcal{N}\left(\boldsymbol{a}|\boldsymbol{0}, \boldsymbol{S}^{-1}\right)$$

which concludes the proof.

Proof 12 (Proof of Prop.3; Posterior Approximation) The posterior approximation is

$$q(\boldsymbol{a}|\boldsymbol{y}) = \frac{q(\boldsymbol{a}, \boldsymbol{y})}{q(\boldsymbol{y})} \propto q(\boldsymbol{a}, \boldsymbol{y}) = q(\boldsymbol{y}|\boldsymbol{a})\, q_C(\boldsymbol{a})$$

where the first equality comes from the definition of conditional probabilities, the proportionality because the marginal likelihood $q(\boldsymbol{y})$ is independent of $\boldsymbol{a}$ and the last equality exploits Proof 11. Since

$$q(\boldsymbol{y}|\boldsymbol{a})\, q_C(\boldsymbol{a}) = \mathcal{N}(\boldsymbol{y}|\boldsymbol{H}\boldsymbol{a}, \boldsymbol{V})\, \mathcal{N}\left(\boldsymbol{a}|\boldsymbol{0}, \boldsymbol{S}^{-1}\right),$$

the desired posterior distribution can be analytically computed via (B8) yielding

$$q(\boldsymbol{a}|\boldsymbol{y}) = \mathcal{N}(\boldsymbol{a}|\boldsymbol{\mu}, \boldsymbol{\Sigma}),$$

with $\boldsymbol{\Sigma} = \left(\boldsymbol{H}^T \boldsymbol{V}^{-1} \boldsymbol{H} + \boldsymbol{S}\right)^{-1}$, $\boldsymbol{\mu} = \boldsymbol{\Sigma}\boldsymbol{\eta}$ and $\boldsymbol{\eta} = \boldsymbol{H}^T \boldsymbol{V}^{-1} \boldsymbol{y}$.

Proof 13 (Proof of Prop. 9; Marginal Likelihood) The marginal likelihood $q(\boldsymbol{y})$ is obtained by integrating (B7) over the joint distribution $q(\boldsymbol{y}, \boldsymbol{a})$ in Prop. 2 leading to

$$q(\boldsymbol{y}) = \int q(\boldsymbol{y}, \boldsymbol{a}) \,\mathrm{d}\boldsymbol{a} = \int q(\boldsymbol{y}|\boldsymbol{a})\, q(\boldsymbol{a}) \,\mathrm{d}\boldsymbol{a} = \int \mathcal{N}(\boldsymbol{H}\boldsymbol{a}, \boldsymbol{V})\, \mathcal{N}\left(\boldsymbol{0}, \boldsymbol{S}^{-1}\right) \mathrm{d}\boldsymbol{a} = \mathcal{N}(\boldsymbol{0}, \boldsymbol{P})$$

where $\boldsymbol{P} = \boldsymbol{H}\boldsymbol{S}^{-1}\boldsymbol{H}^T + \boldsymbol{V}$.

Proof 14 (Proof of Prop. 12; Exact Diagonal of Prior) Using Prop. 11, the trace can be written as

$$tr\left(\boldsymbol{S}\boldsymbol{K}_{\boldsymbol{A}\boldsymbol{A}}\right) = tr\left(\left(\sum_{j=1}^{J} \overline{\boldsymbol{K}}^{-1}_{\boldsymbol{A}_{\boldsymbol{\pi}^+(j)}\boldsymbol{A}_{\boldsymbol{\pi}^+(j)}} - \overline{\boldsymbol{K}}^{-1}_{\boldsymbol{A}_{\boldsymbol{\pi}(j)}\boldsymbol{A}_{\boldsymbol{\pi}(j)}}\right)\boldsymbol{K}_{\boldsymbol{A}\boldsymbol{A}}\right)$$
$$= \sum_{j=1}^{J} tr\left(\overline{\boldsymbol{K}}^{-1}_{\boldsymbol{A}_{\boldsymbol{\pi}^+(j)}\boldsymbol{A}_{\boldsymbol{\pi}^+(j)}}\boldsymbol{K}_{\boldsymbol{A}\boldsymbol{A}}\right) - tr\left(\overline{\boldsymbol{K}}^{-1}_{\boldsymbol{A}_{\boldsymbol{\pi}(j)}\boldsymbol{A}_{\boldsymbol{\pi}(j)}}\boldsymbol{K}_{\boldsymbol{A}\boldsymbol{A}}\right).$$

By construction of the matrices $\overline{\boldsymbol{K}}^{-1}_{\boldsymbol{A}_\phi,\boldsymbol{A}_\phi}$, they contain the matrix $\boldsymbol{K}^{-1}_{\boldsymbol{A}_\phi,\boldsymbol{A}_\phi}$ at the entries $[\phi,\phi]$. Therefore, the resulting product when multiplying with $\boldsymbol{K}_{\boldsymbol{A}\boldsymbol{A}}$ is a matrix with identity $\mathbb{I}_T$ at the position $[\phi,\phi]$ with $T = |\phi|$ and 0 at the diagonal where not ϕ. The quantity above is then

$$\sum_{j=1}^{J} L|\boldsymbol{\pi}^+(j)| - L|\boldsymbol{\pi}\,(j)\,| = \sum_{j=1}^{J} L\left(min(j,C) - min(j-1,C-1)\right) = JL.$$

Proof 15 (Proof of Prop. 10; Prior Approximation II) The prior approximation is

$$q(\boldsymbol{a}) = \prod_{j=1}^{J} p\left(\boldsymbol{a}_j|\boldsymbol{a}_{\boldsymbol{\pi}(j)}\right) = \prod_{j=1}^{J} \mathcal{N}\left(\boldsymbol{a}_j|\boldsymbol{F}_j\boldsymbol{a}_{\boldsymbol{\pi}(j)}, \boldsymbol{Q}_j\right)$$

for which the quadratic term inside the exponential of the individual Gaussian can be written as

$$-\frac{1}{2}(\boldsymbol{a}_j - \boldsymbol{F}_j\boldsymbol{a}_{\boldsymbol{\pi}(j)})^T\boldsymbol{Q}_j^{-1}(\boldsymbol{a}_j - \boldsymbol{F}_j\boldsymbol{a}_{\boldsymbol{\pi}(j)})$$
$$-\frac{1}{2}\begin{bmatrix}\boldsymbol{a}^T_{\boldsymbol{\pi}(j)} & \boldsymbol{a}^T_j\end{bmatrix}\begin{bmatrix}-\boldsymbol{F}^T_j \\ \mathbb{I}\end{bmatrix}\boldsymbol{Q}_j^{-1}\begin{bmatrix}-\boldsymbol{F}_j & \mathbb{I}\end{bmatrix}\begin{bmatrix}\boldsymbol{a}_{\boldsymbol{\pi}(j)} \\ \boldsymbol{a}_j\end{bmatrix}$$

which correspond to a Gaussian

$$\mathcal{N}\left(\boldsymbol{a}_{\boldsymbol{\pi}^+(j)}|\boldsymbol{0}, \boldsymbol{S}^{-1}_{(j)}\right)$$

with $\boldsymbol{S}_{(j)} = \tilde{\boldsymbol{F}}^T_j\boldsymbol{Q}^{-1}_j\tilde{\boldsymbol{F}}_j \in \mathbb{R}^{LC\times LC}$ and $\tilde{\boldsymbol{F}}_j = \begin{bmatrix}-\boldsymbol{F}_j & \mathbb{I}\end{bmatrix} \in \mathbb{R}^{L\times LC}$ which proves the first part. We can augment this Gaussian for $\boldsymbol{a}_{\boldsymbol{\pi}^+(j)} \in \mathbb{R}^{LC}$ to

$$-\frac{1}{2}\boldsymbol{a}^T\bar{\boldsymbol{S}}^{-1}_{(j)}\boldsymbol{a} \quad \propto \quad \mathcal{N}\left(\boldsymbol{a}|\boldsymbol{0}, \bar{\boldsymbol{S}}^{-1}_{(j)}\right)$$

over $\boldsymbol{a} \in \mathbb{R}^M$ where $\bar{\boldsymbol{S}}_{(j)} \in \mathbb{R}^{M\times M}$ is the augmented matrix consisting of $\boldsymbol{S}_{(j)}$at the entries $[\boldsymbol{\pi}^+(j), \boldsymbol{\pi}^+(j)]$ and 0 otherwise. Using (B9), the original product $q(\boldsymbol{a})$ is then

$$\prod_{j=1}^{J} \mathcal{N}\left(\boldsymbol{a}|\boldsymbol{0}, \bar{\boldsymbol{S}}^{-1}_{(j)}\right) = \mathcal{N}\left(\boldsymbol{a}|\boldsymbol{0}, \left(\sum_{j=1}^{J}\bar{\boldsymbol{S}}_{(j)}\right)^{-1}\right)$$

and thus $\boldsymbol{S} = \sum_{j=1}^{J}\bar{\boldsymbol{S}}_{(j)}$ positive definite which concludes the proof.

Proof 16 (Proof of Prop. 11; Prior Approximation III) The prior $q(\boldsymbol{a})$ can be written as

$$q(\boldsymbol{a}) = \prod_{j=1}^{J} p\left(\boldsymbol{a}_j|\boldsymbol{a}_{\boldsymbol{\pi}(j)}\right) = \prod_{j=1}^{J}\frac{p\left(\boldsymbol{a}_j, \boldsymbol{a}_{\boldsymbol{\pi}(j)}\right)}{p\left(\boldsymbol{a}_{\boldsymbol{\pi}(j)}\right)} = \prod_{j=1}^{J}\frac{p\left(\boldsymbol{a}_{\boldsymbol{\pi}^+(j)}\right)}{p\left(\boldsymbol{a}_{\boldsymbol{\pi}(j)}\right)}$$

$$= \prod_{j=1}^{J} \frac{\mathcal{N}\left(\boldsymbol{a}_{\boldsymbol{\pi}^+(j)} | \mathbf{0}, \boldsymbol{K}_{\boldsymbol{A}_{\boldsymbol{\pi}^+(j)} \boldsymbol{A}_{\boldsymbol{\pi}^+(j)}}\right)}{\mathcal{N}\left(\boldsymbol{a}_{\boldsymbol{\pi}(j)} | \mathbf{0}, \boldsymbol{K}_{\boldsymbol{A}_{\boldsymbol{\pi}(j)} \boldsymbol{A}_{\boldsymbol{\pi}(j)}}\right)}$$

Similarly to the Proof 15, we can augment the CL-dimensional and the $(C-1)L$-dimensional Gaussian in the nominator and denominator, respectively, to M-dimensional Gaussians with covariance $\overline{\boldsymbol{K}}^{-1}_{\boldsymbol{A}_{\boldsymbol{\phi}} \boldsymbol{A}_{\boldsymbol{\phi}}}$ consisting of $\boldsymbol{K}^{-1}_{\boldsymbol{A}_{\boldsymbol{\phi}} \boldsymbol{A}_{\boldsymbol{\phi}}}$ at the entries $[\boldsymbol{\phi}, \boldsymbol{\phi}]$ and 0 otherwise. This gives with (B9)

$$\prod_{j=1}^{J} \frac{\mathcal{N}\left(\boldsymbol{a} | \mathbf{0}, \overline{\boldsymbol{K}}_{\boldsymbol{A}_{\boldsymbol{\pi}^+(j)} \boldsymbol{A}_{\boldsymbol{\pi}^+(j)}}\right)}{\mathcal{N}\left(\boldsymbol{a} | \mathbf{0}, \overline{\boldsymbol{K}}_{\boldsymbol{A}_{\boldsymbol{\pi}(j)} \boldsymbol{A}_{\boldsymbol{\pi}(j)}}\right)}$$

$$= \mathcal{N}\left(\boldsymbol{a} | \mathbf{0}, \left(\sum_{j=1}^{J} \overline{\boldsymbol{K}}^{-1}_{\boldsymbol{A}_{\boldsymbol{\pi}^+(j)} \boldsymbol{A}_{\boldsymbol{\pi}^+(j)}} - \overline{\boldsymbol{K}}^{-1}_{\boldsymbol{A}_{\boldsymbol{\pi}(j)} \boldsymbol{A}_{\boldsymbol{\pi}(j)}}\right)^{-1}\right),$$

which concludes the proof with $\boldsymbol{S} = \sum_{j=1}^{J} \overline{\boldsymbol{K}}^{-1}_{\boldsymbol{A}_{\boldsymbol{\pi}^+(j)} \boldsymbol{A}_{\boldsymbol{\pi}^+(j)}} - \overline{\boldsymbol{K}}^{-1}_{\boldsymbol{A}_{\boldsymbol{\pi}(j)} \boldsymbol{A}_{\boldsymbol{\pi}(j)}}$ which is positive definite.

Proof 17 (Proof of Prop. 4; Prediction Aggregation) The predictive posterior distribution is defined as

$$p\left(f_* | \boldsymbol{y}\right) = \prod_{j=C}^{J} p\left(f_{*j} | \boldsymbol{y}\right)^{\beta_{*j}}.$$

Since the local predictions $p\left(f_{*j} | \boldsymbol{y}\right) = \mathcal{N}\left(m_{*j}, v_{*j}\right)$ are all univariate Gaussians, we obtain via the product rule of Gaussians in (B9) directly

$$m_* = v_{*j} \sum_{j=C}^{J} \beta_{*j} \frac{m_{*j}}{v_{*j}} \quad \text{and} \quad \frac{1}{v_*} = \sum_{j=C}^{J} \frac{\beta_{*j}}{v_{*j}}.$$

Using the usual likelihood $p\left(y_* | f_*\right) = \mathcal{N}\left(f_*, \sigma_n^2\right)$ yields with (B7) the final noisy prediction $p\left(y_* | \boldsymbol{y}\right) = \int p\left(y_* | f_*\right) p\left(f_* | \boldsymbol{y}\right) \mathrm{d} f_* = \mathcal{N}\left(m_*, v_* + \sigma_n^2\right)$. Note that, in Definition 5 of the weights, we introduced a scaling factor Z. This Z in the exponent of the normalization of the weights has a sharpening effect, so that the informative experts have even more weight compared to the non-informative experts for more data N and more correlations C. This is a heuristic but showed quite robust performance in experiments. Moreover, the consistency properties are more relevant than the particular weights.

Proof 18 (Proof of Prop. 8; Local Predictions) The predictive conditional $p\left(f_{*j} | \boldsymbol{a}_{\boldsymbol{\psi}(j)}\right)$ can be again derived via (B6) from the assumed joint

$$p\left(f_{*j}, \boldsymbol{a}_{\boldsymbol{\psi}(j)}\right) = \mathcal{N}\left(\mathbf{0}, \boldsymbol{K}_{[\boldsymbol{x}_*, \boldsymbol{A}_{\boldsymbol{\psi}(j)}][\boldsymbol{x}_*, \boldsymbol{A}_{\boldsymbol{\psi}(j)}]}\right)$$

leading to $\mathcal{N}\left(\boldsymbol{h}_* \boldsymbol{a}_{\boldsymbol{\psi}(j)}, v_*\right)$ with

$$\boldsymbol{h}_* = \boldsymbol{K}_{\boldsymbol{x}_* \boldsymbol{A}_{\boldsymbol{\psi}(j)}} \boldsymbol{K}^{-1}_{\boldsymbol{A}_{\boldsymbol{\psi}(j)} \boldsymbol{A}_{\boldsymbol{\psi}(j)}}$$

and

$$v_* = \boldsymbol{K}_{\boldsymbol{x}_*\boldsymbol{x}_*} - \boldsymbol{K}_{\boldsymbol{x}_*\boldsymbol{A}_{\psi(j)}} \boldsymbol{K}^{-1}_{\boldsymbol{A}_{\psi(j)}\boldsymbol{A}_{\psi(j)}} \boldsymbol{K}_{\boldsymbol{A}_{\psi(j)}\boldsymbol{x}_*}.$$

Moreover, the local posteriors $q\left(\boldsymbol{a}_{\boldsymbol{\psi}(j)}|\boldsymbol{y}\right) = \mathcal{N}\left(\boldsymbol{\mu}_{\boldsymbol{\psi}(j)}, \boldsymbol{\Sigma}_{\boldsymbol{\psi}(j)}\right)$ are obtained from the corresponding entries $\boldsymbol{\psi}(j)$ of the mean $\boldsymbol{\mu}$ and covariance $\boldsymbol{\Sigma}$ (via partial inversion A.2) in Prop, 3. Finally, the local predictions $p\left(f_{*j}|\boldsymbol{y}\right)$ in Prop. (8) can then be computed with Gaussian integration (B7) yielding

$$q\left(f_{*j}|\boldsymbol{y}\right) = \int p\left(f_{*j}|\boldsymbol{a}_{\boldsymbol{\psi}(j)}\right) p\left(\boldsymbol{a}_{\boldsymbol{\psi}(j)}|\boldsymbol{y}\right) \mathrm{d}\boldsymbol{a}_{\boldsymbol{\psi}(j)}$$

which correspond to the desired quantities

$$\mathcal{N}\left(m_{*j}, v_{*j}\right) = \mathcal{N}\left(\boldsymbol{h}_*\boldsymbol{\mu}_{\boldsymbol{\psi}(j)}, \boldsymbol{h}_*^T\boldsymbol{\Sigma}_{\boldsymbol{\psi}(j)}\boldsymbol{h}_* + v_*\right).$$

Proof 19 (Proof for Figure C7; Joint Prior Covariance) For the joint prior

$$q_C(\boldsymbol{a}, \boldsymbol{f}, \boldsymbol{y}) = \mathcal{N}\left([\boldsymbol{a};\ \boldsymbol{f};\ \boldsymbol{y}] \mid \boldsymbol{0}, \boldsymbol{W}_\gamma\right)$$

with covariance

$$\boldsymbol{W}_\gamma = \begin{bmatrix} \boldsymbol{\Sigma}_{\boldsymbol{aa}} & \boldsymbol{\Sigma}_{\boldsymbol{af}} & \boldsymbol{\Sigma}_{\boldsymbol{ay}} \\ \boldsymbol{\Sigma}_{\boldsymbol{fa}} & \boldsymbol{\Sigma}_{\boldsymbol{ff}} & \boldsymbol{\Sigma}_{\boldsymbol{fy}} \\ \boldsymbol{\Sigma}_{\boldsymbol{ya}} & \boldsymbol{\Sigma}_{\boldsymbol{yf}} & \boldsymbol{\Sigma}_{\boldsymbol{yy}} \end{bmatrix}$$

corresponding to Fig. C7, we show that we recover the marginal and conditional distributions $q_C(\boldsymbol{a})$, $q_C(\boldsymbol{f}|\boldsymbol{a})$ and $p(\boldsymbol{y}|\boldsymbol{f})$. For $q_C(\boldsymbol{a})$, the marginalization correspond to selecting the corresponding mean and covariance, i.e. $\mathcal{N}\left(\boldsymbol{a}|\boldsymbol{0}, \boldsymbol{\Sigma}_{\boldsymbol{aa}}\right) = \mathcal{N}\left(\boldsymbol{a}|\boldsymbol{0}, \boldsymbol{S}^{-1}\right)$. For $q_C(\boldsymbol{f}|\boldsymbol{a})$, we use Eq. (B6) yielding

$$\begin{aligned} &\mathcal{N}\left(\boldsymbol{f}|\boldsymbol{\Sigma}_{\boldsymbol{fa}}\boldsymbol{\Sigma}_{\boldsymbol{aa}}^{-1}\boldsymbol{a}, \boldsymbol{\Sigma}_{\boldsymbol{ff}} - \boldsymbol{\Sigma}_{\boldsymbol{fa}}\boldsymbol{\Sigma}_{\boldsymbol{aa}}^{-1}\boldsymbol{\Sigma}_{\boldsymbol{af}}\right) \\ &= \mathcal{N}\left(\boldsymbol{f}|\boldsymbol{H}\boldsymbol{a}, (\boldsymbol{H}\boldsymbol{S}^{-1}\boldsymbol{H}^T + \overline{\boldsymbol{V}}) - \boldsymbol{H}(\boldsymbol{S}^{-1}\boldsymbol{H}^T\right) \\ &= \mathcal{N}\left(\boldsymbol{f}|\boldsymbol{H}\boldsymbol{a}, \overline{\boldsymbol{V}}\right) \end{aligned}$$

since $\boldsymbol{\Sigma}_{\boldsymbol{fa}}\boldsymbol{\Sigma}_{\boldsymbol{aa}}^{-1} = (\boldsymbol{H}\boldsymbol{S}^{-}1)\boldsymbol{S} = \boldsymbol{H}$. Similarly for $p(\boldsymbol{y}|\boldsymbol{f})$, with Eq. (B6) we get

$$\begin{aligned} &\mathcal{N}\left(\boldsymbol{y}|\boldsymbol{\Sigma}_{\boldsymbol{yf}}\boldsymbol{\Sigma}_{\boldsymbol{ff}}^{-1}\boldsymbol{f}, \boldsymbol{\Sigma}_{\boldsymbol{yy}} - \boldsymbol{\Sigma}_{\boldsymbol{yf}}\boldsymbol{\Sigma}_{\boldsymbol{ff}}^{-1}\boldsymbol{\Sigma}_{\boldsymbol{fy}}\right) \\ &= \mathcal{N}\left(\boldsymbol{y}|\mathbb{I}\boldsymbol{f}, (\boldsymbol{H}\boldsymbol{S}^{-1}\boldsymbol{H}^T + \boldsymbol{V}) - \mathbb{I}(\boldsymbol{H}\boldsymbol{S}^{-1}\boldsymbol{H}^T + \overline{\boldsymbol{V}})\right) \\ &= \mathcal{N}\left(\boldsymbol{y}|\boldsymbol{f}, \sigma_n^2\mathbb{I}\right) \end{aligned}$$

since $\boldsymbol{\Sigma}_{\boldsymbol{yf}}\boldsymbol{\Sigma}_{\boldsymbol{ff}}^{-1} = \mathbb{I}$.

C.3 Derivative of LML

The log marginal likelihood in Section 3.6.2 in Eq. (A1) is proportional to

$$-\frac{1}{2}\boldsymbol{y}^T\boldsymbol{V}^{-1}\boldsymbol{y} + \frac{1}{2}\boldsymbol{\mu}^T\boldsymbol{\Sigma}^{-1}\boldsymbol{\mu} - \frac{1}{2}\log|\boldsymbol{\Sigma}^{-1}| - \frac{1}{2}\log|\boldsymbol{V}| - \frac{1}{2}\log|\boldsymbol{Q}|.$$

In the following, we provide the partial derivative with respect to θ for each additive term.

$$\frac{\partial}{\partial\theta}\left[-\frac{1}{2}\boldsymbol{y}^T\boldsymbol{V}^{-1}\boldsymbol{y}\right]=\frac{1}{2}\boldsymbol{y}^T\boldsymbol{V}^{-1}\frac{\partial\boldsymbol{V}}{\partial\theta}\boldsymbol{V}^{-1}\boldsymbol{y}$$

$$\frac{\partial}{\partial\theta}\left[\frac{1}{2}\boldsymbol{\mu}^T\boldsymbol{\Sigma}^{-1}\boldsymbol{\mu}\right]=\frac{\partial\boldsymbol{\eta}^T}{\partial\theta}\boldsymbol{\mu}-\frac{1}{2}\boldsymbol{\mu}^T\frac{\partial\boldsymbol{\Sigma}^{-1}}{\partial\theta}\boldsymbol{\mu}$$

$$\frac{\partial}{\partial\theta}\left[-\frac{1}{2}\log|\boldsymbol{\Sigma}^{-1}|\right]=-\frac{1}{2}tr\left\{\boldsymbol{\Sigma}\frac{\partial\boldsymbol{\Sigma}^{-1}}{\partial\theta}\right\}$$

In the last expression the whole posterior covariance is needed, however, it turns out that only the entries which are non-zero in the precision are needed. The right term in the last expression equals $sum\left\{\boldsymbol{\Sigma}\odot\frac{\partial\boldsymbol{\Sigma}^{-1}}{\partial\theta}\right\}$, where $\odot$ denotes the pointwise multiplication. Therefore it is enough to only compute $sum\left\{\overline{\boldsymbol{\Sigma}}\odot\frac{\partial\boldsymbol{\Sigma}^{-1}}{\partial\theta}\right\}$, where $\overline{\boldsymbol{\Sigma}}$ is the partial inversion (for more derails A.2) which is sparse as well and already computed for the local predictions in Prop. 8.

$$\frac{\partial}{\partial\theta}\left[-\frac{1}{2}\log|\boldsymbol{V}|\right]=-\frac{1}{2}sum\left\{\boldsymbol{V}^{-1}\odot\frac{\partial\boldsymbol{V}}{\partial\theta}\right\}$$

$$\frac{\partial}{\partial\theta}\left[-\frac{1}{2}\log|\boldsymbol{Q}|\right]=-\frac{1}{2}sum\left\{\boldsymbol{Q}^{-1}\odot\frac{\partial\boldsymbol{Q}}{\partial\theta}\right\}$$

The derivatives $\frac{\partial\boldsymbol{\Sigma}^{-1}}{\partial\theta}$, $\frac{\partial\boldsymbol{V}}{\partial\theta}$ and $\frac{\partial\boldsymbol{Q}}{\partial\theta}$ can be computed via chain rule of derivatives.

Appendix D Sequential Algorithm

The probabilistic equations in Section 3 can be equivalently formulated as

$$\begin{aligned}\boldsymbol{a}_j&=\boldsymbol{F}_j\boldsymbol{a}_{\boldsymbol{\pi}(j)}+\boldsymbol{\gamma}_j;\\ \boldsymbol{f}_j&=\boldsymbol{H}_j\boldsymbol{a}_{\boldsymbol{\psi}(j)}+\boldsymbol{\nu}_j;\\ \boldsymbol{y}_j&=\boldsymbol{f}_j+\boldsymbol{\varepsilon}_j,\end{aligned}$$

with $\boldsymbol{\gamma}_j\sim\mathcal{N}\left(0,\boldsymbol{Q}_j\right)$, $\boldsymbol{\nu}_j\sim\mathcal{N}\left(0,\overline{\boldsymbol{V}}_j\right)$ and $\boldsymbol{\varepsilon}_j\sim\mathcal{N}\left(0,\sigma_n^2\mathbb{I}\right)$. Instead to the inference procedure described in Prop. 3, the posterior could be alternatively computed with sequential algorithms. Assuming $C=2$ and $\boldsymbol{\pi}(j)=\{j-1\}$, the Kalman Filter and Smoother (e.g. [43]) provide an equivalent solution to

the posterior distribution in Prop. 3. For $C > 2$ and general neighbourhood set, the Gaussian (loopy) belief propagation algorithm or Gaussian expectation propagation (e.g. [43]) might constitute an interesting approach for sequential/online and distributed learning procedures. Together with the competitive results in the application to time series with covariates makes this idea very promising for future work.

Appendix E More Details about GPR

In this section we provide more details for Section 2.

Suppose we are given a training set $\mathcal{D} = \{y_i, \boldsymbol{x}_i\}_{i=1}^N$ of N pairs of inputs $\boldsymbol{x}_i \in \mathbb{R}^D$ and noisy scalar outputs y_i generated by adding independent Gaussian noise to a latent function $f(\boldsymbol{x})$, that is $y_i = f(\boldsymbol{x}_i) + \varepsilon_i$, where $\varepsilon_i \sim \mathcal{N}\left(0, \sigma_n^2\right)$. We denote $\boldsymbol{y} = [y_1, \ldots, y_N]^T$ the vector of observations and with $\boldsymbol{X} = [\boldsymbol{x}_1^T, \ldots, \boldsymbol{x}_N^T]^T \in \mathbb{R}^{N \times D}$.
We can model f with a *Gaussian Process* (GP), which defines a prior over functions and can be converted into a posterior over functions once we have observed some data (consider e.g. [1]). To describe a GP, we only need to specify a mean $m(\boldsymbol{x})$ and a covariance function $k_{\boldsymbol{\theta}}(\boldsymbol{x}, \boldsymbol{x}')$ where $\boldsymbol{\theta}$ is a set of a few hyperparemeters. Thereby, $k_{\boldsymbol{\theta}}$ is a positive definite kernel function (see [1]), for instance the *squared exponential* (SE) kernel with individual lengthscales for each dimension, that is $k_{\boldsymbol{\theta}}(\boldsymbol{x}, \boldsymbol{x}') = \sigma_0^2 \exp\left(-\frac{1}{2}\left(\boldsymbol{x} - \boldsymbol{x}'\right)^T \boldsymbol{L}^{-1}\left(\boldsymbol{x} - \boldsymbol{x}'\right)\right)$ with $L = \mathrm{Diag}\left[l_1^2, \ldots, l_D^2\right]$ and $\{\sigma_0, l_1, \ldots, l_D\} \in \boldsymbol{\theta}$. For the sake of simplicity, we assume $m(x) \equiv 0$, however it could be any function. Given the training values $\boldsymbol{f} = f\left(\boldsymbol{X}\right) = \left[f(\boldsymbol{x}_1), \ldots, f(\boldsymbol{x}_N)\right]^T$ and a test latent function value $f_* = f(\boldsymbol{x}_*)$ at a test point $\boldsymbol{x}_* \in \mathbb{R}^D$, then the joint distribution $p(\boldsymbol{f}, f_*)$ is Gaussian $\mathcal{N}\left(\boldsymbol{0}, \boldsymbol{K}_{[\boldsymbol{X};\, \boldsymbol{x}_*][\boldsymbol{X};\, \boldsymbol{x}_*]}\right)$. Thereby, we use the notation $[\boldsymbol{A}_1;\ \boldsymbol{A}_2]$ for the resulting matrix after stacking $\boldsymbol{A}_1 \in \mathbb{R}^{N_1 \times D}$ and $\boldsymbol{A}_2 \in \mathbb{R}^{N_1 \times D}$ above each other and $\boldsymbol{K} \in \mathbb{R}^{M_1 \times M_2}$ denotes the kernel covariance matrix with entries $[\boldsymbol{K}_{\boldsymbol{AB}}]_{ij}$ corresponding to the kernel evaluation $k_{\boldsymbol{\theta}}(\boldsymbol{a}_i, \boldsymbol{b}_j)$ with the corresponding rows $\boldsymbol{a}_i, \boldsymbol{b}_j$ for any $\boldsymbol{A} \in \mathbb{R}^{M_1 \times D}$ and $\boldsymbol{B} \in \mathbb{R}^{M_2 \times D}$.
Typically, in GP regression, the likelihood is Gaussian, that is, $p\left(\boldsymbol{y}|\boldsymbol{f}\right) = \mathcal{N}\left(\boldsymbol{y}|\boldsymbol{f}, \sigma_n^2\mathbb{I}\right)$, and with Bayes theorem (B8) we obtain analytically the predictive posterior distribution $p\left(f_*|\boldsymbol{y}\right) = \mathcal{N}\left(f_*|\boldsymbol{\mu}_*, \boldsymbol{\Sigma}_*\right)$ with $\boldsymbol{\mu}_* = \boldsymbol{K}_{\boldsymbol{x}_*\boldsymbol{X}}\left(\boldsymbol{K}_{\boldsymbol{X}\boldsymbol{X}} + \sigma_n^2\mathbb{I}\right)^{-1}\boldsymbol{y}$ and $\boldsymbol{\Sigma}_* = \boldsymbol{K}_{\boldsymbol{x}_*\boldsymbol{x}_*} - \boldsymbol{K}_{\boldsymbol{x}_*\boldsymbol{X}}\left(\boldsymbol{K}_{\boldsymbol{X}\boldsymbol{X}} + \sigma_n^2\mathbb{I}\right)^{-1}\boldsymbol{K}_{\boldsymbol{X}\boldsymbol{x}_*}$.
Alternatively to the standard derivation shown above, the posterior distribution over the latent variables $\boldsymbol{f}$ given the data $\boldsymbol{y}$ can be explicitly formulated as

$$p\left(\boldsymbol{f}|\boldsymbol{y}\right) \propto p\left(\boldsymbol{f}, \boldsymbol{y}\right) = p\left(\boldsymbol{y}|\boldsymbol{f}\right)p\left(\boldsymbol{f}\right) = \prod_{j=1}^{J} p\left(\boldsymbol{y}_j|\boldsymbol{f}_j\right)p\left(\boldsymbol{f}_j|\boldsymbol{f}_{1:j-1}\right), \tag{E16}$$

where the data is split into J mini-batches of size B, i.e. $\mathcal{D} = \{\boldsymbol{y}_j, \boldsymbol{X}_j\}_{j=1}^J$ with inputs $\boldsymbol{X}_j \in \mathbb{R}^{B\times D}$, outputs $\boldsymbol{y}_j \in \mathbb{R}^B$ and the corresponding latent function values $\boldsymbol{f}_j = f(\boldsymbol{X}_j) \in \mathbb{R}^B$. In (1) we used the notation $\boldsymbol{f}_{k:j}$ indicating $[\boldsymbol{f}_k, \ldots, \boldsymbol{f}_j]$ and the conditionals $p\left(\boldsymbol{f}_j|\boldsymbol{f}_{1:j-1}\right)$ can be derived from the joint Gaussian $p\left(\boldsymbol{f}_j, \boldsymbol{f}_{1:j-1}\right) = \mathcal{N}\left(\mathbf{0}, \boldsymbol{K}_{[\boldsymbol{X}_j;\, \boldsymbol{X}_{1:j-1}][\boldsymbol{X}_j;\, \boldsymbol{X}_{1:j-1}]}\right)$ via Gaussian conditioning (B6). The corresponding graphical model of (1) is depicted in Figure 1(a)i). Given the posterior over $\boldsymbol{f}|\boldsymbol{y}$, the predictive posterior distribution from above is equivalently obtained as $p\left(f_*|\boldsymbol{y}\right) = \int p\left(f_*|\boldsymbol{f}\right) p\left(\boldsymbol{f}|\boldsymbol{y}\right) \mathrm{d}\boldsymbol{f}$ via Gaussian integration (B7) where $p\left(f_*|\boldsymbol{f}\right)$ is derivable from the joint via (B6). The graphical model of the prediction procedure is depicted in Figure 1(b)i). We present this alternative two stage procedure to highlight later connections to our model with full GP.

E.1 Global Sparse GPs

Sparse GP regression approximations based on *global inducing points* reduce the computational complexity by introducing $M \ll N$ inducing points $\boldsymbol{a} \in \mathbb{R}^M$ that optimally summarize the dependency of the whole training data globally, compare the graphical model in Figure 1b). Thereby the inducing *inputs* $\boldsymbol{A} \in \mathbb{R}^{M\times D}$ are in the D-dimensional input data space and the inducing *outputs* $\boldsymbol{a} = f(\boldsymbol{A}) \in \mathbb{R}^M$ are the corresponding GP-function values. In the following, this model is denoted by SGP(M). Similarly to full GP in Eq. (1), the posterior over the inducing points $p(\boldsymbol{a}|\boldsymbol{y}) \propto \int p\left(\boldsymbol{a}, \boldsymbol{f}, \boldsymbol{y}\right) \mathrm{d}\boldsymbol{f}$ can be derived from the joint distribution

$$p\left(\boldsymbol{a}, \boldsymbol{f}, \boldsymbol{y}\right) = p\left(\boldsymbol{y}|\boldsymbol{f}\right) p\left(\boldsymbol{f}|\boldsymbol{a}\right) p(\boldsymbol{a}) = \prod_{j=1}^{J} p\left(\boldsymbol{y}_j|\boldsymbol{f}_j\right) p\left(\boldsymbol{f}_j|\boldsymbol{a}\right) p(\boldsymbol{a}_j|\boldsymbol{a}_{1:j-1}), \quad \text{(E17)}$$

where the usual Gaussian likelihood $p\left(\boldsymbol{y}_j|\boldsymbol{f}_j\right) = \mathcal{N}\left(\boldsymbol{f}_j, \sigma_n^2\mathbb{I}\right)$ is used and $p\left(\boldsymbol{f}_j|\boldsymbol{a}\right)$ can be derived from the joint Gaussian $p\left(\boldsymbol{f}_j, \boldsymbol{a}\right) = \mathcal{N}\left(\mathbf{0}, \boldsymbol{K}_{[\boldsymbol{X}_j;\, \boldsymbol{A}][\boldsymbol{X}_j;\, \boldsymbol{A}]}\right)$ with (B6). Using the posterior computed via (2) together with the predictive conditional $p\left(f_*|\boldsymbol{a}\right)$ derived by (B6) from the assumed joint $p\left(f_*, \boldsymbol{a}\right) = \mathcal{N}\left(\mathbf{0}, \boldsymbol{K}_{[\boldsymbol{x}_*, \boldsymbol{A}][\boldsymbol{x}_*, \boldsymbol{A}]}\right)$ and integrating $\int p\left(f_*|\boldsymbol{a}\right) p\left(\boldsymbol{f}_j, \boldsymbol{a}\right) \mathrm{d}\boldsymbol{a}$ via (B7) provides an approximation to the predictive posterior of full GP. Batch inference in these sparse global models can be done in $\mathcal{O}(M^2N)$ time and $\mathcal{O}(MN)$ space (e.g. [3]).
In order to find optimal inducing inputs $\boldsymbol{A}$ and hyperparameters $\boldsymbol{\theta}$, a sparse variation of the log marginal likelihood similar can be used e.g. [5–7]. In particular, the authors in [5] proposed to maximize a variational lower bound to the true GP marginal likelihood which has the effect that the sparse GP predictive distribution converges to the full GP predictive distribution as the number of inducing points increases. For larger datasets, stochastic optimization has been applied e.g. [8–11] to obtain faster and more data efficient optimization procedures. For recent reviews on the subject consider e.g. [1, 3, 19].

E.2 Local Independent GPs

An alternative to the global sparse inducing point methods constitute local approaches, which exploit multiple local GPs combined with averaging techniques to boost predictions. Beside other averaging techniques (e.g. mixture of experts [28, 28, 29]), the *Product of Expert (PoE)* scheme was proposed by [14], where individual predictions $p\left(f_{*j}|\boldsymbol{y}_j\right)$ from J experts based on the local data $\boldsymbol{y}_j$ are aggregated to the final predictive distribution

$$p\left(f_*|\boldsymbol{y}\right) = \prod_{j=1}^{J} g_j\left(p\left(f_{*j}|\boldsymbol{y}_j\right)\right), \tag{E18}$$

where g_j is a function depending on the particular PoE method discussed below and is in the original work of [14] just the identity. Note that we present here the version of PoEs where the noiseless predictions f_{*j} are aggregated instead of noisy aggregation with y_{*j} as described in some work of PoEs. The individual predictions $p\left(f_{*j}|\boldsymbol{y}_j\right)$ are local GP fits $\int p\left(f_{*j}|\boldsymbol{f}_j\right)p\left(\boldsymbol{f}_j|\boldsymbol{y}_j\right)\mathrm{d}\boldsymbol{f}_j$ involving the predictive conditionals $p\left(f_{*j}|\boldsymbol{f}_j\right)$ derived by (B6) from the assumed joint $p\left(f_{*j},\boldsymbol{f}_j\right) = \mathcal{N}\left(\boldsymbol{0},\boldsymbol{K}_{[\boldsymbol{x}_*,\boldsymbol{X}_j][\boldsymbol{x}_*,\boldsymbol{X}_j]}\right)$ and the local posteriors $p\left(\boldsymbol{f}_j|\boldsymbol{y}_j\right) \propto p\left(\boldsymbol{y}_j|\boldsymbol{f}_j\right)p\left(\boldsymbol{f}_j\right)$, where the individual prior $p\left(\boldsymbol{f}_j\right) = \mathcal{N}\left(\boldsymbol{0},\boldsymbol{K}_{\boldsymbol{X}_j\boldsymbol{X}_j}\right)$. Together with the usual Gaussian likelihood $p\left(\boldsymbol{y}_j|\boldsymbol{f}_j\right) = \mathcal{N}\left(\boldsymbol{f}_j,\sigma_n^2\mathbb{I}\right)$, the final noisy predictive distribution $p\left(y_*|\boldsymbol{y}\right)$ can be obtained via $\int p\left(y_*|f_*\right)p\left(f_*|\boldsymbol{y}\right)\mathrm{d}f_*$. Similarly to Eqs. (1) and (2), the implicit posterior in all PoE method is

$$p\left(\boldsymbol{f}|\boldsymbol{y}\right) \propto p\left(\boldsymbol{f},\boldsymbol{y}\right) = p\left(\boldsymbol{y}|\boldsymbol{f}\right)\prod_{j=1}^{J} p\left(\boldsymbol{f}_j\right) = \prod_{j=1}^{J} p\left(\boldsymbol{y}_j|\boldsymbol{f}_j\right)p\left(\boldsymbol{f}_j\right), \tag{E19}$$

where the corresponding graphical model is depicted in Figure 1iii).
The function g_j in (E18) takes as argument the predictive distribution $p_{*j} := p\left(f_{*j}|\boldsymbol{y}_j\right)$ which depends implicitly also on $\boldsymbol{x}_*$. In the original work [14] the authors used the identity $g_j(p_{*j}) = p_{*j}$ which produce underconfident prediction variances [19]. In order to mitigate this issue, the aggregation weights $g_j(p_{*j}) = p_{*j}^{1/J}$ were proposed [12] but still resulting in too large predictive uncertainty estimates [19]. The reason is that the experts are all equally weighted, however, the predictions at a particular point $\boldsymbol{x}_*$ are not equally reliable, therefore in the generalized PoE (GPoE) [12] some varying weights $\beta_j(x_*)$ were introduced to quantify the contribution of the expert j at $\boldsymbol{x}_*$. Thus, $g_j(p_{*j}) = p_{*j}^{\beta_j(x_*)}$ with weights set to the difference in entropy between the expert's prior and posterior, that is, $\bar{\beta}_{*j} = \frac{1}{2}\log\left(\frac{v_{*0}}{v_{*j}}\right)$. This has the effect of increase or decreasing the importance of the experts based on the corresponding prediction uncertainty v_{*0} and v_{*j}. However, these general weights can produce overconfident uncertainty estimates, therefore the authors in [12] proposed also an version with normalized weights such that $\sum_j^J \bar{\beta}_j(x_*) = 1$.

In the following, PoE and GPoE refer to the version with normalized weights. Other important contributions in this field are BCM [16] and its robustified version RBCM [12], GRBCM [17], distributed local GPs [13], hierarchical PoEs [26], and local experts with consistent aggregations [15, 27]. We refer to [19] for a recent overview.

Simple baseline methods are the *minimal variance (minVar)* and the *nearest expert (NE)* aggregation, where only the prediction from the expert with minimal variance or nearest expert is used, respectively. Although both these method show often surprisingly good performance, they suffer from an huge disadvantage, namely that there are serious discontinuities at the boundaries between the experts (see for instance Fig. 2) and thus often not useful in practice. This is also the main limitation of all local methods based only on the prediction of one expert (e.g. [23–25, 44]) and it was one of the reason for introducing smooth PoEs with combined experts. Since in basically all cases minVar is better than NE (which is also consistent with the findings in [15]), we only compare our method to minVar and not NE for the sake of simplicity.

Appendix F Tables

Here we provide more results for the experiments in Section 4 and the datasets in Table 3a. In the following, we report different average quantities for several test points $\boldsymbol{x}_*, y_*$ corresponding to the predictive distributions $p(y_*|\boldsymbol{y}) = \mathcal{N}(m_*, v_*)$. The considered quantities are *Kullback-Leibler-(KL)-divergence (KL)* to full GP, *Continuous Ranked Probability Score (CRPS)* and *95%-coverage (COV)*, *root mean squared error (RMSE)*, *absolut error (ABSE)*, *negative log probability (NLP)* , root mean squared error to full GP (ERR) and *log marginal likelihood (LML)*.

We use the KL to compare the closeness of predictive distributions of different GP approximation models to the one of full GP $\mathcal{N}(m, v)$. Since both are univariate Gaussians, the $KL(\mathcal{N}(m, v) \parallel \mathcal{N}(m_*, v_*))$ can be computed as $\frac{1}{2}\left(\log\frac{v_*}{v} + \frac{v}{v_*} + \frac{(m-m_*)^2}{v_*} - 1\right)$.

The CRPS can be used to assess the respective accuracy of two probabilistic forecasting models. In particular, it is a measure between the forecast CDF F_* of $\mathcal{N}(m_*, v_*)$ and the empirical CDF of the observation $\boldsymbol{y}_*$ and is defined as $CRPS(F_*, y_*) \int (F(z) - 1_{z \geq y_*})^2 \, \mathrm{d}z$.

The *95%-confidence interval* can be computed as $c_{1,2} = m_* \pm 1.96\sqrt{v_*}$. The 95%-coverage is then defined as $COV = 1_{c_1 \leq y_* \leq c_2}$.

The negative log probability is $-p(y_*|\boldsymbol{y}) = \frac{1}{2}\log(2\pi v_*) + \frac{(y_*-m_*)^2}{2v_*}$.

For all quantities except LML (large values are better) and COV (should be close to 0.95), small values mean better predictions.

	time	LML	KL	ERR	CRPS	RMSE	ABSE	NLP	COV
fullGP	7.3 ± 0.6	-314.2 ± 5.1	0.0 ± 0.0	0.0 ± 0.0	0.162 ± 0.004	0.311 ± 0.011	0.218 ± 0.005	0.47 ± 0.12	0.92 ± 0.01
SGP(25)	6.4 ± 0.6	-595.4 ± 10.7	440.3 ± 19.6	0.314 ± 0.008	0.234 ± 0.005	0.422 ± 0.01	0.324 ± 0.005	1.11 ± 0.04	0.96 ± 0.01
SGP(50)	14.5 ± 2.6	-539.6 ± 10.2	405.0 ± 31.3	0.291 ± 0.012	0.222 ± 0.004	0.402 ± 0.008	0.308 ± 0.005	1.01 ± 0.03	0.95 ± 0.01
SGP(100)	36.4 ± 2.9	-494.6 ± 7.8	352.9 ± 29.5	0.264 ± 0.011	0.211 ± 0.004	0.384 ± 0.007	0.292 ± 0.006	0.92 ± 0.03	0.95 ± 0.01
minVar	1.5 ± 0.1	-389.8 ± 2.9	122.2 ± 13.1	0.156 ± 0.012	0.175 ± 0.004	0.335 ± 0.011	0.236 ± 0.005	0.61 ± 0.09	0.92 ± 0.01
GPoE	1.4 ± 0.1	-389.8 ± 2.9	174.4 ± 9.4	0.166 ± 0.01	0.186 ± 0.004	0.342 ± 0.01	0.255 ± 0.007	0.68 ± 0.05	0.96 ± 0.01
BCM	1.4 ± 0.1	-389.8 ± 2.9	338.1 ± 32.7	0.185 ± 0.012	0.195 ± 0.005	0.354 ± 0.01	0.265 ± 0.007	1.16 ± 0.12	0.82 ± 0.01
RBCM	1.4 ± 0.1	-389.8 ± 2.9	427.9 ± 35.0	0.166 ± 0.013	0.187 ± 0.005	0.342 ± 0.011	0.249 ± 0.006	1.43 ± 0.21	0.79 ± 0.01
GRBCM	1.7 ± 0.1	-465.0 ± 3.1	224.6 ± 30.3	0.202 ± 0.011	0.19 ± 0.004	0.352 ± 0.01	0.262 ± 0.006	0.71 ± 0.05	0.92 ± 0.01
**CPoE(1)**	1.5 ± 0.0	-397.0 ± 2.8	111.1 ± 12.5	0.146 ± 0.011	0.175 ± 0.004	0.333 ± 0.011	0.237 ± 0.006	**0.59 ± 0.09**	0.93 ± 0.01
**CPoE(2)**	2.1 ± 0.1	-345.1 ± 5.6	89.6 ± 14.3	0.124 ± 0.013	0.172 ± 0.004	0.326 ± 0.011	0.232 ± 0.006	0.6 ± 0.1	0.91 ± 0.01
**CPoE(3)**	2.5 ± 0.1	-337.0 ± 5.5	82.2 ± 14.3	0.116 ± 0.013	**0.17 ± 0.004**	**0.323 ± 0.01**	0.231 ± 0.005	**0.59 ± 0.1**	0.91 ± 0.01
**CPoE(4)**	2.8 ± 0.1	-339.4 ± 5.0	**79.5 ± 13.9**	**0.111 ± 0.012**	0.171 ± 0.004	0.324 ± 0.011	0.232 ± 0.005	0.6 ± 0.1	0.91 ± 0.01

Table F5: Results for dataset *concrete*.

	time	LML	KL	ERR	CRPS	RMSE	ABSE	NLP	COV
fullGP	25.5 ± 1.1	-994.2 ± 1.1	0.0 ± 0.0	0.0 ± 0.0	0.283 ± 0.002	0.511 ± 0.004	0.39 ± 0.005	1.49 ± 0.02	0.94 ± 0.0
SGP(25)	7.5 ± 0.7	-1082.8 ± 0.9	93.49 ± 3.86	0.232 ± 0.005	0.316 ± 0.003	0.561 ± 0.004	0.445 ± 0.005	1.68 ± 0.02	0.94 ± 0.0
SGP(50)	9.7 ± 1.4	-1042.7 ± 5.2	41.4 ± 5.59	0.146 ± 0.012	0.299 ± 0.003	0.537 ± 0.003	0.416 ± 0.006	1.59 ± 0.01	0.94 ± 0.0
SGP(100)	14.4 ± 0.8	-1009.6 ± 1.2	9.86 ± 1.73	0.069 ± 0.006	0.285 ± 0.002	0.514 ± 0.004	0.395 ± 0.005	1.51 ± 0.02	0.94 ± 0.0
minVar	2.0 ± 0.2	-1025.8 ± 1.1	19.39 ± 1.78	0.101 ± 0.005	**0.282 ± 0.002**	**0.508 ± 0.005**	**0.39 ± 0.003**	**1.48 ± 0.02**	0.93 ± 0.0
GPoE	1.9 ± 0.1	-1025.8 ± 1.1	54.22 ± 1.64	0.162 ± 0.003	0.301 ± 0.002	0.535 ± 0.004	0.424 ± 0.006	1.6 ± 0.01	0.96 ± 0.0
BCM	1.9 ± 0.1	-1025.8 ± 1.1	257.61 ± 8.81	0.209 ± 0.005	0.313 ± 0.003	0.555 ± 0.006	0.422 ± 0.004	2.02 ± 0.04	0.82 ± 0.0
RBCM	1.9 ± 0.1	-1025.8 ± 1.1	38.35 ± 1.56	0.132 ± 0.003	0.295 ± 0.003	0.528 ± 0.005	0.408 ± 0.005	1.56 ± 0.02	0.92 ± 0.0
GRBCM	2.3 ± 0.2	-1048.9 ± 1.7	69.12 ± 6.48	0.196 ± 0.01	0.307 ± 0.004	0.551 ± 0.007	0.431 ± 0.006	1.64 ± 0.02	0.94 ± 0.0
**CPoE(1)**	2.1 ± 0.1	-1025.8 ± 1.1	12.18 ± 0.92	0.079 ± 0.003	0.284 ± 0.002	0.51 ± 0.004	0.393 ± 0.003	1.49 ± 0.02	0.94 ± 0.0
**CPoE(2)**	2.8 ± 0.1	-1010.1 ± 1.5	8.44 ± 0.66	0.066 ± 0.003	0.285 ± 0.002	0.512 ± 0.004	0.394 ± 0.004	1.5 ± 0.02	0.93 ± 0.0
**CPoE(3)**	3.1 ± 0.1	-1007.0 ± 1.5	7.83 ± 0.58	0.064 ± 0.002	0.285 ± 0.002	0.513 ± 0.004	0.394 ± 0.004	1.5 ± 0.02	0.93 ± 0.0
**CPoE(4)**	3.3 ± 0.1	-1004.8 ± 1.5	**7.59 ± 0.63**	**0.062 ± 0.003**	0.285 ± 0.002	0.513 ± 0.004	0.393 ± 0.004	1.5 ± 0.02	0.93 ± 0.0

Table F6: Results for dataset *mg*.

	time	LML	KL	ERR	CRPS	RMSE	ABSE	NLP	COV
fullGP	114.8 ± 4.3	-2113.6 ± 5.6	0.0 ± 0.0	0.0 ± 0.0	0.255 ± 0.005	0.471 ± 0.01	0.348 ± 0.007	1.3 ± 0.04	0.95 ± 0.0
SGP(50)	34.8 ± 4.8	-2319.6 ± 7.4	137.62 ± 7.41	0.259 ± 0.009	0.288 ± 0.005	0.531 ± 0.012	0.395 ± 0.007	1.57 ± 0.04	0.95 ± 0.0
SGP(100)	46.6 ± 6.1	-2242.4 ± 7.5	108.14 ± 6.24	0.229 ± 0.008	0.279 ± 0.005	0.514 ± 0.012	0.382 ± 0.007	1.5 ± 0.04	0.95 ± 0.0
SGP(150)	56.6 ± 6.8	-2205.9 ± 6.6	90.94 ± 6.01	0.21 ± 0.009	0.275 ± 0.005	0.508 ± 0.012	0.376 ± 0.007	1.47 ± 0.04	0.94 ± 0.0
minVar	7.2 ± 0.2	-2312.6 ± 6.8	63.58 ± 2.93	0.19 ± 0.01	0.272 ± 0.006	0.508 ± 0.016	0.374 ± 0.008	1.41 ± 0.04	0.95 ± 0.0
GPoE	7.2 ± 0.2	-2312.6 ± 6.8	98.01 ± 3.06	0.2 ± 0.013	0.279 ± 0.006	0.515 ± 0.02	0.378 ± 0.008	1.49 ± 0.03	0.97 ± 0.0
BCM	7.2 ± 0.2	-2312.6 ± 6.8	222.78 ± 4.12	0.2 ± 0.008	0.28 ± 0.007	0.511 ± 0.016	0.38 ± 0.008	1.75 ± 0.1	0.87 ± 0.01
RBCM	7.2 ± 0.2	-2312.6 ± 6.8	635.61 ± 21.61	0.194 ± 0.011	0.285 ± 0.007	0.513 ± 0.018	0.378 ± 0.008	2.54 ± 0.18	0.77 ± 0.01
GRBCM	6.5 ± 0.2	-2397.3 ± 6.2	105.64 ± 5.13	0.24 ± 0.008	0.284 ± 0.005	0.525 ± 0.012	0.391 ± 0.007	1.5 ± 0.04	0.95 ± 0.01
**CPoE(1)**	7.8 ± 0.2	-2316.1 ± 6.8	62.99 ± 2.94	0.186 ± 0.011	0.272 ± 0.006	0.507 ± 0.018	0.372 ± 0.008	1.41 ± 0.04	0.96 ± 0.0
**CPoE(2)**	10.6 ± 0.2	-2164.9 ± 6.7	36.45 ± 3.02	0.142 ± 0.011	0.264 ± 0.005	0.491 ± 0.015	**0.361 ± 0.008**	**1.36 ± 0.04**	0.95 ± 0.0
**CPoE(3)**	12.9 ± 0.2	-2165.9 ± 6.7	36.27 ± 2.99	0.141 ± 0.01	**0.263 ± 0.005**	0.49 ± 0.014	**0.361 ± 0.008**	**1.36 ± 0.04**	0.95 ± 0.0
**CPoE(4)**	14.9 ± 0.2	-2166.2 ± 6.7	**36.03 ± 3.0**	**0.14 ± 0.01**	**0.263 ± 0.005**	**0.489 ± 0.014**	**0.361 ± 0.008**	**1.36 ± 0.04**	0.95 ± 0.0

Table F7: Results for dataset *space*.

	time	LML	KL	ERR	CRPS	RMSE	ABSE	NLP	COV
fullGP	237.9 ± 12.2	-3722.3 ± 7.4	0.0 ± 0.0	0.0 ± 0.0	0.34 ± 0.005	0.635 ± 0.012	0.459 ± 0.006	1.92 ± 0.04	0.94 ± 0.0
SGP(20)	21.9 ± 2.5	-3785.3 ± 5.8	27.8 ± 4.1	0.15 ± 0.01	0.343 ± 0.004	0.635 ± 0.011	0.463 ± 0.005	1.93 ± 0.03	0.95 ± 0.0
SGP(50)	26.4 ± 3.6	-3758.7 ± 7.6	22.4 ± 3.9	0.14 ± 0.01	0.342 ± 0.004	0.633 ± 0.011	0.461 ± 0.006	1.93 ± 0.03	0.94 ± 0.0
SGP(100)	58.9 ± 7.0	-3746.9 ± 7.4	15.6 ± 3.5	0.11 ± 0.01	**0.34 ± 0.005**	**0.631 ± 0.012**	**0.457 ± 0.006**	1.92 ± 0.04	0.94 ± 0.0
minVar	6.4 ± 0.4	-3847.3 ± 7.2	25.1 ± 1.5	0.15 ± 0.0	0.346 ± 0.005	0.647 ± 0.013	0.466 ± 0.006	1.94 ± 0.04	0.94 ± 0.0
GPoE	6.3 ± 0.4	-3847.3 ± 7.2	50.3 ± 1.0	0.19 ± 0.0	0.353 ± 0.004	0.652 ± 0.011	0.478 ± 0.006	1.99 ± 0.02	0.96 ± 0.0
BCM	6.3 ± 0.3	-3847.3 ± 7.2	1838.2 ± 46.8	0.16 ± 0.0	0.373 ± 0.006	0.642 ± 0.011	0.473 ± 0.006	5.33 ± 0.24	0.67 ± 0.01
RBCM	6.3 ± 0.3	-3847.3 ± 7.2	1147.4 ± 64.8	0.12 ± 0.0	0.362 ± 0.006	0.638 ± 0.012	0.466 ± 0.006	4.01 ± 0.21	0.73 ± 0.01
GRBCM	7.6 ± 0.4	-3864.0 ± 7.6	36.4 ± 1.9	0.18 ± 0.0	0.353 ± 0.004	0.661 ± 0.011	0.477 ± 0.005	1.98 ± 0.03	0.94 ± 0.0
**CPoE(1)**	6.4 ± 0.4	-3848.6 ± 7.3	16.8 ± 0.6	0.12 ± 0.0	0.342 ± 0.004	0.638 ± 0.012	0.463 ± 0.005	1.92 ± 0.03	0.95 ± 0.0
**CPoE(2)**	7.5 ± 0.3	-3737.3 ± 7.0	8.1 ± 0.5	0.08 ± 0.0	0.341 ± 0.005	0.636 ± 0.012	0.463 ± 0.006	1.92 ± 0.04	0.94 ± 0.0
**CPoE(3)**	9.3 ± 0.5	-3736.5 ± 7.2	6.2 ± 0.6	0.07 ± 0.0	0.341 ± 0.005	0.636 ± 0.012	0.461 ± 0.006	1.92 ± 0.04	0.94 ± 0.0
**CPoE(4)**	10.4 ± 0.3	-3733.7 ± 7.0	**4.7 ± 0.5**	**0.06 ± 0.0**	**0.34 ± 0.005**	0.635 ± 0.012	0.46 ± 0.006	**1.91 ± 0.04**	0.94 ± 0.0

Table F8: Results for dataset *abalone*.

	time	LML	KL	ERR	CRPS	RMSE	ABSE	NLP	COV
fullGP	161.5 ± 3.6	-1232.1 ± 7.4	0.0 ± 0.0	0.0 ± 0.0	0.148 ± 0.001	0.267 ± 0.001	0.207 ± 0.001	0.17 ± 0.01	0.94 ± 0.0
SGP(100)	42.2 ± 6.5	-4033.6 ± 27.1	603.7 ± 9.4	0.4 ± 0.01	0.265 ± 0.003	0.476 ± 0.005	0.369 ± 0.004	1.35 ± 0.02	0.96 ± 0.0
SGP(200)	49.8 ± 3.3	-3141.3 ± 17.8	408.4 ± 3.7	0.29 ± 0.0	0.218 ± 0.001	0.392 ± 0.001	0.303 ± 0.001	0.96 ± 0.0	0.96 ± 0.0
SGP(300)	54.8 ± 2.2	-2732.8 ± 13.5	323.1 ± 5.0	0.25 ± 0.0	0.201 ± 0.001	0.363 ± 0.001	0.281 ± 0.001	0.8 ± 0.01	0.96 ± 0.0
minVar	9.3 ± 0.2	-2820.5 ± 9.0	211.0 ± 2.3	0.2 ± 0.0	0.183 ± 0.001	0.333 ± 0.001	0.256 ± 0.001	0.59 ± 0.01	0.94 ± 0.0
GPoE	9.4 ± 0.1	-2820.5 ± 9.0	342.3 ± 2.6	0.23 ± 0.0	0.202 ± 0.001	0.354 ± 0.002	0.278 ± 0.002	0.84 ± 0.01	0.99 ± 0.0
BCM	9.4 ± 0.1	-2820.5 ± 9.0	1629.2 ± 24.7	0.25 ± 0.0	0.218 ± 0.002	0.367 ± 0.002	0.278 ± 0.002	3.45 ± 0.07	0.64 ± 0.0
RBCM	9.4 ± 0.2	-2820.5 ± 9.0	939.3 ± 17.4	0.2 ± 0.0	0.193 ± 0.001	0.331 ± 0.002	0.253 ± 0.001	2.06 ± 0.05	0.71 ± 0.0
GRBCM	11.9 ± 0.2	-2981.3 ± 9.6	129.8 ± 3.0	0.14 ± 0.0	0.168 ± 0.001	0.303 ± 0.001	0.235 ± 0.001	0.43 ± 0.01	0.94 ± 0.0
**CPoE(1)**	9.2 ± 0.1	-2822.7 ± 8.9	152.4 ± 1.7	0.15 ± 0.0	0.17 ± 0.001	0.307 ± 0.001	0.237 ± 0.001	0.46 ± 0.0	0.97 ± 0.0
**CPoE(2)**	12.9 ± 0.1	-1811.2 ± 11.1	79.9 ± 1.3	0.11 ± 0.0	0.161 ± 0.001	0.29 ± 0.001	0.225 ± 0.001	0.33 ± 0.01	0.95 ± 0.0
**CPoE(3)**	19.8 ± 0.3	-1466.0 ± 9.9	46.9 ± 1.0	0.09 ± 0.0	0.155 ± 0.001	0.279 ± 0.001	0.217 ± 0.001	0.26 ± 0.01	0.95 ± 0.0
**CPoE(4)**	27.8 ± 0.2	-1363.8 ± 9.2	**32.8 ± 1.0**	**0.07 ± 0.0**	**0.153 ± 0.001**	**0.276 ± 0.001**	**0.215 ± 0.001**	**0.24 ± 0.01**	0.94 ± 0.0

Table F9: Results for dataset *kin*.

	time	LML	CRPS	RMSE	ABSE	NLP	COV
SGP(250)	77.7 ± 0.4	-4163.9 ± 23.7	0.207 ± 0.002	0.366 ± 0.004	0.282 ± 0.002	0.93 ± 0.01	0.98 ± 0.0
SGP(500)	112.1 ± 1.2	-3242.2 ± 12.6	0.183 ± 0.001	0.324 ± 0.002	0.252 ± 0.001	0.67 ± 0.01	0.98 ± 0.0
SGP(1000)	244.1 ± 2.9	-2534.7 ± 9.0	0.166 ± 0.001	0.294 ± 0.002	0.23 ± 0.001	0.46 ± 0.01	0.98 ± 0.0
minVar	14.4 ± 0.5	-3388.8 ± 7.9	0.173 ± 0.002	0.314 ± 0.004	0.242 ± 0.002	0.48 ± 0.02	0.94 ± 0.0
GPoE	14.4 ± 0.5	-3388.8 ± 7.9	0.193 ± 0.001	0.34 ± 0.003	0.267 ± 0.002	0.76 ± 0.01	0.99 ± 0.0
BCM	14.4 ± 0.5	-3388.8 ± 7.9	0.21 ± 0.001	0.35 ± 0.003	0.266 ± 0.002	3.6 ± 0.1	0.63 ± 0.0
RBCM	14.4 ± 0.5	-3388.8 ± 7.9	0.188 ± 0.001	0.318 ± 0.003	0.244 ± 0.002	2.39 ± 0.09	0.69 ± 0.0
GRBCM	16.5 ± 0.4	-3388.8 ± 7.9	0.164 ± 0.001	0.294 ± 0.003	0.229 ± 0.002	0.37 ± 0.02	0.94 ± 0.0
**CPoE(1)**	13.8 ± 0.2	-3393.9 ± 8.0	0.163 ± 0.001	0.292 ± 0.003	0.226 ± 0.002	0.38 ± 0.01	0.97 ± 0.0
**CPoE(2)**	18.9 ± 0.3	-2076.6 ± 12.9	0.155 ± 0.001	0.278 ± 0.002	0.217 ± 0.001	0.27 ± 0.01	0.95 ± 0.0
**CPoE(3)**	31.7 ± 0.6	-1655.2 ± 8.7	**0.151 ± 0.001**	**0.27 ± 0.002**	**0.211 ± 0.001**	**0.21 ± 0.01**	0.95 ± 0.0

Table F10: Results for dataset *kin2* for the stochastic versions.

	time	LML	CRPS	RMSE	ABSE	NLP	COV
SGP(250)	70.9 ± 3.7	-3905.6 ± 23.3	0.207 ± 0.002	0.373 ± 0.004	0.287 ± 0.002	0.85 ± 0.02	0.96 ± 0.0
SGP(500)	86.1 ± 1.8	-2968.6 ± 11.7	0.181 ± 0.001	0.325 ± 0.003	0.252 ± 0.001	0.57 ± 0.01	0.96 ± 0.0
SGP(1000)	143.6 ± 3.6	-2277.2 ± 8.6	0.162 ± 0.001	0.292 ± 0.002	0.225 ± 0.001	0.36 ± 0.01	0.96 ± 0.0
minVar	13.8 ± 0.2	-3384.5 ± 7.8	0.173 ± 0.002	0.314 ± 0.004	0.241 ± 0.002	0.48 ± 0.02	0.94 ± 0.0
GPoE	13.8 ± 0.2	-3384.5 ± 7.8	0.193 ± 0.001	0.34 ± 0.002	0.267 ± 0.002	0.75 ± 0.01	0.99 ± 0.0
BCM	13.8 ± 0.2	-3384.5 ± 7.8	0.209 ± 0.001	0.35 ± 0.003	0.266 ± 0.002	3.63 ± 0.07	0.63 ± 0.0
RBCM	13.8 ± 0.2	-3384.5 ± 7.8	0.187 ± 0.001	0.317 ± 0.003	0.243 ± 0.001	2.38 ± 0.06	0.69 ± 0.0
GRBCM	18.8 ± 0.4	-3608.7 ± 8.4	0.164 ± 0.001	0.294 ± 0.002	0.229 ± 0.002	0.38 ± 0.02	0.94 ± 0.0
**CPoE(1)**	16.2 ± 0.8	-3389.8 ± 8.0	0.162 ± 0.001	0.292 ± 0.003	0.225 ± 0.002	0.37 ± 0.01	0.97 ± 0.0
**CPoE(2)**	21.5 ± 0.7	-2071.4 ± 13.0	0.155 ± 0.001	0.278 ± 0.002	0.217 ± 0.001	0.26 ± 0.01	0.95 ± 0.0
**CPoE(3)**	34.3 ± 0.9	-1650.7 ± 8.3	**0.15 ± 0.001**	**0.27 ± 0.002**	**0.211 ± 0.001**	**0.21 ± 0.01**	0.94 ± 0.0

Table F11: Results for dataset *kin2* for the deterministic batch version.

	time	LML	CRPS	RMSE	ABSE	NLP	COV
SGP(250)	248.6 ± 0.6	-15182.0 ± 35.7	0.254 ± 0.003	0.48 ± 0.009	0.335 ± 0.004	1.42 ± 0.03	0.95 ± 0.0
SGP(500)	346.9 ± 3.4	-15074.6 ± 37.2	0.253 ± 0.003	0.478 ± 0.009	0.333 ± 0.004	1.41 ± 0.03	0.95 ± 0.0
SGP(1000)	727.6 ± 3.5	-14961.2 ± 31.4	0.252 ± 0.003	**0.476 ± 0.009**	0.332 ± 0.004	1.4 ± 0.03	0.95 ± 0.0
minVar	28.2 ± 1.0	-15387.4 ± 17.5	0.257 ± 0.003	0.491 ± 0.009	0.337 ± 0.005	1.42 ± 0.04	0.94 ± 0.0
GPoE	28.3 ± 1.0	-15387.4 ± 17.5	0.289 ± 0.003	0.534 ± 0.009	0.371 ± 0.004	1.64 ± 0.02	0.96 ± 0.0
BCM	28.5 ± 0.9	-15387.4 ± 17.5	0.321 ± 0.004	0.536 ± 0.01	0.373 ± 0.004	20.72 ± 1.0	0.45 ± 0.0
RBCM	28.5 ± 0.9	-15387.4 ± 17.5	0.303 ± 0.005	0.515 ± 0.01	0.358 ± 0.004	15.98 ± 0.9	0.51 ± 0.01
GRBCM	33.5 ± 1.2	-15387.4 ± 17.5	0.262 ± 0.003	0.499 ± 0.009	0.346 ± 0.004	1.44 ± 0.03	0.94 ± 0.0
**CPoE(1)**	24.5 ± 0.1	-15404.2 ± 17.8	0.259 ± 0.004	0.492 ± 0.01	0.335 ± 0.005	1.43 ± 0.04	0.95 ± 0.0
**CPoE(2)**	33.4 ± 0.2	-13645.5 ± 19.8	0.251 ± 0.003	0.479 ± 0.009	0.328 ± 0.004	1.36 ± 0.04	0.94 ± 0.0
**CPoE(3)**	52.0 ± 0.5	-13483.2 ± 15.6	**0.249 ± 0.004**	**0.476 ± 0.01**	**0.324 ± 0.004**	**1.34 ± 0.04**	0.94 ± 0.0

Table F12: Results for dataset *cadata*.

	time	LML	CRPS	RMSE	ABSE	NLP	COV
SGP(250)	473.4 ± 1.0	9370.3 ± 60.7	0.0746 ± 0.0005	0.1407 ± 0.0008	0.097 ± 0.001	-0.39 ± 0.01	0.95 ± 0.0
SGP(500)	730.1 ± 1.1	12112.0 ± 68.2	0.0695 ± 0.0003	0.1304 ± 0.001	0.09 ± 0.001	-0.49 ± 0.01	0.95 ± 0.0
SGP(1000)	1718.5 ± 1.8	16034.0 ± 91.6	0.0628 ± 0.0003	0.1172 ± 0.0009	0.081 ± 0.0	-0.64 ± 0.01	0.96 ± 0.0
minVar	71.3 ± 23.1	27128.2 ± 20.2	0.0516 ± 0.0008	0.1024 ± 0.0034	**0.067 ± 0.001**	**-1.88 ± 0.04**	0.93 ± 0.0
GPoE	71.4 ± 23.2	27128.2 ± 20.2	0.0862 ± 0.0004	0.1322 ± 0.0013	0.096 ± 0.001	-0.57 ± 0.01	1.0 ± 0.0
BCM	71.5 ± 23.2	27128.2 ± 20.2	0.095 ± 0.001	0.1544 ± 0.001	0.115 ± 0.001	7.86 ± 0.3	0.48 ± 0.01
RBCM	71.6 ± 23.2	27128.2 ± 20.2	0.0726 ± 0.0009	0.1196 ± 0.0013	0.086 ± 0.001	11.45 ± 0.47	0.5 ± 0.01
GRBCM	84.6 ± 23.0	27128.2 ± 20.2	0.06 ± 0.0007	0.1102 ± 0.001	0.079 ± 0.001	-0.52 ± 0.08	0.79 ± 0.01
**CPoE(1)**	45.4 ± 0.2	-41213.2 ± 883.2	0.0516 ± 0.0005	0.0998 ± 0.0019	0.067 ± 0.001	-1.86 ± 0.02	0.96 ± 0.0
**CPoE(2)**	67.3 ± 0.4	-37867.5 ± 911.8	0.0509 ± 0.0006	0.0977 ± 0.0015	0.067 ± 0.001	-1.8 ± 0.02	0.93 ± 0.0
**CPoE(3)**	134.3 ± 1.2	-37204.6 ± 949.1	**0.0507 ± 0.0005**	**0.0975 ± 0.0011**	**0.067 ± 0.001**	-1.78 ± 0.02	0.92 ± 0.0

Table F13: Results for dataset *sarcos*.

	time	LML	CRPS	RMSE	ABSE	NLP	COV
SGP(250)	443.2 ± 2.1	-53395.2 ± 80.2	0.334 ± 0.004	0.59 ± 0.008	0.475 ± 0.007	1.77 ± 0.02	0.96 ± 0.0
SGP(500)	632.9 ± 2.7	-52988.7 ± 58.9	0.329 ± 0.005	0.582 ± 0.008	0.467 ± 0.007	1.75 ± 0.02	0.96 ± 0.0
SGP(1000)	1362.5 ± 4.8	-52592.1 ± 46.9	0.325 ± 0.005	**0.575 ± 0.008**	0.459 ± 0.007	1.74 ± 0.02	0.96 ± 0.0
minVar	45.8 ± 1.0	-39976.0 ± 22.6	0.294 ± 0.003	0.607 ± 0.006	0.387 ± 0.003	1.4 ± 0.03	0.93 ± 0.0
GPoE	45.6 ± 0.8	-39976.0 ± 22.6	0.302 ± 0.003	0.6 ± 0.006	0.409 ± 0.005	1.43 ± 0.02	0.97 ± 0.0
BCM	45.7 ± 0.9	-39976.0 ± 22.6	0.316 ± 0.005	0.615 ± 0.009	0.416 ± 0.007	2.47 ± 0.1	0.82 ± 0.01
RBCM	45.7 ± 0.9	-39976.0 ± 22.6	0.312 ± 0.004	0.647 ± 0.008	0.425 ± 0.006	1.61 ± 0.05	0.91 ± 0.01
GRBCM	59.4 ± 1.1	-39976.0 ± 22.6	0.31 ± 0.004	0.642 ± 0.008	0.421 ± 0.005	1.5 ± 0.04	0.92 ± 0.01
**CPoE(1)**	45.1 ± 0.3	-40075.2 ± 22.1	0.289 ± 0.003	0.596 ± 0.006	0.38 ± 0.004	**1.35 ± 0.03**	0.94 ± 0.0
**CPoE(2)**	70.3 ± 0.6	-39571.2 ± 65.5	0.287 ± 0.004	0.589 ± 0.007	0.38 ± 0.005	1.36 ± 0.03	0.93 ± 0.0
**CPoE(3)**	123.8 ± 1.4	-39439.5 ± 98.8	**0.282 ± 0.004**	**0.575 ± 0.008**	**0.372 ± 0.006**	1.37 ± 0.04	0.92 ± 0.01

Table F14: Results for dataset *casp*.

	time	RMSE	ABSE	CRPS	COV
fullGP-SE	12.52 ± 0.98	0.311 ± 0.011	0.218 ± 0.005	0.162 ± 0.004	0.92 ± 0.01
fullGP-FLEX	28.23 ± 7.99	0.254 ± 0.01	0.169 ± 0.005	0.128 ± 0.004	0.94 ± 0.01
**CPoE(1)-SE**	1.97 ± 0.06	0.333 ± 0.011	0.236 ± 0.006	0.175 ± 0.004	0.92 ± 0.01
**CPoE(2)-SE**	2.39 ± 0.06	0.326 ± 0.011	0.231 ± 0.006	0.171 ± 0.004	0.91 ± 0.01
**CPoE(3)-SE**	2.77 ± 0.11	0.323 ± 0.01	0.23 ± 0.005	0.17 ± 0.004	0.91 ± 0.01
**CPoE(4)-SE**	2.89 ± 0.06	0.324 ± 0.011	0.231 ± 0.005	0.171 ± 0.004	0.91 ± 0.01
**CPoE(1)-FLEX**	16.37 ± 1.11	0.266 ± 0.009	0.175 ± 0.005	0.133 ± 0.004	0.93 ± 0.01
**CPoE(2)-FLEX**	17.06 ± 1.14	0.259 ± 0.01	0.172 ± 0.005	0.13 ± 0.004	0.94 ± 0.01
**CPoE(3)-FLEX**	17.35 ± 1.09	0.255 ± 0.01	0.171 ± 0.005	0.129 ± 0.004	0.94 ± 0.01
**CPoE(4)-FLEX**	17.7 ± 1.1	**0.255 ± 0.01**	**0.171 ± 0.005**	**0.129 ± 0.004**	0.95 ± 0.01
MLP(100-100)	4.52 ± 0.39	0.289 ± 0.011	0.204 ± 0.005	0.0 ± 0.0	0.0 ± 0.0
MLP(500-500)	10.73 ± 0.62	0.292 ± 0.008	0.208 ± 0.004	0.0 ± 0.0	0.0 ± 0.0
MLP(100-100-100)	4.14 ± 0.18	0.285 ± 0.011	0.2 ± 0.006	0.0 ± 0.0	0.0 ± 0.0
XGboost	32.16 ± 1.87	0.323 ± 0.008	0.235 ± 0.007	0.0 ± 0.0	0.0 ± 0.0
LinReg	0.01 ± 0.0	0.626 ± 0.01	0.492 ± 0.009	0.0 ± 0.0	0.0 ± 0.0

Table F15: Comparison with non-GP methods for dataset *concrete*. Best method (besides full GP) is indicated in bold.

	time	RMSE	ABSE	CRPS	COV
fullGP-SE	38.89 ± 1.93	0.511 ± 0.004	0.39 ± 0.005	0.283 ± 0.002	0.94 ± 0.0
fullGP-FLEX	161.63 ± 12.31	0.509 ± 0.004	0.388 ± 0.004	0.282 ± 0.002	0.93 ± 0.0
**CPoE(1)-SE**	2.37 ± 0.14	**0.508 ± 0.005**	0.39 ± 0.003	0.282 ± 0.002	0.94 ± 0.01
**CPoE(2)-SE**	2.81 ± 0.11	0.512 ± 0.004	0.393 ± 0.004	0.285 ± 0.002	0.93 ± 0.0
**CPoE(3)-SE**	3.13 ± 0.09	0.513 ± 0.004	0.394 ± 0.004	0.285 ± 0.002	0.93 ± 0.0
**CPoE(4)-SE**	3.38 ± 0.08	0.513 ± 0.004	0.393 ± 0.004	0.284 ± 0.002	0.93 ± 0.0
**CPoE(1)-FLEX**	24.06 ± 2.31	0.511 ± 0.005	**0.388 ± 0.004**	**0.281 ± 0.003**	0.93 ± 0.01
**CPoE(2)-FLEX**	24.68 ± 2.31	0.515 ± 0.005	0.391 ± 0.005	0.283 ± 0.003	0.93 ± 0.01
**CPoE(3)-FLEX**	25.37 ± 2.31	0.516 ± 0.005	0.392 ± 0.005	0.284 ± 0.004	0.93 ± 0.01
**CPoE(4)-FLEX**	26.14 ± 2.35	0.516 ± 0.005	0.391 ± 0.005	0.284 ± 0.004	0.93 ± 0.01
MLP(100-100)	3.83 ± 0.35	0.525 ± 0.004	0.398 ± 0.006	0.0 ± 0.0	0.0 ± 0.0
MLP(500-500)	11.97 ± 0.8	0.522 ± 0.004	0.397 ± 0.006	0.0 ± 0.0	0.0 ± 0.0
MLP(100-100-100)	4.84 ± 0.36	0.531 ± 0.006	0.403 ± 0.007	0.0 ± 0.0	0.0 ± 0.0
XGboost	0.65 ± 0.01	0.545 ± 0.008	0.405 ± 0.008	0.0 ± 0.0	0.0 ± 0.0
LinReg	0.01 ± 0.0	0.633 ± 0.006	0.509 ± 0.006	0.0 ± 0.0	0.0 ± 0.0

Table F16: Comparison with non-GP methods for dataset *mg*. Best method (besides full GP) is indicated in bold.

	time	RMSE	ABSE	CRPS	COV
fullGP-SE	137.76 ± 5.07	0.471 ± 0.01	0.348 ± 0.007	0.255 ± 0.005	0.95 ± 0.0
fullGP-FLEX	700.27 ± 255.47	0.455 ± 0.014	0.328 ± 0.007	0.238 ± 0.004	0.94 ± 0.0
**CPoE(1)-SE**	11.64 ± 0.59	0.506 ± 0.017	0.371 ± 0.008	0.27 ± 0.005	0.95 ± 0.0
**CPoE(2)-SE**	14.86 ± 0.53	0.49 ± 0.015	0.36 ± 0.008	0.263 ± 0.005	0.95 ± 0.0
**CPoE(3)-SE**	18.78 ± 0.56	0.489 ± 0.014	0.361 ± 0.008	0.263 ± 0.005	0.95 ± 0.0
**CPoE(4)-SE**	22.57 ± 0.56	0.489 ± 0.014	0.361 ± 0.008	0.263 ± 0.005	0.95 ± 0.0
**CPoE(1)-FLEX**	72.08 ± 4.54	0.631 ± 0.121	0.347 ± 0.012	0.252 ± 0.011	0.95 ± 0.01
**CPoE(2)-FLEX**	76.73 ± 4.55	0.446 ± 0.019	0.324 ± 0.005	0.236 ± 0.004	0.95 ± 0.0
**CPoE(3)-FLEX**	82.48 ± 4.6	0.444 ± 0.02	0.321 ± 0.005	0.234 ± 0.004	0.95 ± 0.0
**CPoE(4)-FLEX**	86.95 ± 4.57	**0.449 ± 0.023**	**0.324 ± 0.006**	**0.236 ± 0.005**	0.95 ± 0.01
MLP(100-100)	7.22 ± 0.68	0.482 ± 0.011	0.355 ± 0.008	0.0 ± 0.0	0.0 ± 0.0
MLP(500-500)	33.31 ± 2.41	0.475 ± 0.011	0.353 ± 0.008	0.0 ± 0.0	0.0 ± 0.0
MLP(100-100-100)	9.87 ± 1.13	0.476 ± 0.01	0.351 ± 0.006	0.0 ± 0.0	0.0 ± 0.0
XGboost	0.72 ± 0.01	0.543 ± 0.026	0.386 ± 0.007	0.0 ± 0.0	0.0 ± 0.0
LinReg	0.01 ± 0.0	0.645 ± 0.014	0.485 ± 0.008	0.0 ± 0.0	0.0 ± 0.0

Table F17: Comparison with non-GP methods for dataset *space*. Best method (besides full GP) is indicated in bold.

	time	RMSE	ABSE	CRPS	COV
fullGP-SE	327.61 ± 23.85	0.635 ± 0.012	0.459 ± 0.006	0.34 ± 0.005	0.94 ± 0.0
fullGP-FLEX	1008.11 ± 165.51	0.638 ± 0.013	0.461 ± 0.007	0.34 ± 0.005	0.94 ± 0.0
**CPoE(1)-SE**	8.92 ± 0.5	0.637 ± 0.012	0.459 ± 0.006	0.34 ± 0.005	0.94 ± 0.0
**CPoE(2)-SE**	10.44 ± 0.41	0.634 ± 0.012	0.458 ± 0.006	0.34 ± 0.005	0.94 ± 0.0
**CPoE(3)-SE**	12.62 ± 0.39	0.634 ± 0.012	0.458 ± 0.006	0.34 ± 0.005	0.94 ± 0.0
**CPoE(4)-SE**	16.35 ± 0.97	**0.635 ± 0.012**	**0.459 ± 0.006**	**0.34 ± 0.005**	0.94 ± 0.0
**CPoE(1)-FLEX**	130.92 ± 10.31	0.687 ± 0.019	0.487 ± 0.01	0.365 ± 0.009	0.96 ± 0.0
**CPoE(2)-FLEX**	133.52 ± 10.34	0.669 ± 0.016	0.48 ± 0.01	0.355 ± 0.007	0.95 ± 0.0
**CPoE(3)-FLEX**	137.64 ± 10.23	0.659 ± 0.014	0.474 ± 0.009	0.35 ± 0.006	0.95 ± 0.0
**CPoE(4)-FLEX**	141.31 ± 10.28	0.655 ± 0.013	0.471 ± 0.008	0.348 ± 0.006	0.94 ± 0.0
MLP(100-100)	19.19 ± 3.86	0.652 ± 0.014	0.469 ± 0.008	0.0 ± 0.0	0.0 ± 0.0
MLP(500-500)	166.5 ± 17.02	0.761 ± 0.012	0.53 ± 0.008	0.0 ± 0.0	0.0 ± 0.0
MLP(100-100-100)	33.85 ± 2.41	0.762 ± 0.017	0.527 ± 0.009	0.0 ± 0.0	0.0 ± 0.0
XGboost	0.74 ± 0.04	0.65 ± 0.011	0.466 ± 0.005	0.0 ± 0.0	0.0 ± 0.0
LinReg	0.02 ± 0.0	0.66 ± 0.009	0.488 ± 0.005	0.0 ± 0.0	0.0 ± 0.0

Table F18: Comparison with non-GP methods for dataset *abalone*. Best method (besides full GP) is indicated in bold.

	time	RMSE	ABSE	CRPS	COV
fullGP-SE	178.61 ± 3.76	0.267 ± 0.001	0.207 ± 0.001	0.148 ± 0.001	0.94 ± 0.0
fullGP-FLEX	714.07 ± 18.51	0.28 ± 0.001	0.215 ± 0.001	0.154 ± 0.001	0.94 ± 0.0
**CPoE(1)-SE**	15.92 ± 0.77	0.31 ± 0.001	0.239 ± 0.001	0.172 ± 0.001	0.96 ± 0.0
**CPoE(2)-SE**	20.65 ± 0.57	0.292 ± 0.001	0.226 ± 0.001	0.162 ± 0.001	0.95 ± 0.0
**CPoE(3)-SE**	30.6 ± 1.03	0.28 ± 0.001	0.218 ± 0.001	0.155 ± 0.001	0.94 ± 0.0
**CPoE(4)-SE**	45.01 ± 1.33	**0.276 ± 0.001**	**0.215 ± 0.001**	**0.153 ± 0.001**	0.94 ± 0.0
**CPoE(1)-FLEX**	64.24 ± 2.05	0.334 ± 0.001	0.256 ± 0.001	0.185 ± 0.001	0.97 ± 0.0
**CPoE(2)-FLEX**	69.43 ± 2.02	0.315 ± 0.001	0.241 ± 0.001	0.174 ± 0.001	0.96 ± 0.0
**CPoE(3)-FLEX**	78.69 ± 2.1	0.303 ± 0.002	0.232 ± 0.001	0.167 ± 0.001	0.95 ± 0.0
**CPoE(4)-FLEX**	90.09 ± 2.11	0.297 ± 0.002	0.228 ± 0.001	0.164 ± 0.001	0.95 ± 0.0
MLP(100-100)	21.77 ± 1.0	0.287 ± 0.001	0.223 ± 0.001	0.0 ± 0.0	0.0 ± 0.0
MLP(500-500)	100.1 ± 5.9	0.284 ± 0.001	0.221 ± 0.001	0.0 ± 0.0	0.0 ± 0.0
MLP(100-100-100)	33.92 ± 2.19	0.299 ± 0.002	0.233 ± 0.001	0.0 ± 0.0	0.0 ± 0.0
XGboost	0.9 ± 0.05	0.667 ± 0.003	0.528 ± 0.002	0.0 ± 0.0	0.0 ± 0.0
LinReg	0.04 ± 0.0	0.765 ± 0.002	0.613 ± 0.002	0.0 ± 0.0	0.0 ± 0.0

Table F19: Comparison with non-GP methods for dataset *kin*. Best method (besides full GP) is indicated in bold.

	time	RMSE	ABSE	CRPS	COV
**CPoE(1)-SE**	73.17 ± 3.12	0.099 ± 0.002	0.066 ± 0.001	0.051 ± 0.001	0.95 ± 0.0
**CPoE(2)-SE**	107.82 ± 6.0	0.1 ± 0.002	0.068 ± 0.001	0.051 ± 0.001	0.93 ± 0.0
**CPoE(3)-SE**	197.54 ± 7.42	0.099 ± 0.001	0.069 ± 0.001	0.052 ± 0.0	0.92 ± 0.0
**CPoE(1)-FLEX**	205.74 ± 5.65	0.094 ± 0.002	0.062 ± 0.001	0.048 ± 0.0	0.95 ± 0.0
**CPoE(2)-FLEX**	244.57 ± 7.19	0.094 ± 0.002	0.061 ± 0.001	0.048 ± 0.0	0.94 ± 0.0
**CPoE(3)-FLEX**	346.93 ± 11.4	**0.092 ± 0.002**	**0.061 ± 0.001**	**0.047 ± 0.0**	0.94 ± 0.0
MLP(100-100)	64.83 ± 4.7	0.117 ± 0.001	0.084 ± 0.001	0.0 ± 0.0	0.0 ± 0.0
MLP(500-500)	407.75 ± 17.83	0.097 ± 0.001	0.069 ± 0.001	0.0 ± 0.0	0.0 ± 0.0
MLP(100-100-100)	104.19 ± 8.83	0.106 ± 0.001	0.076 ± 0.001	0.0 ± 0.0	0.0 ± 0.0
XGboost	4.72 ± 0.45	0.251 ± 0.002	0.183 ± 0.002	0.0 ± 0.0	0.0 ± 0.0
LinReg	0.28 ± 0.04	0.27 ± 0.003	0.193 ± 0.002	0.0 ± 0.0	0.0 ± 0.0

Table F20: Comparison with non-GP methods for dataset *sarcos*. Best method (besides full GP) is indicated in bold.

	time	RMSE	ABSE	CRPS	COV
**CPoE(1)-SE**	68.54 ± 1.67	0.597 ± 0.006	0.381 ± 0.004	0.289 ± 0.003	0.94 ± 0.0
**CPoE(2)-SE**	102.61 ± 1.54	0.59 ± 0.007	0.38 ± 0.005	0.288 ± 0.004	0.93 ± 0.01
**CPoE(3)-SE**	175.91 ± 3.32	0.59 ± 0.009	0.382 ± 0.006	0.289 ± 0.004	0.92 ± 0.01
**CPoE(1)-FLEX**	215.81 ± 4.6	0.525 ± 0.006	0.339 ± 0.003	0.261 ± 0.002	0.95 ± 0.0
**CPoE(2)-FLEX**	895.7 ± 563.48	0.522 ± 0.005	0.336 ± 0.003	0.259 ± 0.002	0.94 ± 0.0
**CPoE(3)-FLEX**	353.4 ± 4.53	**0.522 ± 0.005**	**0.336 ± 0.003**	**0.259 ± 0.002**	0.93 ± 0.0
MLP(100-100)	397.14 ± 34.0	0.591 ± 0.006	0.426 ± 0.006	0.0 ± 0.0	0.0 ± 0.0
MLP(500-500)	4710.19 ± 810.95	0.577 ± 0.015	0.399 ± 0.009	0.0 ± 0.0	0.0 ± 0.0
MLP(100-100-100)	639.71 ± 44.16	0.585 ± 0.006	0.406 ± 0.005	0.0 ± 0.0	0.0 ± 0.0
XGboost	2.55 ± 0.04	0.767 ± 0.007	0.615 ± 0.007	0.0 ± 0.0	0.0 ± 0.0
LinReg	0.14 ± 0.0	0.854 ± 0.009	0.714 ± 0.008	0.0 ± 0.0	0.0 ± 0.0

Table F21: Comparison with non-GP methods for dataset *casp*. Best method (besides full GP) is indicated in bold.

	concrete	mg	space	abalone	kin	cadata	sarcos	casp
fullGP-SE	0.218	0.39	0.348	0.459	0.207			
fullGP-FLEX	0.169	0.388	0.328	0.461	0.215			
**CPoE(1)-SE**	0.236	0.39	0.371	0.459	0.239	0.323	0.066	0.381
**CPoE(2)-SE**	0.231	0.393	0.36	0.458	0.226	0.318	0.068	0.38
**CPoE(3)-SE**	0.23	0.394	0.361	**0.458**	**0.218**	0.317	0.069	0.382
**CPoE(1)-FLEX**	0.175	**0.388**	0.347	0.487	0.256	0.288	0.062	0.339
**CPoE(2)-FLEX**	0.172	0.391	0.324	0.48	0.241	0.277	0.061	0.336
**CPoE(3)-FLEX**	**0.171**	0.392	**0.321**	0.474	0.232	**0.273**	**0.061**	**0.336**
MLP(100-100)	0.204	0.398	0.355	0.469	0.223	0.297	0.084	0.426
MLP(500-500)	0.208	0.397	0.353	0.53	0.221	0.312	0.069	0.399
MLP(100-100-100)	0.2	0.403	0.351	0.527	0.233	0.316	0.076	0.406
XGboost	0.235	0.405	0.386	0.466	0.528	0.329	0.183	0.615
LinReg	0.492	0.509	0.485	0.488	0.613	0.439	0.193	0.714

Table F22: Comparison for non-GP methods regarding *ABSE*. Best method (besides full GP) is indicated in bold.

	concrete	mg	space	abalone	kin	cadata	sarcos	casp
fullGP-SE	12.5	38.9	137.8	327.6	178.6			
fullGP-FLEX	28.2	161.6	700.3	1008.1	714.1			
**CPoE(1)-SE**	2.0	2.4	11.6	8.9	15.9	84.1	73.2	68.5
**CPoE(2)-SE**	2.4	2.8	14.9	10.4	20.6	118.2	107.8	102.6
**CPoE(3)-SE**	2.8	3.1	18.8	12.6	30.6	246.0	197.5	175.9
**CPoE(1)-FLEX**	16.4	24.1	72.1	130.9	64.2	159.9	205.7	215.8
**CPoE(2)-FLEX**	17.1	24.7	76.7	133.5	69.4	195.0	244.6	895.7
**CPoE(3)-FLEX**	17.4	25.4	82.5	137.6	78.7	274.6	346.9	353.4
MLP(100-100)	4.5	3.8	7.2	19.2	21.8	82.9	64.8	397.1
MLP(500-500)	10.7	12.0	33.3	166.5	100.1	925.6	407.7	4710.2
MLP(100-100-100)	4.1	4.8	9.9	33.8	33.9	195.3	104.2	639.7
XGboost	32.2	0.6	0.7	0.7	0.9	1.2	4.7	2.6
LinReg	0.0	0.0	0.0	0.0	0.0	0.1	0.3	0.1

Table F23: Comparison for non-GP methods regarding *time* in seconds. Note that, GPs provide predictive uncertainty estimates, which is a much harder task. Therefore, these computational times for competitive accuracy are very reasonable in our opinion.
